# Comparative effectiveness of contact tracing interventions in the context of the COVID-19 pandemic: a systematic review

**DOI:** 10.1007/s10654-023-00963-z

**Published:** 2023-02-16

**Authors:** Francisco Pozo-Martin, Miguel Angel Beltran Sanchez, Sophie Alice Müller, Viorela Diaconu, Kilian Weil, Charbel El Bcheraoui

**Affiliations:** 1 Evidence-based Public Health Unit, Centre for International Health Protection, Robert Koch Institute, Nordufer 20, 13353 Berlin, Germany; 2 Independent researcher, Valencia, Spain; 3 Centre for International Health Protection, Robert Koch Institute, Nordufer 20, 13353 Berlin, Germany

**Keywords:** Non-pharmaceutical interventions, Contact tracing, Systematic review, Effectiveness

## Abstract

Contact tracing is a non-pharmaceutical intervention (NPI) widely used in the control of the COVID-19 pandemic. Its effectiveness may depend on a number of factors including the proportion of contacts traced, delays in tracing, the mode of contact tracing (e.g. forward, backward or bidirectional contact training), the types of contacts who are traced (e.g. contacts of index cases or contacts of contacts of index cases), or the setting where contacts are traced (e.g. the household or the workplace). We performed a systematic review of the evidence regarding the comparative effectiveness of contact tracing interventions. 78 studies were included in the review, 12 observational (ten ecological studies, one retrospective cohort study and one pre-post study with two patient cohorts) and 66 mathematical modelling studies. Based on the results from six of the 12 observational studies, contact tracing can be effective at controlling COVID-19. Two high quality ecological studies showed the incremental effectiveness of adding digital contact tracing to manual contact tracing. One ecological study of intermediate quality showed that increases in contact tracing were associated with a drop in COVID-19 mortality, and a pre-post study of acceptable quality showed that prompt contact tracing of contacts of COVID-19 case clusters / symptomatic individuals led to a reduction in the reproduction number R. Within the seven observational studies exploring the effectiveness of contact tracing in the context of the implementation of other non-pharmaceutical interventions, contact tracing was found to have an effect on COVID-19 epidemic control in two studies and not in the remaining five studies. However, a limitation in many of these studies is the lack of description of the extent of implementation of contact tracing interventions. Based on the results from the mathematical modelling studies, we identified the following highly effective policies: (1) manual contact tracing with high tracing coverage and either medium-term immunity, highly efficacious isolation/quarantine and/ or physical distancing (2) hybrid manual and digital contact tracing with high app adoption with highly effective isolation/ quarantine and social distancing, (3) secondary contact tracing, (4) eliminating contact tracing delays, (5) bidirectional contact tracing, (6) contact tracing with high coverage in reopening educational institutions. We also highlighted the role of social distancing to enhance the effectiveness of some of these interventions in the context of 2020 lockdown reopening. While limited, the evidence from observational studies shows a role for manual and digital contact tracing in controlling the COVID-19 epidemic. More empirical studies accounting for the extent of contact tracing implementation are required.

**Supplementary Information:**

The online version contains supplementary material available at 10.1007/s10654-023-00963-z.

## Introduction

By January 5, 2022, the SARS-CoV-2 virus had reportedly infected more than 290.6 million people globally and caused more than 5.4 million deaths [1]. By December 9, 2021, 12 months after the first COVID-19 vaccine became available, only 59% of the world population and 8.9% of the population in low-income countries had received one dose of any COVID-19 vaccine [2]. In the absence of affordable and widely available treatments, governments still rely on non-pharmaceutical interventions (NPIs) to control COVID-19 transmission, morbidity and mortality. Contact tracing is the process of identifying and obtaining information from individuals who have been in long enough contact with other infected individuals (in this context, with other individuals infected with the SARS-CoV-2 virus). Contact tracing can be manual or digital. The former typically relies on the identification of contacts via interviews with cases followed by phone calls to contacts of these cases, while the latter relies on the use of smartphone-based apps to automatically store and report contact information via Bluetooth technology [3]. Once contacts are traced they are warned of their status and either quarantined and/or tested and, if testing positive, isolated and possibly treated. Contact tracing is a widely used intervention to contain outbreaks and one of a wide set of NPIs currently available to policy makers. It is a much less disruptive NPI than lockdown-type policies such as restrictions on gatherings, work closing or stay-at-home requirements. The mechanism by which contact tracing is effective (i.e. has an impact on morbidity and mortality) is by identifying contacts of the index case who have been exposed to the pathogen. As mentioned, these contacts can then be placed in quarantine, or tested for the pathogen and, if testing positive, isolated and maybe treated. With these interventions, onward transmission of the infectious agent is reduced. Contact tracing has been successfully used to control the COVID-19 pandemic in countries like Singapore, South Korea and China [4]. However, in other countries it has not worked so well. For example, the United Kingdom’s NHS Test and Trace programme has not been as effective at reducing COVID-19 transmission as was originally hoped [5]. In the United States, many states’ testing-tracing efforts after reopening were met with surges in case counts [6]. COVID-19 is a quite infectious disease which affects the whole population and which is transmitted by both symptomatic and asymptomatic individuals. In this context, the effectiveness of contact tracing interventions may vary, *inter alia*, based on a number of factors. For example, on the proportion of contacts who are traced (i.e. the contact tracing coverage) [7]; on the delays in tracing [8]; on the mode of contact tracing – for example, forward contact tracing (i.e. tracing the contacts of a known case), backward contact tracing (tracing the index case in a chain of contacts), or bidirectional contact tracing (i.e. both forward and backward contact tracing) [9, 10]; on whether only contacts of known cases are traced (primary or first order contact tracing) or contacts of contacts of known cases are traced (secondary or second order contact tracing) [11]; or on the setting where contacts are traced – e.g. household or workplace contacts [12].

In order to support policy makers in making decisions about whether, and if so what types of contact tracing interventions to implement to contain the COVID-19 pandemic, we performed a systematic review of the evidence regarding the comparative effectiveness of contact tracing interventions in the particular context of COVID-19 transmission.

## Methods

In this systematic review, we followed the Preferred Reporting Items for Systematic Reviews and Meta-analyses (PRISMA) statement [13]. On June 26, 2021, we searched Embase (including Medline resources) for published peer reviewed studies. On July 7, 2021, we searched medRxiv for preprints. We restricted the search to articles available in the English language from January 1, 2020. The search strategies for both databases are available in Annex 1 in the supplementary information file.

Studies were included in the review if they:

- Assessed the effectiveness of contact tracing interventions in terms of any health outcomes (e.g. morbidity, mortality) in the context of COVID-19.
- Were empirical (i.e. either observational, experimental or quasi-experimental) or mathematical modelling studies.
- Compared the effectiveness of two or more contact tracing interventions or compared the effectiveness of a contact tracing intervention to no contact tracing.

Studies were excluded if they:

- Did not assess the direct link between specific contact tracing interventions and a health outcome (for example, if they explored only testing or quarantining of contacts).
- Evaluated the cost-effectiveness rather than the effectiveness of contact tracing interventions.

We assessed the quality of the empirical studies with two different tools. For ecological studies, we used a risk of bias tool developed by Dufault et al. [14] which has been previously adapted in several systematic reviews [15–17]. This tool evaluates the study quality in the following three domains: study design, statistical methodology and reporting (for details, please see Annex 2.1 in the supplementary information file). For cohort studies, we used the Scottish Intercollegiate Guidelines Network (SIGN) cohort study critical appraisal tool [18]. 

To assess the quality of all studies that were based on mathematical models, we used an original framework informed by previous Cochrane reviews of similar studies, and developed by Anglemyer et al. [19] and Nussbaumer-Streit et al. [20]. Table 1 shows the criteria that we employed in the quality assessment, where each criterion was assigned a range of possible scores.

**Table 1:** Criteria used in assessing the quality of studies using mathematical models

Criteria	Scores
1. Is the model transparently described?	Yes = 1, No = 0
2. Are the parameters and their sources fully described?	Yes = 1, No = 0
3. Are sensitivity analyses performed on key model assumptions?	Yes = 1, No = 0
4. Does the model distinguish between different categories of infectiousness?	Yes = 1, No = 0
5. Is the model an individual-based simulation?	Yes = 3, No = 0
6. Does the model include social mixing or a multi-layer network?	Yes (multi-layer network) = 2 Yes (social mixing) = 1 No = 0

The first three criteria in Table 1 were included as key areas indicating the risk of bias after reviewing the modelling and reporting recommendations of the Society for Medical Decision Making (SMDM) and the International Society for Pharmacoeconomics and Outcomes (ISPOR) [21]. The last three criteria in Table 1 were risk of bias criteria based on the models’ realistic representation of SARS-CoV-2 disease transmission. As in reality Sars-CoV-2 transmission occurs at different rates from both symptomatic and asymptomatic individuals and also between different age groups, a model is more realistic if it distinguishes between different categories of infectiousness. Since disease transmission in reality occurs between individuals, a model is more realistic if it simulates infectiousness at the individual level rather than at the aggregate cohort level. As social mixing between individuals occurs in reality at a different rate in different contexts (e.g. in the household versus in the workplace), a model is more realistic if it represents the contacts of individuals either by distinguishing between social mixing for different social groups or for different networks of individuals. The maximum possible quality score for any given study was nine points. We excluded from the analysis any study which scored five points or less.

Study screening and selection was performed by five reviewers (FPM, MABS, KW, SAM, VD). Data extraction was performed by four reviewers (FPM, KW, SAM, VD). Another reviewer (MABS) screened a random selection of 10% of the total records and all the records that were selected by abstract. Two reviewers (FPM and MABS) independently assessed the quality of the studies. Disagreements between reviewers were solved by arbitration by a third reviewer (CEB).

## Results

### Overview

**Fig. 1:**
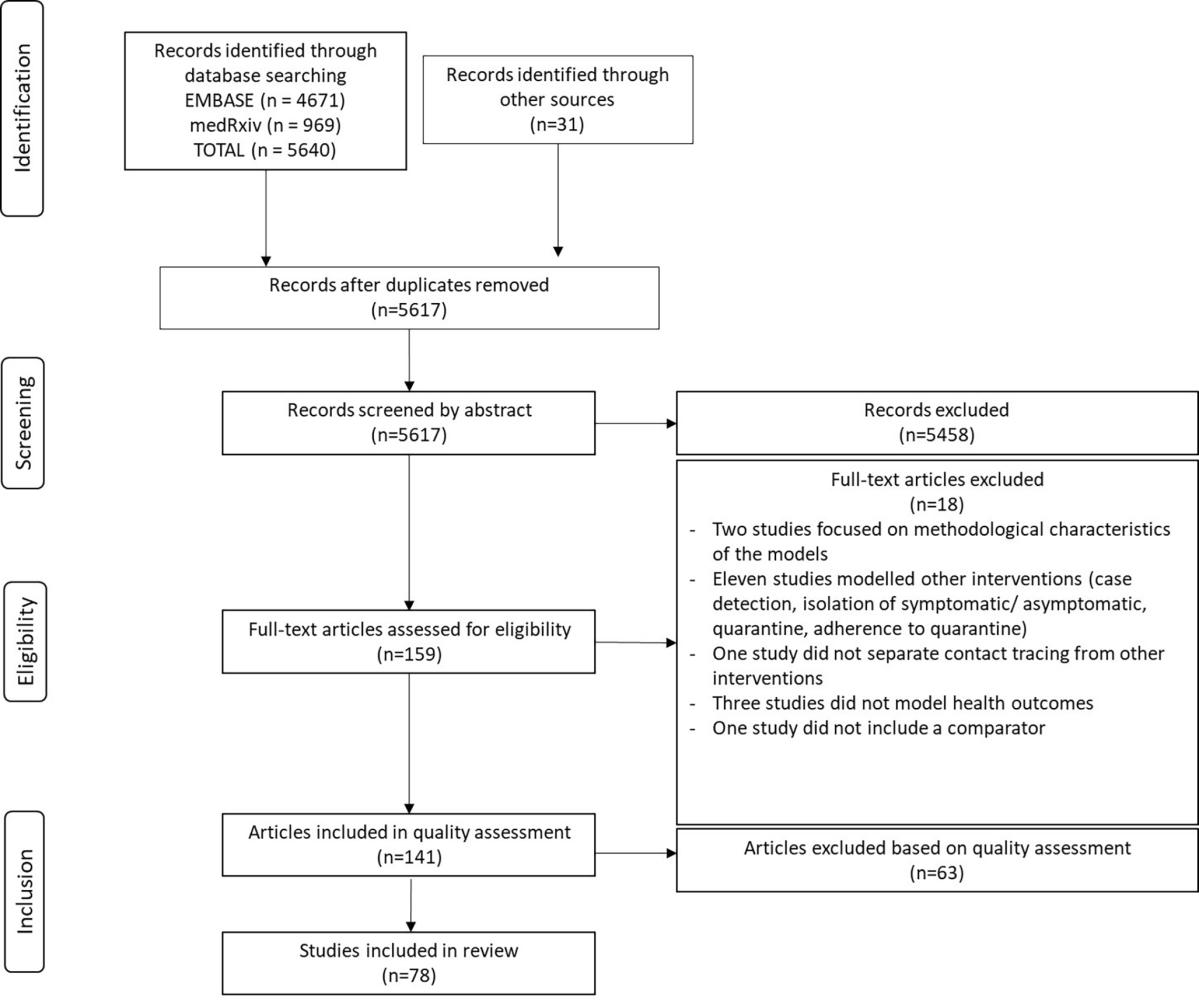
PRISMA flowchart

The initial search identified 5,617 records after removing duplicates across the databases. These records were screened and filtered based on whether any of the inclusion/ exclusion criteria were met based on the abstract. If unclear, the full-text was retrieved. Overall, 159 full-text records were assessed for eligibility. Of these, 141 met the inclusion criteria and were included in the quality assessment and 18 did not (See Fig. 1 for more details). 63 studies were excluded from the review based on the results from the quality assessment. 78 studies were included in the review, 67 of them published in peer-reviewed journals and 11 preprints. The full quality assessment of the 141 studies is available in Annex 2.2 of the supplementary information file.

### Methodological characteristics of the studies included in the analysis

#### Study type, timeframe and geographical scope

##### Study type

Out of the 78 studies included in the review, 12 studies were empirical (all of them observational) and 66 were mathematical modelling studies. Out of the 12 empirical studies, ten were ecological, of which nine were published [22–30] and one was a preprint [31]. One was a published retrospective cohort study [32] and another one was a published pre-post study of two COVID-19 patient cohorts [33]. Annex 3.1 in the supplementary information file provides an overview of each of these studies.

Out of the 66 mathematical modelling studies, 38 used agent-based models (ABM), i.e. models simulating COVID-19 infection and disease progression between groups of interacting individuals, 19 used stochastic branching process models (SBP) simulating COVID-19 outbreaks by tracking the sequential process of disease progression from an initial case or groups of cases and 9 used other varied disease modelling approaches (Other). Out of the 38 ABM-based studies, 32 were published [7, 12, 34–63] and six were preprints [64–69]. Out of the 19 SBP-based studies, 17 were published [5, 8–11, 70–81] and two were preprints [82, 83]. Of 9 studies using varied modelling approaches, seven were published [84–90] and two were preprints [91, 92]. Annex 3.2 in the supplementary information file provides an overview of the mathematical modelling studies.

##### Timeframe

In terms of timeframe, out of the 12 empirical studies, nine published studies [22–26, 28, 30, 32, 33] and one preprint [31] were based on data from the first wave of the COVID-19 pandemic. Only Pozo-Martin et al. [27] and Wibbens et al. [29] included data from further pandemic waves, the former until December 2020 and the latter until November 2020.

For the ABM-based mathematical modelling studies, based on the period of the epidemic modelled we identified two broad groups of studies: (1) Studies modelling the COVID-19 epidemic in a context other than the 2020 lockdown reopening, either in the general population [36–41, 46, 47, 54, 55, 57, 61, 64, 66–68], in a population of workers [44] or in a hospital [53] , and (2) studies modelling the COVID-19 epidemic in the context of the 2020 lockdown reopening, either in the general population [7, 12, 34, 35, 39, 45, 48, 49, 51, 52, 56, 58–60, 62, 65, 69] or in educational institutions [42, 43, 50, 63]. Within the first group, all studies modelled outbreaks over a variable time span (from 60 days [40] to 600 days [68]) from the first COVID-19 cases except three [38, 46, 54], which modelled the conditions of an ongoing epidemic, such as acquired immunity or vaccination. Within the second group of studies, all reproduced the conditions of specific 2020 lockdown and reopening scenarios in the modelling parameters except the studies set in educational institutions, which modelled outbreaks in the event of initiating at least some in-person teaching. Using the same grouping for the SBP-based studies: all the SBP-based studies modelled the epidemic in a context other than 2020 lockdown reopening from the first cases except Brook et al. [71], Fyles et al. [73] and Huamani et al. [75], who modelled 2020 lockdown reopening conditions. With respect to the varied modelling studies, all of them modelled the epidemic from its start in a context other than 2020 lockdown reopening with the exception of Moran et al. [91], who simulated events for an ongoing epidemic from June 2020 onwards.

##### Geographical scope

The geographical scope of the studies is varied. Six empirical studies assessed contact tracing in a wide range of geographical areas - Haug et al. [22] in 79 territories and 56 countries worldwide, Hong et al. [23] in 108 countries, Leffler et al. [25] in 200 countries around the world, Liu et al. [26] in 130 countries around the world, Papadopoulos et al. [31] in 137 countries, Pozo-Martin et al. [27] in the 37 OECD member states, while the remaining six focused in specific geographical contexts around the world. The ABMs simulated epidemics in either specific communities - e.g. in the University of Illinois [50], in Masiphumele township in Cape Town (South Africa) [66], in towns or cities, such as Boston MA (USA) [35] or Seattle WA (USA) [45], in regions - for example, Victoria (Australia) [56], or in an entire country, e.g. Luxembourg [60] or Belgium [62]. The SBP-based studies typically simulated local outbreaks in either generic unspecified contexts or in specific contexts, such as a student community at UC Berkeley at the start of a semester [71]. Studies not belonging to either category modelled outbreaks either in unspecified geographical areas [84], in cities, such as San Francisco CA (USA) [92] or in a country, e.g. the United Kingdom [86, 87].

#### Statistical and modelling approaches and parameters

##### Empirical studies

Annex 4.1 in the supplementary information file presents an overview of the type of study design, the modelling approach / statistical analysis, sample size and sources of data for the empirical studies.

The ecological studies used a wide range of statistical methods to assess the impact of contact tracing (among other NPIs) on the relevant health outcomes. Among the nine longitudinal ecological studies, Haug et al. [22] used four different approaches to separately estimate and then harmonize the impact of a vast number of NPIs on the reproduction number R: case-control analysis, step-function lasso regression, RF regression and transformer modelling. Pozo-Martin et al. [27] used both maximum likelihood and Bayesian estimation to estimate the impact of 13 NPIs including contact tracing on the weekly growth rate in cumulative COVID-19 cases. Kendall et al. [24] and Wibbens et al. [29] also used longitudinal Bayesian estimation/ modelling techniques to estimate, respectively, the impact of adding digital to manual contact tracing and the impact of eleven NPIs (including contact tracing) on the growth rate in cases. Wymant et al. [30] used both matched neighbour regression and modelling. The cross-sectional study [23] used multiple linear regression. The most common sources of data for these studies were a range of COVID-19 policy trackers – in particular, the Oxford COVID-19 Government Policy Tracker. The retrospective cohort study [32] used surveillance data and hypothesis tests to estimate, *inter alia*, the reduction in the number of secondary cases per diagnosed individual under contact tracing compared to symptomatic surveillance. The pre-post study [33] also used surveillance data to estimate the reduction in R associated with tracing and testing contacts of COVID-19 case clusters / symptomatic individuals compared to those of symptomatic individuals.

##### Simulation studies

The three categories of simulation models assessed in this review – ABM, SBP and Other models differ in fundamental aspects. ABMs simulate groups of interacting individuals, ranging from communities to entire populations. Each individual (i.e. each agent) is assigned particular characteristics which may affect the probability of infecting other individuals, becoming ill, recovering or dying. In contrast, SBPs simulate outbreaks starting with an index case or a small group of cases and track the sequential process of disease transmission. Models categorised as Other are neither ABMs nor SBPs but may share common characteristics with both. Annex 4.2 in the supplementary information file presents the main methodological characteristics of the mathematical modelling studies, including the representation of social interactions (specifically, the types of network layers and contact structure modelled for the interactions between individuals, with data sources), the representation of infection and disease (specifically, whether the models distinguish between symptomatic and asymptomatic carriers and/or levels of severity in COVID-19 symptoms) and the main model parameters and their sources.

##### ABMs

The ABMs from the 38 studies assessed in this review can be characterised into two broad types: multi-layer and single-layer ABMs. Multi-layer ABMs simulate different social layers (e.g. households, schools, workplaces) with different contact structures. In this sense, they are more realistic than single-layer ABMs. Within the multi-layer ABMs, the COVID-19 Agent-Based Simulator (COVASIM) is the most used - see [45, 46, 52, 56, 58]. COVASIM is an open-source ABM [93] which includes demographic data on age-structure and population size for specific countries, four different social layers (households, schools, workplaces, leisure) and a comprehensive description of health states, including asymptomatic/ presymptomatic/ mild/ severe/ critical/ dead. COVASIM incorporates different types of transmission networks, such as random networks and realistic networks via its integration with Synthpops, an open-source data-driven model that allows to generate synthetic contact networks based on evidence-based age-contact patterns for different environments such as schools and households [93]. Interestingly, many of the remaining ABM studies not using COVASIM also incorporate realistic network structures – for example, Aleta et al. [35] or Gressman et al. [42]. Studies that include less realistic age-contact patterns include those which model the age-contact structure using only the average number of contacts per age-group, e.g. Abueg et al. [34]. In addition, less realistic ABMs include those which are single-layer network ABMs- see for example [7, 38, 40, 41, 50, 55, 63], Reich et al. 1 [67], Reich 2 [68] and Tuomisto et al. [69]. Most ABM-based studies distinguish between symptomatic and asymptomatic transmission of COVID-19; exceptions include Bhattacharyya et al. [37], Wallentin et al. [7], Goldenbogen et al. [65], Low et al. [66], and Reich et al. 1 [67]. Some studies also distinguish between levels of severity of COVID-19 infected.

##### SBPs

As with the ABMs, the SBP used in the 19 studies assessed in this review can be separated into multi-layer and single-layer SBPs. Plank et al. [81] uses a multi-layer SBP (home, school, work and leisure). Seven studies [5, 10, 11, 70, 72, 75, 79] use adaptations of the SBP by Hellewell et al. [74]. This model is a SBP which simulates outbreaks with the following characteristics [74]: the number of potential secondary cases arising from an index case is distributed as a negative binomial distribution with mean equal to the reproduction number R; each new infection is assigned an incubation time (time between virus exposure and symptoms) for which a probability distribution is also assumed; once the individual is symptomatic he/she is isolated at a time drawn from a delay distribution; for each potential secondary case, depending on the study, a generation time (time between the infection of a primary case and one of its secondary cases) or a serial interval (time between the onset of symptoms in a transmission pair) is drawn from a distribution. Each contact is then traced with a probability p. Similar to the ABM studies, most SBP distinguish between symptomatic and asymptomatic transmission of COVID-19, except for Endo et al. [9] and Huang et al. [83]. In contrast with the ABMs, most SBPs do not distinguish between different levels of infection severity, except for Allali et al. [82].

##### Other models

Nine models belonging to this category were assessed in this review. For example, Kucharski et al. [87] uses a model starting with a number of infected and simulating contacts via an age-based contact distribution. Cencetti et al. [84] uses recursive equations where time is modelled in discrete steps. Grassly et al. [86] uses a time-dependent infectiousness function which distinguishes between symptomatic and asymptomatic infected individuals. Worden et al. [92] uses Monte Carlo methods to simulate outbreaks. Two of the models used in this group of studies are multi-layer [84, 87]. All of these models but one - see Worden et al. [92], separate symptomatic and asymptomatic transmission of COVID-19.

##### Parameters used in simulation studies and their sources

Parameters describing COVID-19 infectiousness commonly used across models include (1) the basic reproduction number R_0_ (the average number of new cases generated by an index case); (2) the incubation time; (3) the latency period (time between exposure and infectiousness), used in ABMs; (4) the generation time (the time between the infection of a primary case and the infection of a secondary case) and serial interval (the time between onset of symptoms in a transmission pair), used in SBPs and Other models; and (5) the proportion of asymptomatic cases:

1. Different values of R_0_ are used across models, typically when setting scenarios of higher or lower virus transmission. For example, Huamani et al. [75] uses R_0_ values of 2.7 and 3.5 for pre-lockdown and 1.5, 2.0 and 2.7 post-lockdown, based on estimates by Liu et al. [94] and Chen et al. [95]. Liu et al. [94] is referenced as a source of R_0_ in several studies, including Wallentin et al. [7], Huamani et al. [75], James et al. [76] and Pollmann et al. [54] - this paper reviews the first estimates of R_0_ in China, concluding that the mean (median) value for this parameter is 3.28 (2.79).
2. The incubation time is set to relatively similar values across most studies. COVASIM-based studies estimate a mean value for this parameter of 5.6 days based on a statistical analysis of cases by Linton et al. [96]. Several other ABM studies – e.g. Gressman et al. [42], Pham et al. [53], and Tuomisto et al. [69] assume a Gamma distribution for the incubation time between 5 and 6 days. In most SBP studies based on the model by Hellewell et al. [74], the incubation time is assumed to follow a Lognormal [5, 10] or Weibull [11, 72, 74] distribution with mean (standard deviation) in the range 5.5–5.8 (2.3–2.6) days. The most used reference for incubation time is Lauer et al. [97]. Lauer et al. [97] is indeed cited as a source for the incubation time by a number of ABM studies including Abueg et al. [34], Bicher et al. [12], Colomer et al. [38], Fiore et al. [40], and Pollmann et al. [54]; a number of SBP studies such as Bradshaw et al. 1 [10], James et al. [76], Bradshaw et al. 2 [70], Plank et al. [81], and by two studies included in the Other model types category [85, 86]. Lauer et al. [97] estimate the duration of the COVID-19 incubation period by analysing the cumulative number of confirmed COVID-19 cases reported between January 4 and February 24, 2020 in 50 regions and countries. Other widely used references for the incubation time include Backer et al. [98] and Li et al. [99].
3. The latency period is also set to relatively similar values across most studies. COVASIM-based studies use a lognormal distribution with mean (standard deviation) 4.5 (1.5) days based on Lauer et al. [97] and Nishiura et al. [100]. Aleta et al. [35] uses values ranging between 3 and 5 days, based on estimations by Backer et al. [98]. Bicher et al. [12] and Tatapudi et al. [59] use a latency period of 3 days. Ng et al. [51] use a PERT distribution with mean 3.68 days to characterise this parameter. Lauer et al. [97] is again used by multiple studies as a source for this parameter.
4. The serial interval and the generation time are also set to relatively similar values across most studies. Hellewell-based studies (all of them SBPs) mainly assume a Skew-Normal distribution for the serial interval with mean the incubation time and standard deviation equal to 2, e.g. Hellewell et al. [74], Bradshaw et al. 1 [10], Filonets et al. [72], Firth et al. [11] and Bradshaw et al. 2 [70]. Other SBP studies mainly assume a Weibull distribution for the generation time with mean 5.00-5.05 and standard deviation 1.92–1.94, based on Ferretti et al. [85].
5. The proportion of asymptomatic cases is modelled differently. COVASIM-based studies assume a different proportion of asymptomatic infected individuals by age groups, which are based on estimates by Ferguson et al. [101] and Verity et al. [102]. Other studies such as Abueg et al. [34], Moreno Lopez et al. [49] and Thompson et al. [60] also model a varying proportion of asymptomatic infected for different age groups. This approach adds realism to the representation of asymptomatic infected individuals in models. Aleta et al. [35] and Ng et al. [51] use equal values across all age groups for this parameter respectively of 25% and 38%. Nishiura et al. [100] is often cited as a source for the proportion of asymptomatic cases [35, 75, 77]. Nishiura et al. [100] analyse PCR results from 565 Japanese citizens evacuated from Wuhan and calculate the proportion of asymptomatic infected using Bayes’ theorem. Other sources commonly used for this parameter are Lavezzo et al. [103] and Mizumoto et al. [104].

To summarise, a host of statistical/ modelling approaches have been used to estimate the comparative effectiveness of contact tracing interventions. Among the empirical studies, longitudinal ecological designs evaluating the impact of contact tracing along with that of other NPIs on different health outcomes are predominant. The simulation studies differ substantially in their realistic representation of populations or outbreaks. Finally, there is relative consistency in the parameters commonly used across simulation models, whereby these parameters are extracted from adequate sources.

### Results of the studies

#### Empirical studies

Table 2 presents the results of the empirical studies, in descending order based (where applicable) on their quality score.

**Table 2:** Results of empirical studies

Authors; Setting; Longitudinal/ cross-sectional	Intervention(s)/ comparator(s)	Results relevant to contact tracing with effect sizes in brackets	Quality score/ Comment (studies with score < 16)
Wymant et al. [30] England and Wales Cross-sectional study	Contact tracing using National Health Service (NHS) COVID-19 app. Comparator: no app	Cases/ deaths averted in the period between end of September and end of December 2020: - Statistical estimation (95% CI): 594,000 (317,000-914,000) / 8,700 (4,700 − 13,500) and - Modelling (sensitivity analysis exploring 2.5-97.5% of variability in modelling estimates): 284,000 (108,000-450,000)/ 4,200 (1,600-6,600) Approximately one case was averted for each case consenting to notification of their contacts. For every percentage point increase in app uptake, the number of cases could be reduced by 0.8% (sensitivity analysis exploring 2.5-97.5% of variability in modelling estimates: 0.37-1.10%) or 2.3% (95% CI: 1.5-3%) depending on the estimation procedure	17
Kendall et al. [24] Isle of Wight (UK) Longitudinal study	Manual contact tracing + contact tracing using automated app. Comparator: Manual contact tracing	Results are presented graphically. After the introduction of contact tracing in the Isle of Wight, between May 5 and June 29 there is a drop in R from 1.3 to 0.5; at the same point in time, R was lower than in the Upper Tier Local Health Authorities of the UK as a whole (p < 0.0001)	17
Pozo-Martin et al. [27] 37 OECD countries Longitudinal study	(1) School closing requirements; (2) Workplace closing requirements; (3) Public events cancelling requirements; (4) Restrictions on gatherings; (5) Public transport restrictions; (6) Stay-at-home requirements; (7) Restrictions on internal travel; (8) International travel controls; (9) Public health information campaigns; (10) Mask wearing requirements; (11) Testing policy; (12) Contact tracing policy. Interventions are compared with each other.	Impact of contact tracing is not significant (exact effect size not provided)	16
Haug et al. [22] 79 territories and 46 countries worldwide Longitudinal study	42,151 NPIs including contact tracing. Interventions are compared to each other	Impact of contact tracing is not significant (exact effect size not provided)	16
Liu et al. [26] 130 countries worldwide Longitudinal study	(1) Internal containment and closure (School and workplace closure, public event cancellation, limits on gathering sizes, public transport closure, stay-at-home requirement, internal movement restriction); (2) International travel restrictions; (3) Economic policies; (4) Health systems policies (Public information campaign, testing policy, contact tracing). Interventions are compared with each other	Weak evidence of an association between contact tracing and an increase in R at 10 days (exact effect size is not provided)	15 No inclusion of covariates
Vecino-Ortiz et al. [28] 32 departments and 5 districts in Colombia Longitudinal study	Contact tracing as implemented in different departments and districts	A 10% increase in the proportion of cases identified through contact tracing in the previous 3 to 8 weeks is associated with COVID-19 mortality reductions between 0.8% and 3.4%. Regression coefficients for 0.8% mortality reduction (SE), cases traced 3 weeks previously: -0.078 (0.034), p-value = 0.024; Regression coefficients for 3.5% mortality reduction (SE) cases traced 3/4/6/8 weeks prior: -0.203 (0.042), p-value < 0.001/ 0.012 (0.036), p-value = 0.737/ -0.062 (0.030), p-value = 0.04/ -0.080 (0.026), p-value = 0.002	15 Analytical methodology less flexible
Leffler et al. [25] 200 countries Longitudinal study	(1) School closing; (2) Workplace closing; (3) Cancelling of public events; (4) Restrictions on gatherings; (5) Public transport closure; (6) Stay-at-home requirements; (7) Internal movement restrictions; (8) International travel restrictions; (9) Income support; (10) Public information campaigns; (11) Testing policy; (12) Contact tracing policy; (13) Mask-wearing	Impact of contact tracing is not significant. Regression coefficient (95% CI): -0.176 (-0.357 to 0.006), p-value = 0.06	13 Number of data points low
Wibbens et al. [29] Growth rate in cases 40 territories: 17 countries and 23 US states Longitudinal study	(1) Closing of schools; (2) Closing of workplaces; (3) Public event cancelling; (4) Gathering bans; (5) Public transport closure; (6) Shelter-in-place orders and home confinement; (7) Restrictions on internal movement; (8) Restrictions on international travel; (9) Public information campaigns; (10) Testing access; (11) Contact tracing. Interventions are compared with each other.	Marginal effect of contact tracing on reducing weekly growth rates is weak (results presented graphically, exact effect size not provided)	13 No inclusion of covariates
Papadopoulos et al. [31] 137 countries worldwide Longitudinal study	(1) School closing; (2) Workplace closing; (3) Cancelling of public events; (4) Restriction on gatherings; (5) Closure of public transport; (6) Stay-at-home restrictions; (7) Domestic travel restrictions; (8) International travel restrictions; (9) Public information; (10) Testing framework; (11) Contact tracing. Interventions are compared with each other	No evidence of a consistent association between high intensity contact tracing and *decreased* health outcomes (cases and deaths). Regression coefficient (95% CI) for cases/ deaths: 0.167 (0.006–0.316), p-value = 0.041 / 0.09 (-0.096-0.276), p-value = 0.296. There was no evidence of an association between timing of contact tracing and health outcomes. Regression coefficient (95% CI) for cases/ deaths: -0.019 (-0.228-0.153), p-value = 0.820 / -0.12 (-0.179-0.144), p-value = 0.893	11 Number of data points low, analytical methodology less flexible
Hong et al. [23] 108 countries Cross-sectional study	Assembly Restrictions (A): School closures (A1), Workplace closures (A2), Cancel public events (A3), Gathering size restriction (A4); Movement Restrictions (M): Close public transport (M1), Stay at home requirement (M2), Internal movement restrictions (M3), International travel restrictions (M4); Privacy Restriction (P): Contact tracing (P1). Interventions compared with each other	School closing in combination with full contact tracing (i.e. contact tracing done for all cases, used as an interaction term) has an impact on the decrease rate of increase in cumulative confirmed cases, leading to lower COVID-19 growth rate. Regression coefficients (SE) school closures / contact tracing as an interaction term: -2.070 (0.833), p-value < 0.05 / 0.227 (1.033)	11 Number of data points low, analytical methodology less flexible
Malheiro et al. [32] Eastern Porto (Portugal) Longitudinal study	(1) Contact tracing and quarantine. Comparator: no contact tracing and quarantine	Impact tracing and quarantine did not have an impact. Secondary attack rate in intervention group (95% CI) = 12·1% (7·1–18·9]; Secondary attack rate in control group (95% CI): 9·2% (7·8–10·8), p-value = 0.13	Acceptable Risk of performance bias
Park et al. [33] Seoul (South Korea) Longitudinal study	(1) Tracing the contacts of all COVID-19 case clusters and symptomatic individuals, testing them and placing all those testing positive in quarantine. Comparator: Testing only symptomatic, tracing and testing their contacts and quarantining all those testing positive	With tracing and testing the contacts of COVID-19 case clusters / symptomatic individuals and placing all those testing positive in quarantine the effective reproduction number was reduced from 1.3 to 0.6 (effect size not provided)	Acceptable Risk of selection bias

We separated the ecological studies into three categories based on their risk of bias rating: lower quality studies (risk of bias rating 11 or 12), intermediate quality studies (risk of bias rating 13 to 15), and higher quality studies (risk of bias rating 16 or 17). In the highest quality ecological study, Wymant et al. [30] found that use of the NHS COVID-19 app averted a large number of cases (594,000 and 284,000, depending on the method of estimation) between the end of September and the end of December 2020. They estimated that for each case consenting to notification of their contacts approximately one case could be averted and that for every percentage increase in app adoption cases could be reduced, depending on the method of estimation, by 2.3% or 0.8%. In the second highest quality ecological study, Kendall et al. [24] found that, after the implementation of a test, trace and isolate intervention including manual and digital contact tracing in the Isle of Wight, there was a consistent drop in the effective reproductive number from 1.3 to 0.5 [24]. Vecino-Ortiz et al. [28], in an ecological study of intermediate quality comparing the impact of contact tracing across 32 departments and five districts in Colombia, found that an increase in the proportion of cases identified through contact tracing of 10% was associated with a reduction in COVID-19 mortality of between 0.8% and 3.4%.

In a retrospective cohort study of acceptable quality (as defined by the SIGN risk of bias checklist, acceptable quality refers to neither high quality nor of unacceptably low quality), Malheiro et al. [32] compared (1) the number of secondary cases from index cases who were not subject to contact tracing and quarantine before laboratory confirmation of COVID-19 status with (2) the number of secondary cases from close contacts of index cases who were traced and quarantined before laboratory confirmation of COVID-19 status. The authors found that contact tracing was not associated with a reduction in the number of secondary cases per contact. In a pre-post study of two cohorts of COVID-19 patients of acceptable quality (as defined by the SIGN checklist), Park et al. [33] found that prompt tracing of contacts of COVID-19 case clusters/ symptomatic individuals was associated with a reduction in R from 1.3 to 0.6.

In the ecological studies which explored the comparative effectiveness of contact tracing in the context of a broad set of other (mostly social distancing) NPIs, contact tracing showed a very small effect on reducing weekly COVID-19 growth rates in Wibbens et al. [29]. Hong et al. [23] found that school closing and high-intensity contact tracing can, implemented together, have an effect on reducing the COVID-19 growth rate. Papadopoulos et al. [31], in a multivariate analysis comparing several NPIs, found no association between early adoption of contact tracing and reduced morbidity/ mortality. In a univariate analysis (i.e. not including the effect of other NPIs), the authors found that contact tracing was associated with an *increase* in the number of COVID-19 cases but neither with a decrease in the number of cases nor with a decrease in the number of deaths [31]. The remaining studies exploring contact tracing along other NPIs found no impact of contact tracing on health outcomes either in the first wave of the epidemic [22, 25, 26] or in both the first wave of the epidemic and in the period October-December 2020 [27].

#### Simulation studies

The simulation studies varied enormously, *inter alia*, in the geographical context, outcomes measured, point of the epidemic explored, and additional NPIs factored into the analysis. In addition, the majority of simulation studies reported results graphically and supported this graphical presentation with a descriptive narration regarding the specific aspects of the simulated contact tracing interventions which had a substantial impact on the epidemic. In this challenging context for evidence synthesis, we used the following approach to present the study results. First, we separated the studies into two types: (1) those that explicitly reported numerical changes in outcomes relevant to the contact tracing interventions, and (2) those that highlighted the specific contact tracing interventions modelled which could achieve COVID-19 epidemic control / suppression (R ≤ 1). Within both groups, we classified the studies into the two types described previously regarding the period of the epidemic modelled: (a) Studies modelling the epidemic in a context other than the 2020 lockdown reopening, and (b) Studies modelling the epidemic in the context of a 2020 lockdown reopening. In addition, for each study we made explicit whether conditions of social distancing / reductions in transmission were incorporated in the simulations of specific contact tracing interventions. In order to categorise the evidence for the studies explicitly reporting numerical changes in outcomes relevant to the contact tracing interventions analysed, we separated the contact tracing interventions reported into those that achieved high effectiveness (> 50% of reduction in the outcomes reported), intermediate effectiveness (between 10% and 50% reduction in the outcomes reported) and low effectiveness (< 10% reduction in outcomes reported). The outcomes reported include the effective reproduction number R, incidence-related outcomes (e.g. the attack rate, the number of susceptible individuals, infections, cases, hospitalisations and recovered individuals) and mortality. For a full description of the mathematical modelling study results, please see Annex 5 in the supplementary information file.

Table 3 below highlights the contact tracing interventions achieving high, intermediate, and low effectiveness for the studies modelling the epidemic in a context other than reopening a 2020 lockdown which explicitly reported numerical changes in outcomes. From Table 3, in studies modelling the epidemic in a context other than the 2020 lockdown reopening, the following contact tracing interventions were highly effective:

- In the context of manual primary contact tracing, high manual forward tracing coverage with medium term immunity or high isolation/ quarantine efficacy and/or physical distancing. In a context of physical distancing and mid-term immunity, tracing and testing 40% of contacts [91] resulted in a reduction of 99% in the number of deaths. With high isolation/ quarantine efficacy, tracing all contacts achieved reductions in R of 64% [87]. Wells et al. [61] found that with levels of quarantine efficacy of 47% tracing all infected individuals could reduce the epidemic size by 95%. Eilersen et al. [39] estimated that an approach of one-step tracing, identification and highly efficacious quarantining of social contacts of individuals testing positive could reduce the peak number of infected by 60%. Colomer et al. [38] found that, with social distancing and a population vaccination level of 19% in the summer of 2021, a lower contact tracing coverage of 40% would reduce deaths by 71%/77% depending on the level of social distancing.
- Hybrid manual and digital contact tracing with high app coverage and high isolation/ quarantine efficacy. Plank et al. [81] estimated that a fast and effective contact tracing strategy with high quarantine efficacy and digital contact tracing with 75% app adoption reduced R by 53%. Kucharski et al. [87] found that adding digital contact tracing with 53% app adoption to manual tracing of acquaintances achieved a reduction in R of 61%. Both authors found that the efficacy of hybrid contact tracing increased with physical distancing. Kucharski et al. [87] in addition found that digital contact tracing on its own had no advantage over manual contact tracing.
- Secondary contact tracing. Geffen et al. [64] found that with perfect isolation of infected and perfect tracing of first and secondary contacts the number of infections was reduced by 82%. Firth et al. [11] found that secondary manual contact tracing achieved a reduction in infections of 78%. Bhattacharya et al. [37] estimated that coupled with a moderate lockdown, secondary contact tracing may achieve a 99% reduction in recovered individuals.
- Immediate contact tracing from identification of index case (i.e. no delays in contact tracing). Quilty et al. [55] found that in a context with moderate/ high quarantine efficacy, a reduction in tracing delays from three to zero days could avert 58% of transmissions.
- Bidirectional contact tracing. Endo et al. [9] found that across a wide level of relevant infection- and policy-related parameters, bidirectional contact tracing could avert two or three times more cases than forward contact tracing alone.

**Table 3:** Specific contact tracing interventions achieving high (> 50% reduction in reported outcomes), intermediate (between 10% and 50% reduction in reported outcomes) and low effectiveness (< 10% reduction in reported outcomes): studies modelling the epidemic in a context other than the 2020 lockdown reopening

Specific intervention modelled	High effectiveness	Intermediate effectiveness	Low effectiveness
**Manual forward tracing of primary contacts**	- Isolation (cases + HH contacts) + tracing all contacts [87] *** R reduced by 64% - One-step tracing, identification and quarantining of social contacts of those testing + (with 10% of infected tested per day of illness) [39] *** peak number of infected reduced by 60% - Contact tracing with 100% trace and quarantine efficacy [44] *** Reduced peak in infectious prevalence by 75% - Contact tracing of symptomatic and variable tracing rate of asymptomatic (0-100%) [47] *** Infection density per 10,000 population reduced between 65-88% - Contact tracing of any infected individual with higher than 47% quarantine efficacy [61] *** epidemic size is reduced by 95% - Isolating cases, tracing and testing contacts with 40% efficacy [91] *** Deaths reduced by 99% - In the context of advanced epidemic with vaccination and with/without social distancing, tracing 40% of contacts [38] *** Deaths reduced by 71%/74%	- Manual tracing of HH (instant), School (0.5 days after case isolation), Work/ Causal contacts (within three days of case isolation) with moderate (50%) quarantine efficacy [81] *** R is reduced by 35% - Tracing and testing HCW with a two-day/ seven-day window and reduction in transmission due to PPE [53] *** Reduction in R of 32-37% - Isolation of infected (85%) + 10%/30% contact tracing coverage [64] *** Reduction of infections by 23%/41% - Tracing and testing 80% of HH/ Work/ School contacts with two-day test turnaround time [66] *** Reduction in infections 25.5% - Contact tracing without (with) mask-wearing with a shelter in place order [92] *** Reduction in reported cases 11% (30%)	- Tracing and testing 80% HH/ Work/ School contacts with eight-day test turnaround time [66] *** Infections reduced by 8%
**Digital or hybrid (manual + digital) contact tracing**	- Hybrid tracing of HH/ Work/ School/ Casual contacts within 3 days with moderate/ high quarantine efficacy and 75% app adoption [81] ***R reduced by 48%/53% - Manual tracing of acquaintances and digital contact tracing with 53% app adoption [87] *** R reduced by 61%	- Random testing with 10 tests per thousand per day and digital contact tracing (50% app adoption) [88] *** Infections reduced by 20%	
**Tracing of secondary contacts**	- Tracing primary contacts with variable (0.3–0.9) probability of success and tracing their secondary contacts [11] *** Infections reduced by 78% - Perfect isolation of infected + 100% contact tracing coverage of primary and secondary contacts [64] *** Infections reduced by 82% - Tracing secondary contacts with a mild lockdown [37] *** Recovered reduced by 99%		
**Delays to contact tracing**	- With 14-day isolation/ quarantine (57-67% adherence), reducing tracing delay from three to zero days [55] *** 58% of transmissions averted	- Testing 80% of symptomatic and contact tracing (80% coverage) with one-day delay to contact tracing [86] *** R reduced by 26%	- Testing 50% of symptomatic and contact tracing (50% coverage) with two-day delay to contact tracing [86] *** R reduced by 8%
**Backward or bidirectional contact tracing**	- Bidirectional contact tracing across a broad range of infectivity, tracing coverage and quarantine efficacy levels [9] *** Increased number of cases averted by 200-300%	- Hybrid bidirectional contact tracing with low/high uptake, a two-day tracing window and social distancing [10] *** R reduced by 21%/ 42% - Bidirectional contact tracing with 2-day window (70% coverage) or bidirectional contact tracing with 6-day window (50% coverage) [70] *** R reduced by 10% - Hybrid bidirectional contact tracing with Bluetooth technology (20% adoption) [36] *** Infections reduced by 10%	

* HH = households; HCW = health care workers

The following contact tracing interventions had intermediate effectiveness:

- In the context of manual forward contact tracing, different levels of contact tracing coverage coupled with either quick quarantine or high isolation and quarantine efficacy. Plank et al. [81] found that tracing of school/work/casual contacts with 50% quarantine efficiency achieved an R reduction of 35%. Low et al. [66] found that testing and isolating infected contacts with a two-day test turnaround time reduced the number of infections by 25.5%. Geffen et al. [64] found that with a strong isolation policy, levels of 10%/30% contact tracing could achieve a 23%/41% infection reduction.
- Digital contact tracing with intermediate levels of app adoption. Kuzdeuov et al. [88] estimated that a level of 50% app adoption could lead to a 20% reduction in infections in the context of mass random testing.
- Small (i.e. 1 day) contact tracing delays. Grassly et al. [86] found that in the context of very high levels of testing of symptomatic individuals and contact tracing, a one-day delay to contact tracing could induce a reduction in R of 26%.
- Longer bidirectional contact tracing windows. In the study by Bradshaw et al. 2 [70], bidirectional contact tracing with a six-day window and 50% coverage would reduce R by 10%; to obtain the same effect with a 2-day tracing window, a higher level of coverage of 70% would be required.

The following contact tracing interventions had low effectiveness:

- Longer delays to contact quarantine. Low et al. [66] found that a contact quarantine delay induced by an 8-day delay in the time from contacts testing to achieving test results could reduce infections by 8%.
- Longer (i.e. 2 days) delays to contact tracing. In the same study discussed previously, Grassly et al. [86] found that with high levels of symptomatic testing and contact tracing, a two-day contact tracing delay resulted in a reduction in R of 8%.

Table 4 below highlights the contact tracing interventions achieving high, intermediate, and low effectiveness for the studies modelling the epidemic in the context of 2020 lockdown reopening which explicitly reported numerical changes in outcomes. Based on the results from Table 4, the following contact tracing interventions were highly effective in studies modelling contact tracing in the context of 2020 lockdown reopening scenarios:

- In forward manual primary contact tracing, high forward tracing coverage levels coupled with high isolation and/ or quarantine efficacy and with social distancing after reopening. Ng et al. [51] and Bicher et al. [12] found in the context of strong isolation policies and social distancing after reopening, that 100% and 50% tracing coverage achieved a 99% reduction in the attack rate and a 62% reduction in infections respectively. Tatapudi et al. [59] found, with social distancing after reopening, a 66% reduction in the infection rate with a strong contact tracing policy identifying 50% of symptomatic and asymptomatic individuals. Willem et al. [62] estimated that, with social distancing, identifying 50% of symptomatic and tracing their contacts with high coverage (90% in households, 50% outside of households) reduced hospitalisations by 58%.
- Digital contact tracing alone or hybrid manual and digital contact tracing, both with high app adoption, and social distancing. Moreno Lopez [49] found that with high/ low social distancing after reopening, digital contact tracing with 60% app adoption achieved a reduction in peak incidence of 89%/66%. Abueg et al. [34] in the context of reopening with mask wearing and closed schools, found that digital tracing with 75% app adoption could reduce infections between 56% and 73% (low estimates) in three counties in the USA.
- Reopening educational institutions with high levels of contact tracing and with social distancing. Brook et al. [71] estimated a very large (x17) increase in the number of cases saved with a policy reaching 90% of contacts within one day of identifying the student index case. Zafarnejad et al. [63] estimated, in a context of surveillance testing, that shifting from no contact tracing to the maximum level could avert 70% of cases in reopening an educational institution.

**Table 4:** Specific contact tracing interventions achieving high (> 50% reduction in reported outcomes), intermediate (between 10% and 50% reduction in reported outcomes) and low effectiveness (< 10% reduction in reported outcomes): studies modelling the epidemic in the context of 2020 lockdown reopening

Specific intervention modelled	High effectiveness	Intermediate effectiveness	Low effectiveness
**Manual forward tracing of primary contacts**	- Enhanced (50%) case detection, isolation of HH and contact tracing (100% coverage) with social distancing [51] *** Attack rate reduced by 99% - Manual tracing of HH/Work contacts and 50% casual contacts with social distancing [12] *** Infections reduced by 62% - Contact tracing identifying 50% of symptomatic/ asymptomatic infected with social distancing [59] *** Infections reduced by 66% - Suppression strategy testing all symptomatic and progressive increase in contract tracing over time to 60% [69] *** Reduction in cases 90% - Tracing infected contacts with a 20%/60% coverage with high quarantine success and social distancing [48] *** Cases reduced by 84.6%/ 92.4% − 50% symptomatic traced (90% HH contacts, 50% nonHH contacts) with social distancing [62] *** Hospitalisations reduced by 58% - Testing symptomatic (30%/50%) + HHquarantine + tracing 60%/40% of non-household contacts [35] ***Hospitalisations reduced by 87%/86%	- Manual tracing of HH/Work contacts with strong social distancing [12] *** Infections reduced by 41%/ 35%	
**Digital or hybrid (manual + digital) contact tracing**	- Isolation (cases and HH contacts) and digital contact tracing (60% app adoption) with high/low social distancing [49] *** Reduction in peak incidence 89%/66% - Digital contact tracing with 75% app adoption in three counties with social distancing [34] *** Infections reduced by ranges of 56% and 73% / 73% and 79% / 67–81%.	- Isolation (cases and HH contacts) and digital contact tracing (20% app adoption) with lower/higher levels of social distancing [49] *** Infections reduced by 35%/ 45%	
**Reopening in educational institutions**	- In the context of reopening educational institutions, contact tracing with 100% trace and quarantine efficacy [43] *** proportion of students infected reduced by 68% - In the context of reopening educational institutions with social distancing, contact tracing (90% success) with a time to trace delay of 1 day [71] *** Cases saved per index case increased x17 - In the context of reopening educational institutions, surveillance testing and contact tracing with some social distancing [63] *** Switching from no contact tracing to maximum contact tracing, rate of infection reduced by 70%		- Reopening of educational institutions without social distancing. Random testing (15,000 tests per day) and increasing tracing between 80% and 90% contact tracing coverage [50] *** Infections reduced by 5.54% - Reopening of educational institutions with strong social distancing, random testing and contact tracing [42] *** Infections reduced by 8.5%

*HH = household

The following contact tracing interventions had intermediate effectiveness:

- Manual contact tracing of household or work contacts with social distancing. Bicher et al. [12] found that, in a context of strong social distancing after reopening, manual contact tracing of household/ work contacts reduced the number of infections by 41%/ 35%.
- Isolation of household contacts and digital contact tracing (low uptake) with social distancing. Moreno-Lopez et al. [49] found that in a context of isolation of cases and their household contacts digital contact tracing with 20% app adoption and lower/ higher social distancing reduced infections between 35% and 45%.

The following contact tracing interventions had low effectiveness:

- Reopening of educational institutions with random testing, contact tracing and social distancing and small changes in tracing coverage when it is already at a high level. In the study by Gressman et al. [42], contact tracing within a set of policies in educational institutions including random testing and social distancing reduced infections by 8.5%. However, the level of contact tracing coverage in the study was not clear. Mukherjee et al. [50], in a similar context, found that increasing from contact tracing coverage from 80% to 90% had a small (5.54%) impact on infection reduction.

Table 5 presents a set of specific contact tracing interventions which can achieve COVID-19 epidemic control / suppression (R ≤ 1) from the studies included in the review which did not explicitly report changes in numerical outcomes relevant to contact tracing but which highlighted these interventions. From Table 5, the results from the studies modelling the epidemic in contexts other than 2020 lockdown reopening echo the results that we have outlined above:

- For manual forward tracing, high levels of isolation, contact tracing and quarantine efficacy helped achieve epidemic control/ suppression [80], especially in the context of reduced transmission by, *inter alia*, social distancing interventions [40, 67, 74, 79].
- For digital or hybrid contact tracing, high level of smartphone use/ app adoption, particularly with social distancing [57, 72, 90] helped achieve epidemic control/ suppression. Cencetti et al. [84] found that with a two-day delay in contact tracing, high quarantine efficacy and strong social distancing, a level of app adoption of 40% was enough to control the epidemic.
- Shorter delays to contact tracing helped epidemic control: three days delay with highly successful quarantine and no social distancing [85]; zero days delay with 80% contact tracing and social distancing [8]; one day delay (i.e. one day to isolation of symptomatic) with physical distancing [82]; zero days without physical distancing and 100% coverage of household contacts [78]. Kretzschmar et al. 2 [78] found the following trade-off: for a tracing delay of zero days, contact tracing coverage of 40% or higher can achieve R < 1; however, if this tracing delay is increased to one day, tracing coverage needs to increase to 100% to achieve the same effect. In a similar context, Quilty et al. [55] found that reducing contact tracing delays may allow for shorter quarantine periods.

**Table 5:** Specific contact tracing interventions which can achieve epidemic control / extinction (R ≤ 1) from additional studies included in the review

	Studies not modelling contact tracing in reopening scenarios after 2020 lockdown	Studies modelling contact tracing in reopening scenarios after 2020 lockdown
**Manual forward tracing of primary contacts (including educational institutions)**	- Contact tracing (90%, time to trace 0.5 days) and 90% effective quarantine [80] *** Without other interventions, R < 1 - Isolation and contact tracing with 20% of undetected cases and strong social distancing [89] *** R < 1 - Contact tracing and isolation (90%) with low transmission (R = 1.5) but 40% of transmission asymptomatic [77] *** less than 20% probability of R < 1 - Contact tracing with high [medium-low] transmission efficiency + contact tracing (80-100%) [60-100%] + high [medium] contact testing capacity [40] *** R < 1 - Case isolation + contact tracing (90%) + mask wearing + physical distancing [79] *** R < 1 as long as with physical distancing R = 1.2 - Mass random testing (10%) + contact tracing (50%/100%) with/without social distancing [67] *** R < 1 - With pre-symptomatic transmission and low (R = 1.5)/ high (R = 2.5) infectivity and time to trace delay of 3.5 days, contact tracing (80%) [74] *** R < 1	- Testing of symptomatic individuals and asymptomatic contacts (90%) + contact tracing (90%) + social distancing [58] *** R < 1 - Full-time [part-time] school reopening with symptomatic testing 75% [65%] and contact tracing with 68% [40%] coverage + some social distancing [52] *** R < 1 - Case isolation and contact tracing after lifting of lockdown (R = 2.7) [75] *** R < 1 with early isolation and 100% of contacts traced
**Digital or hybrid (manual + digital) contact tracing**	- Isolation of cases with two-day delay + digital contact tracing (20% app adoption) + 50% quarantine efficacy + physical distancing (R = 1.2) [84] *** R < 1 - With high pre-symptomatic infectiousness, case isolation and contact tracing (60%) + high efficiency mask wearing (50%) + social distancing (R = 1.5) [72] *** R < 1 - Combination of lockdown and digital contact tracing (60% of population owns smartphones) [57] *** R < 1 - Digital contact tracing (40-60%) + time to trace 0 days + low (30%) probability of transmission [90] *** R = 1 - Digital contact tracing (90%) [80%] with time to trace = 0 days and 80% of symptomatic detected without/ with reduction in transmission [54] *** R < 1	- Digital contact tracing with 26% app adoption + physical distancing (20%) [7] *** R = 1
**Delays to contact tracing**	- Isolation of symptomatic + contact tracing with no social distancing [85] *** R < 1 if time to trace < 3 days + high success in quarantining contacts and isolating cases. R < 1 with time to trace = 0 and 70% success of quarantining contacts/ cases - Test symptomatic (80%) with a testing and tracing delay of 0 days and 80% contact tracing coverage [8] *** R < 1 - Contact tracing with 100% coverage of household contacts and no social distancing [78] *** time to trace must be at most one day for R < 1 - Delay to isolation of symptomatic (1 day) + highly successful contact tracing (3 asymptomatic detected per symptomatic) + physical distancing (R = 2) [82] *** R < 1 - Increasing contact tracing coverage (50-80%) compared to reducing time to trace from four to one days [68] *** Increasing contact tracing coverage is more effective than reducing time to trace	- With a test capacity of 2.7 thousand per day, contact tracing (70%) + time to trace = 2 days + mask wearing and school closures [45] *** R < 1
**Backward or bidirectional contact tracing**		- Contact tracing of symptomatic considering household structure and physical distancing (50%) / Backward digital contact tracing of individuals (50%) [73] *** R < 1 / Reduces growth rate if recall does not decay

The results from the studies modelling contact tracing in 2020 lockdown reopening scenarios similarly echo our previous results and add new information:

- For manual forward tracing, high levels of contact tracing and physical distancing helped attain epidemic control/ suppression [58]. In addition, full-time and part-time reopening of schools with high levels of testing and contact tracing and some social distancing can help epidemic control [52].
- Digital contact tracing with lower app adoption and physical distancing helped control/ suppress the epidemic. Wallentin et al. [7] found that in a context of 20% reduction in mobility at reopening, lower (26%) levels of digital contact tracing app adoption led to R = 1.
- With high contact tracing levels and lower transmission due to mask wearing and school closures, tracing delays of two days did not hinder control of the epidemic [45].
- Contact tracing coverage (50%) considering the structure of households with the addition of physical distancing helped R < 1 [73].

To summarise the results of the review for the modelling studies across levels of effectiveness:

1. Manual contact tracing with high tracing coverage is a highly effective intervention if accompanied by medium term immunity or high isolation/ quarantine efficacy and/or physical distancing. Excluding casual contacts from contact tracing may reduce the effectiveness of manual contact tracing. Manual contact tracing with longer delays to contact quarantine were found to have low effectiveness, which highlights the importance of high quarantine efficacy in the context of this intervention.
2. Hybrid contact tracing with high app adoption is a highly effective intervention if accompanied by high isolation and quarantine efficacy and social distancing. Moderate levels of app adoption reduce the effectiveness of this intervention.
3. Secondary contact tracing is a highly effective intervention.
4. Reducing delays to contact tracing (from three to zero) is increasingly effective, and immediate contact tracing is highly effective. Some 2020 lockdown reopening studies found that delays in tracing up to three days can be effective, particularly with social distancing. Other studies found that increases in tracing delays of only one day (from zero to one) require a very large increase in contact tracing coverage to achieve a similar effect, and that reducing tracing delays may allow for shorter quarantine periods.
5. Bidirectional contact tracing is highly effective. Longer (e.g. 6 days) tracing windows have been found to have intermediate effectiveness.
6. Contact tracing with high coverage in reopening educational institutions is highly effective. One study found that contact tracing in an educational institution had low effectiveness, but the level of tracing coverage was not clear. Small changes in tracing coverage when coverage is high in educational institutions have been shown to have low effectiveness.

## Discussion

*Study quality*.

The quality of the empirical studies was variable. Studies using large sample sizes and advanced statistical methods [24, 30] or using large databases and multiple, more sophisticated methods of analysis [22, 27] coexisted with studies with relatively small sample sizes and less sophisticated/ flexible statistical methods, e.g. [25, 31]. Most studies (ten out of twelve), however, were of intermediate or high quality. Specifically, this was the case for four out of the five studies with a statistically significant positive effect on reducing health outcomes [24, 28–30], hence highlighting the validity of the results reported in individual studies. For the mathematical modelling studies, there was less variability in quality than for the empirical studies. In addition, a full one half of the studies (33/66) reached a score of eight or nine (with the maximum possible being nine) and four fifths (57/66) achieved a score of seven, eight or nine, yielding more confidence in their results. Quality differences across models were for the most part due to differences in the representation of more realistic social mixing between individuals in the models. Specifically, as mentioned previously, agent-based models often (two-thirds of the time) implemented multi-layer networks. Such networks are based on actual interactions of individuals across different networks, such as at school, at work, or in the community and most realistically represent the interactions between individuals that can lead to disease spreading.

*Empirical studies*.

From the 12 empirical studies analysed in this review, two higher quality studies [24, 30], two intermediate quality studies [28, 29], one acceptable quality study [33] and one lower quality study [23] found an effect of contact tracing on controlling the COVID-19 pandemic, while six [22, 25–27, 31, 32] did not.

Implementing digital contact tracing in addition to manual contact tracing was identified in our review as an effective intervention in two high quality observational studies [24, 30]. Wymant et al. [30] suggest that the positive effect of the NHS COVID-19 app on health outcomes is due to a higher tracing speed and a higher coverage of contacts compared to manual contact tracing (the app detected 4.2 contacts per index case compared with 1.8 with manual contact tracing). Kendall et al. [24] suggest that among the reasons for the success of the implementation of digital contact tracing along with manual contact tracing in the Isle of Wight were the large advertising campaign, community discussions and national publicity that followed the launch of the initiative. While Vecino-Ortiz et al. [28] showed that increased levels of contact tracing had a significant impact on mortality in Colombia and Park et al. [33] found that tracing contacts associated with COVID-19 case clusters reduced the reproduction number R to levels compatible with epidemic control (albeit without providing an effect size) in Seoul (South Korea), Malheiro et al. [32] did not find that contact tracing and quarantine was more effective than symptomatic surveillance in Porto (Portugal). This last finding may be due to two explanations according to the authors. First, citing Nussbaumer-Streit et al. [20], they argue that considering the large reproduction number and the pre-symptomatic transmission of COVID-19, quarantine of contacts alone seems to be insufficient to contain the epidemic [32]. Second, they state that, in their cohort study, most high-risk contacts were household contacts and, in many cases, housing conditions could not guarantee that contacts could be truly isolated, and hence the chain of transmission was not immediately stopped [32].

Among the ecological studies exploring the joint implementation of contact tracing with other NPIs, Wibbens et al. [29] found that contact tracing had a very small marginal effect on reducing weekly COVID-19 growth rates across 40 jurisdictions: the authors suggest that this very small effect could be due to the lifting of policies, other than those reported in the database used in their study, at the same time as contact tracing was being implemented [29]. While Hong et al. [23], in a lower quality study, found that school closing was effective at reducing the pandemic growth rate only if implemented concurrently with high intensity contact tracing, the authors did not report an explanation for this effect. No other ecological studies exploring the impact of contact tracing in the context of other NPIs (including strict social distancing measures such as stay-at-home orders) found that contact tracing was a comparatively effective intervention for controlling the COVID-19 epidemic. Haug et al. [22] comment that this could be partially explained by two factors. First of all, their analysis was undertaken in April and May 2020, when contact tracing structures were overwhelmed in most countries rendering this policy ineffective [22]. Second, in countries where contacts were traced and tested, this policy would increase the reproduction number in the short term, as more cases will be found [22]. Liu et al. [26] share this last argument to explain the lack of impact of contact tracing in their study. They add that information bias in the database where they sourced their NPI data could also play a role. Pozo-Martin et al. [27], in their study of NPI impact in OECD member states discuss that the lack of effect of contact tracing shown in the early phase of the epidemic may be explained at least in part by the fact that for the period of study, most OECD countries implemented limited contact tracing (i.e. they did not trace the contacts of all confirmed cases). It is well known that ecological studies have limitations, for example being exposed to omitted variable bias. A further problem of assessing the effectiveness of contact tracing in the context of other NPIs is that it is statistically challenging because NPIs are typically implemented simultaneously- some statistical methods may overestimate the effects of an NPI due to insufficient adjustment for confounding from other measures, and other methods may underestimate the effect of an NPI by assigning its impact to a highly correlated NPI [22]. For this reason, the use of more than one statistical method to explore the effectiveness of joint NPI implementation is good practice (and in fact is included in the study quality rating tool used in our review). Two of the higher quality studies exploring the comparative effectiveness of contact tracing in relation to other NPIs [22, 27] used more than one statistical method to control for this potential problem and found results were consistent across methods.

*Mathematical modelling studies*.

Based on the results from the mathematical modelling studies, high contact tracing coverage is an important mitigation intervention, particularly in contexts of high COVID-19 transmission. This is because individuals become infectious days before the onset of symptoms and it is estimated that 35% of COVID-19 transmission is asymptomatic [105]. Manual contact tracing involves carrying out interviews with identified cases, contacting their contacts (usually by phone) and informing them about their likely exposure to the pathogen. It is labour intensive and time-consuming. In contexts of high COVID-19 transmission, high coverage of manual contact tracing may be difficult to achieve given health system resource constraints. In contrast, in contexts of low COVID-19 transmission, the probability of contact tracing achieving epidemic control increases [74]. Some have suggested that in contexts of low COVID-19 transmission, contact tracing is the key intervention in COVID-19 outbreak management and control [106].

There are a number of interventions that can directly help reduce COVID-19 transmission and hence increase the effectiveness of manual contact tracing. Three of these are robust case detection, high isolation/ quarantine efficacy, and non-pharmaceutical interventions increasing social distancing. As we showed in the review, a number of modelling studies found that manual contact tracing was highly effective in the context of such interventions. These interventions can be of particular relevance in reopening scenarios such as after the 2020 lockdowns. This is of course because (at a huge social cost) lockdowns achieve the interruption of SARS-CoV-2 transmission [35] and contact tracing then becomes feasible. Undeniably, the epidemic resurged during the second half of 2020 with the relaxation of NPIs. Modelling studies have been proposed which combine robust contact tracing with social distancing to mitigate the effect of NPI relaxation [59].

Digital contact tracing is a potential improvement over manual contact tracing. Once an index case is confirmed, the digital tracing app can immediately and automatically detect risky contacts of the index case, inform these contacts of their status and request that they quarantine. In a context of high transmission, it may perform the contact tracing task more efficiently than the staff involved in manual contact tracing. In addition, it does not rely on an index case’s recall of her/ his recent contact history. We found in our review that hybrid manual and digital contact tracing with high app adoption is highly effective with high isolation/ quarantine efficiency and with social distancing. These interventions reduce transmission and the number of contacts who are not known and who may be difficult to trace even with a tracing app. However, achieving high app adoption is not a given. In fact, the uptake of these apps in many countries has been slow [107]. A survey in Germany of 3,276 adults exploring the potential barriers for the adoption the official COVID-19 contact tracing app [108] found that potential spreaders (those with frequent contacts) had a high ability (91%) to adopt the app but a low willingness (31%) to adopt it correctly. For vulnerable groups the main barrier (62%) was access to the app [108]. The authors predicted an adoption rate of 34.7%, below the estimated 56% that epidemiological models predicted was required to contain the epidemic [108]. Panchal et al. [109], in a UK survey assessing the usability and functionality of the NHS COVID-19 contact tracing app, found via a readability analysis that about 40% of the UK population may not understand the information contained in the text displayed in the app, likely affecting its uptake.

Tracing secondary contacts (i.e. contacts of contacts) was found to be a very effective intervention. In effect, secondary contact tracing approach to contact tracing which essentially acts as a “local lockdown” [11]. In their model simulations, Firth et al. [11] found that secondary contact tracing may result at a given point in half of the population being quarantined. The authors suggest that combining contact tracing with other interventions (e.g. social distancing) may result in controlling the epidemic while reducing the number of quarantined contacts [11].

Bidirectional contact tracing can be highly effective. This is because it allows to identify the upstream source of a chain of transmission, and hence many more potentially exposed individuals. In addition, bidirectional contact tracing is quite effective when there is wide variability in the number of onwards transmissions across individuals, as is the case in COVID-19 [9]. Although bidirectional contact tracing has been used to successfully identify clusters of COVID-19 transmission in the community, e.g. in Singapore [10, 110], it is not common. For manual bidirectional contact tracing, extending the tracing window prior to symptom onset (for example, from two to six days) was found to be effective as contacts between infectors and infectees often occur several days before symptoms begin [10]. However, extending the tracing window requires contact tracers to trace many more contacts per index case, at an increasing cost, including in terms of individuals quarantined [10]. Bradshaw et al. 1 [10] propose to limit these costs via efficient prioritisation of forward and backward contact tracing. For example, since individuals identified through backward tracing are unlikely to still be highly infectious, the need for quarantine without a positive test is reduced and an efficient contact tracing programme may prioritise backward tracing (and testing) of contacts from three to six days before the start of symptoms and then initiate forward tracing from the identified cases [10]. This is a similar approach to that of Japan’s contact tracing programme [10]. One important issue that may affect manual bidirectional contact tracing effectiveness is loss of recall. Fyles et al. [73] found that a reduction in the probability of recalling a contact of 10% per day may eliminate all the gains due to backward contact tracing. Hybrid manual and digital bidirectional contact tracing has been identified as a highly performing alternative to manual bidirectional contact tracing. Bradshaw et al. 1 [10] found that with a short tracing window of two days, supplementing manual bidirectional contact tracing with digital contact tracing improved contact tracing performance. Digital contact tracing has the added advantage over manual contact tracing of being fast and scalable, although it has the disadvantage that it is subject to network fragmentation due to insufficient adoption of the contact tracing app [10]. Other approaches to hybrid bidirectional contact tracing using digital applications include the use of Bluetooth beacons placed in places where individuals congregate, which have shown to be effective [36]. The bidirectional interoperability of these systems with manual contact tracing efforts may improve the sensitivity and specificity of contact tracing [36].

Contact tracing effectiveness can be substantially increased with a reduction in contact tracing delays. In particular, we identified that this was the case for reducing tracing delays from three days to zero days (in effect, instantaneous contact tracing). We also identified that tracing delays of up to three days may still be effective, and that reducing tracing delays may allow trade-offs in terms of reducing coverage of contact tracing or quarantine duration. Timeliness of contact tracing is important in part because it is likely to be interdependent with contact tracing coverage: tracing a few contacts may be done quickly, but this is less likely when the number of contacts is high [76]. James et al. [76] found in their modelling study that with a mean tracing time higher than six days the benefit of tracing more contacts is very low and that faster tracing of those contacts who are easier to locate should be a priority.

Contact tracing with high coverage may be an important measure, in conjunction with other NPIs, to control the COVID-19 epidemic in schools and other educational institutions. The relevance of this assessment gains weight when one considers the negative impact of closing educational institutions, which includes economic losses to parents forced into childcare, educational losses and psychological harm to students [111].

In this review, empirical studies show that contact tracing can be effective in controlling the COVID-19 pandemic. These results are based on six studies, a small set. Interestingly, the mathematical studies included in this review described a plethora of highly effective contact interventions. This contrast may signal that the implementation of contact tracing interventions in the real world poses strong challenges not accounted for by modelling studies. Further, while the majority of the ecological studies exploring the effect of contact tracing in the context of other NPIs did not show a comparatively significant effect, contact tracing is defined in most of these studies as a policy with different levels of intensity/stringency. For example, in the widely used Oxford COVID-19 Government Response Tracker, these levels are “no contact tracing”, “limited contact tracing – not done for all cases”, and “comprehensive contact tracing – done for all identified cases” [112]. This is a general definition of contact tracing which may not accurately describe actual contact tracing implementation. Indeed, this definition does not provide information about, for example, the extent of contact tracing coverage. Thus, its use may be not be reflecting the real impact of specific contact tracing interventions on the pandemic. More empirical studies accounting for the actual extent of contact tracing implementation are required to address this issue.

A recent systematic review of the effectiveness of contact tracing interventions in the control of infectious diseases [113] concluded that, across eight diseases including COVID-19, HIV, several STIs and measles, provider-initiated contact tracing was associated with improvements in case detection, disease transmission, and incidence. In the case of COVID-19, based on four observational studies - three of which are included in our review [24, 30, 33], the authors highlight, like us, that contact tracing programmes can have effectiveness at mitigating disease spread [113]. The review also discusses some of the limitations of these studies. For example, all the studies were mostly undertaken in high resource settings and used observational designs with different programmatic approaches, hence limiting generalisability [113]. These limitations extend to the set of empirical studies included in this review.

This study has certain limitations. Due to the extent of the literature, we did not extend the focus of the review to the whole test-trace-quarantine process. Indeed, each of these three elements are linked and the failure of one of them may render the other two ineffective. For example, the effectiveness of testing suspected index cases, key for the contact tracing, can be hindered by, *inter alia*, low sensitivity and specificity of diagnostic tests, by insufficient capacity in the health care system for testing index patients, or by delays in testing index cases. Assuming contact tracing is highly effective, the effectiveness of quarantine can be affected by, *inter alia*, delays between contact tracing and quarantining, the length of the quarantine, and adherence of individuals to the quarantine. An adequate test-trace-quarantine process requires high levels of coordination between public health agents (those involved in surveillance, laboratory testing, monitoring and enforcing quarantines, communicating risks and rules) and a substantial economic investment, not to mention the collaboration of the public. An additional limitation is that we did not incorporate into the review all contact tracing mathematical modelling studies. Our focus on the higher quality studies incorporating more realistic modelling assumptions, particularly individual-based modelling and the realistic representation of social interactions, led to the exclusion of an important part of the literature: that of studies using compartmental dynamic transmission modelling. Finally, another limitation is that we included preprints in this review. Although preprints are not peer-reviewed, we assessed their quality using standard risk of bias tools and only included those with the highest quality.

To the best of our knowledge, at the time of writing there is no other systematic review of the comparative effectiveness of contact tracing interventions in the context of COVID-19 covering the literature until the summer solstice of 2021. Based on a limited number of observational studies, we found that there is evidence regarding the incremental effectiveness of both manual and digital contact tracing for COVID-19 epidemic control. The highest quality mathematical modelling studies available found that highly effective contact tracing interventions include: manual contact tracing with high tracing coverage and either medium-term immunity, highly efficacious isolation/quarantine and/ or physical distancing; hybrid manual and digital contact tracing with high app adoption, highly effective isolation/ quarantine and social distancing; secondary contact tracing; eliminating contact tracing delays; bidirectional contact tracing; contact tracing with high coverage in reopening educational institutions. We also highlighted the role of social distancing to enhance the effectiveness of some of these interventions in the context of 2020 lockdown reopening.

## Electronic supplementary material

Below is the link to the electronic supplementary material.

Supplementary Material 1

Supplementary Material 2
