# Supplementary material for: Comparative effectiveness of contact tracing interventions in the context of the COVID-19 pandemic: a systematic review
Source: Eur J Epidemiol. 2023 Feb 16;38(3):243–66. doi: 10.1007/s10654-023-00963-z (PMC9932408; doi:10.1007/s10654-023-00963-z)
Supplement: Supplementary file 1 — Supplementary Material 1 [file 10654_2023_963_MOESM1_ESM.pdf]

Comparative effectiveness of contact tracing interventions in the context of the COVID-19 pandemic: a systematic review – supplementary information file.

Annex 1. Embase and MedRxiv searches... 2

Annex 2. Risk of bias tool and results of the quality assessment.. 4
Annex 2.1. Risk of bias tool for ecological studies.. 4
Annex 2.2 Results of the quality assessment 7
Annex 2.2.1. Results of the quality assessment: ecological studies.. 7
Annex 2.2.2. Results of the quality assessment: cohort studies... 8
Annex 2.2.3. Results of the quality assessment: mathematical modelling studies included in the review.. 16
Annex 2.2.4. Results of the quality assessment: mathematical modelling studies excluded from the review.. 18

Annex 3. Overview of studies.. 20
Annex 3.1. Overview of empirical studies...20
Annex 3.2. Overview of mathematical modelling studies 21

Annex 4. Study type, characteristics and sources of data..29
Annex 4.1. Study design, modelling approach/ statistical analysis, and sources of data for the empirical studies..29
Annex 4.2. Representation of infection and disease, social interactions, and model parameters for the mathematical modelling studies...30

Annex 5. Expanded results: mathematical modelling studies 37

REFERENCES 55

Annex 1. Embase and MedRxiv searches.

1. Embase search. Performed 26 June 2021.

#1

('severe acute respiratory syndrome coronavirus 2':ti,ab OR 'severe acute respiratory syndrome coronavirus 2'/exp OR 'covid 19'/exp OR ncov*:ti,ab OR covid*:ti,ab OR 'sars cov 2':ti,ab OR 'sars-cov-2':ti,ab OR 'sars coronavirus 2':ti,ab OR 'sars coronavirus 2'/exp OR 'severe acute respiratory syndrome cov 2':ti,ab OR 'wuhan coronavirus':ti,ab OR 'wuhan seafood market pneumonia virus':ti,ab OR sars2:ti,ab OR '2019-ncov':ti,ab OR 'hcov-19':ti,ab OR 'novel 2019 coronavirus':ti,ab OR '2019 novel coronavirus*':ti,ab OR 'novel coronavirus 2019'/exp OR '2019 novel human coronavirus*':ti,ab OR 'human coronavirus 2019':ti,ab OR 'coronavirus disease-19':ti,ab OR 'corona virus disease-19':ti,ab OR 'coronavirus disease 2019':ti,ab OR 'coronavirus disease 2019'/exp OR 'corona virus disease 2019':ti,ab OR '2019 coronavirus disease':ti,ab OR 'novel coronavirus 2019*':ti,ab OR 'novel coronavirus disease 2019':ti,ab OR 'novel coronavirus infection 2019':ti,ab OR '2019 corona virus disease':ti,ab OR 'new coronavirus*':ti,ab OR 'coronavirus outbreak':ti,ab OR 'coronavirus epidemic':ti,ab OR 'coronavirus pandemic':ti,ab OR 'pandemic of coronavirus':ti,ab) AND [2020-2021]/py

#2

(('contact'/exp OR contact) AND trac* OR 'contact trac*' OR 'quarantine'/exp OR quarantine OR (('self'/exp OR self) AND isolat*) OR (case* AND find*) OR 'disease notif*' OR 'outbreak control') AND [2020-2021]/py

#3

(statist* OR analy* OR model* OR simul*) AND [2020-2021]/py

#4

(epidem* OR impact* OR effect* OR effica* OR contro* OR influenc* OR efficie* OR result*) AND [2020-2021]/py

#5

#1 AND #2 AND #3 AND #4 AND ([article]/lim OR [article in press]/lim OR [data papers]/lim OR [erratum]/lim OR [review]/lim) AND [english]/lim AND [humans]/lim AND ([embase]/lim OR [pubmed-not-medline]/lim)

2. MedRxiv search: Performed 7 July 2021 using the R package ‘medrrxiv’.

```
topic1 <- c("COVID", "COVID 19", "ncovid", "new coronavirus", "novel coronavirus",
        "SARS-CoV-2", "severe acute respiratory syndrome coronavirus 2",
        "sars cov 2", "sars coronavirus 2", "coronavirus outbreak",
        "coronavirus disease 2019")

topic2  <- c("contact", "tracing", "contact tracing", "test and trace", "quarantine",
         "self isolation", "self-isolation", "case finding", "case-finding",
         "outbreak control")

topic3 <- c("statistical", "statistic", "analysis", "model", "simulation")

topic4 <- c("epidemiology", "epidemiological", "impact", "impacts", "effect",
        "effects", "efficacy", "effectiveness", "control", "influence", "efficiency")
```

Annex 2. Risk of bias tool and results of the quality assessment.

Annex 2.1. Risk of bias tool for ecological studies.

1. Tool questions

Study design (max=7)	
Study design (max=2)	If it is a multi-level design (e.g. ecologic + individual), the study is upgraded one point (e.g. cross-sectional + multi-level receives 2 points)<br><br>Cross-sectional = 1 point, Longitudinal=2 points
Sample size (max = 2)	Number of ecologic units included in the analysis as proportion of the total number of units, e.g. 119 countries of a total of 195 worldwide would be 61%.<br><br>< 10% units = 0 points<br>10%-80% units = 1 point<br>≥ 80% units = 2 points
Level of inference (max = 1)	Use of the results of the analysis of the study's sample data to draw inferences for individuals or groups (ecologic).<br><br>Individual or unclear = 0 points<br>Ecologic = 1 point
Prespecification of ecological units (max = 1)	Where the ecologic units selected to suit the hypothesis? (as opposed to selection motivated by convenience or necessity)<br><br>No = 0 points<br>Yes = 1 point
Source of data (max=1)	Validity of the sources of data to represent the level that it refers to. If the source of data is not mentioned it is considered inadequate.<br><br>Inadequate = 0 points<br>Adequate = 1 point

Statistical methodology (max=8)	
Use of covariates (max=1)	Authors adjusted the analysis for desirable variables or not.<br><br>No = 0 point<br>Yes = 1 point
Proper adjustment for covariates (max=1)	Are the outcomes standardized or adjusted for certain factors before model adjustment? For standardized or adjusted outcomes, the standardized or adjusted factors should be included in the adjustment model. If standardized/adjusted outcomes are not used, this criterion is considered to have been met.<br><br>No = 0 points<br>Yes = 1 point
Validity of regression (max=1)	Did the adjustment have at least 10 units per covariate?<br><br>No = 0 points<br>Yes = 1 point
Spatial effects (max=1)	Inclusion of spatial analysis<br><br>No = 0 points<br>Yes = 1 point
Internal validity of the methodology (max=2)	Did the authors perform sensitivity analyses or robustness checks? Did they analyse the data through more than one independent method?<br><br>One method and no robustness check or sensitivity analysis = 0 points<br>One method and robustness check or sensitivity analysis OR more than one method and no robustness check or sensitivity analysis = 1 point<br>More than one method and robustness check or sensitivity analysis = 2 points
Analytical methodologies (max = 2)	All statistical methods are acceptable as long as they are used appropriately. We assign a score based on the sophistication and flexibility of the method.<br><br>1 point = less sophisticated, less flexible: correlational analysis, univariate or multivariate linear regression (no growth model), linear regression (growth model) with mixed effects or not, with or without transformation of outcome/response variables, interrupted time series.<br>2 points = more sophisticated and/or more flexible. More flexible: Generalised linear regression (growth model) - it is more flexible than linear regression with or without mixed effects because it allows to separate the modelling or linearity and

	variance relationships. More sophisticated: Bayesian models (hierarchical or not), step function lasso regression, transformer modelling, impulse-response analysis.
Quality of reporting (max=3)	
Statement of study design (max=1)	Did the authors present key elements of study design in the paper?<br><br>No = 0 points<br>Yes = 1 point
Justification of study design (max=1)	Did the authors justify the ecologic analysis, the rationale and the specific objectives, including any prespecified hypotheses?<br><br>No = 0 points<br>Yes = 1 point
Discussion cross-level bias and limitations (max=1)	Did the authors caution readers about the limitations of the ecologic design, the ecologic fallacy, the impossibility of extrapolating to a different level?<br><br>No = 0 points<br>Yes = 1 point

Annex 2.2 Results of the quality assessment.

Annex 2.2.1. Results of the quality assessment: ecological studies

	Study design (max=2)	Sample size (max =2)	Level of inference (max = 1)	Prespecification of ecological units (max = 1)	Source of data (max=1)	Use of covariates (max=1)	Proper adjustment for covariates (max=1)	Validity of regression (max=1)	Spatial effects (max=1)	Internal validity of the methodology (max=2)	Analytical methodologies (max = 2)	Statement of study design (max=1)	Justification of study design (max=1)	Discussion cross-level bias and limitations (max=1)	**Total (max=18)**
Wymant et al. [1]	2	2	1	1	1	1	1	1	0	2	2	1	1	1	**17**
Kendall et al. [2]	2	2	1	1	1	1	1	1	0	2	2	1	1	1	**17**
Pozo-Martín et al. [3]	2	2	1	1	1	1	1	1	0	1	2	1	1	1	**16**
Haug et al. [4]	2	1	1	1	1	1	1	1	0	2	2	1	1	1	**16**
Liu et al. [5]	2	1	1	1	1	0	1	1	0	2	2	1	1	1	**15**
Vecino-Ortiz et al. [6]	2	2	1	1	1	1	1	1	0	1	1	1	1	1	**15**
Leffler et al. [7]	2	2	1	1	1	1	1	0	0	0	1	1	1	1	**13**
Wibbens et al. [8]	2	1	1	0	1	0	1	1	0	1	2	1	1	1	**13**
Hong et al. [9]	2	1	1	0	1	0	1	0	0	1	1	1	1	1	**11**
Papadopoulos et al. [10]	2	1	1	1	1	0	1	0	0	0	1	1	1	1	**11**

Annex 2.2.2. Results of the quality assessment: cohort studies.

SIGN	Methodology Checklist 3: Cohort studies	
Study identification (*Include author, title, year of publication, journal title, pages*) **[11] Malheiro R, Figuereido AL, Magalhaes JP et al. Effectiveness of contact tracing and quarantine on reducing COVID-19 transmission: a retrospective cohort study. 2020. 189:54-9.DOI: 10.1016/j.puhe.2020.09.012**		
Guideline topic:	Key Question No:	Reviewer:

Before completing this checklist, consider:

1. Is the paper really a cohort study? If in doubt, check the study design algorithm available from SIGN and make sure you have the correct checklist.
2. Is the paper relevant to key question? Analyze using PICO (Patient or Population Intervention Comparison Outcome). IF NO REJECT (give reason below). IF YES complete the checklist.

Reason for rejection: 1. Paper not relevant to key question □ 2. Other reason □ (please specify):

Please note that a retrospective study (ie a database or chart study) cannot be rated higher than +.

Section 1: Internal validity		
In a well conducted cohort study:		***Does this study do it?***
1.1	The study addresses an appropriate and clearly focused question.<br>**This study aimed to assess the effectiveness of contact tracing and quarantine measures (in combination with case isolation) on reducing transmission of SARS-CoV-2 in Eastern Porto, Portugal, from March 1st, 2020 to May 15th, 2020.**	Yes □ No □<br>Can't say □
SELECTION OF SUBJECTS		
1.2	The two groups being studied are selected from source populations that are comparable in all respects other than the factor under investigation.<br>**Source population: COVID-19 cases notified to the Eastern Porto Public Health Authority. Intervention group: all COVID-19 cases identified as close contacts of an index case or under mandatory quarantine. Control group: all COVID-19 cases not subject to contact tracing and quarantine. Table 1 compares the sociodemographic characteristics of the intervention and control group. These were similar except for the exposure status, the total number of close contacts, the number of household members and the time between symptom onset and notification/ sample collection**	Yes □ No □<br>Can't say □ Does not apply □

1.3	The study indicates how many of the people asked to take part did so, in each of the groups being studied.<br><br>**Of 630 COVID-19 cases reported to the public health authority during the study period, 551 (87.5%) were considered for the analysis. The intervention and control cohorts comprised 98 (17.8%) and 453 (82.2%) cases, respectively. Most excluded cases were nursing homes' residents (45), followed by uncontacted (17) and hospitalized patients. The participation rate per group is not clearly specified.**	Yes □	No □<br><br>Does not apply □
1.4	The likelihood that some eligible subjects might have the outcome at the time of enrolment is assessed and considered in the analysis.<br><br>**Primary outcome: median number of secondary cases by index case and the proportion of cases with secondary cases. Secondary outcome: median time from symptom onset to specimen collection and median number of close contacts. Both outcomes may show exposure at the moment of selection in some eligible subjects, so there is a possible performance bias.**	Yes □<br><br>Can't say □	No □<br><br>Does not apply □
1.5	What percentage of individuals or clusters recruited into each arm of the study dropped out before the study was completed.<br><br>**The percentage of individuals dropping out is not specified, but it is likely to be small given the nature and the short duration of the study.**	Not specified, likely to be small	
1.6	Comparison is made between full participants and those lost to follow up, by exposure status.<br><br>**Unclear, but lost to follow-up is likely to be small and it is unlikely that there is asymmetry in loss to follow-up between the intervention and control groups**	Yes □<br><br>Can't say □	No □<br><br>Does not apply □
ASSESSMENT			
1.7	The outcomes are clearly defined.<br><br>**Primary outcome: median number of secondary cases by index case and the proportion of cases with secondary cases. Secondary outcome: median time from symptom onset to specimen collection and median number of close contacts.**	Yes □<br><br>Can't say □	No □

1.8	The assessment of outcome is made blind to exposure status. If the study is retrospective this may not be applicable<br>**Exposure Status (ES): contact tracing or quarantine. Due to the nature of the study, blind assessment of outcome was not possible**.	Yes □ No □<br>Can't say □ <u>Does not apply</u> □
1.9	Where blinding was not possible, there is some recognition that knowledge of exposure status could have influenced the assessment of outcome.<br>**Not mentioned in the paper**	Yes □ <u>No</u> □<br>Can't say □
1.10	The method of assessment of exposure is reliable	<u>Yes</u> □ No □<br>Can't say □
1.11	Evidence from other sources is used to demonstrate that the method of outcome assessment is valid and reliable	Yes □ <u>No</u> □<br>Can't say □ Does not apply□
1.12	Exposure level or prognostic factor is assessed more than once	Yes □ No □<br><u>Can't say</u> □ Does not apply □
CONFOUNDING		
1.13	The main potential confounders are identified and taken-into-account in the design and analysis.<br>**The authors repeated the analysis adjusting for lockdown measures.**	<u>Yes</u> □ No □<br>Can't say □
STATISTICAL ANALYSIS		
1.14	Have confidence intervals been provided?	<u>Yes</u> □ No □

SECTION 2: OVERALL ASSESSMENT OF THE STUDY			
2.1	How well was the study done to minimise the risk of bias or confounding?<br><br>**In order to control for the lockdown effect outcomes are analysed in the period before and after lockdown. A stratified (but not multivariable) análisis is done adjusting for lockdown period.**	High quality (++) □<br>Acceptable (+) □<br>Unacceptable – reject 0	
2.2	Taking-into-account clinical considerations, your evaluation of the methodology used, and the statistical power of the study, do you think there is clear evidence of an association between exposure and outcome?	Yes ☐<br>Can't say ☐	No ☐
2.3	Are the results of this study directly applicable to the patient group targeted in this guideline?	Yes □	No □
2.4	**Notes.** Summarise the authors conclusions. Add any comments on your own assessment of the study, and the extent to which it answers your question and mention any areas of uncertainty raised above.		
	**In the present study, contact tracing and quarantine of close contacts appear to have no role in reducing the number of secondary cases of COVID-19.**<br><br>**Local public health measures (contact tracing &quarantine) are effective at reducing both the time between symptom onset and laboratory diagnosis and the median number of close contacts per case. No effect was apparent on secondary cases figures, suggesting that further measures may be required to break the transmission chain of COVID-19. Nevertheless, national restriction measures appear to have an impact on reducing transmission of SARS-CoV-2.**<br><br>**The results of this study should be considered with precaution, as there are possible selection, performance and detection biases. In addition, there are relatively few cases particularly in the intervention group (n=98). Further studies with more cases and more robust designs are needed.**		

SIGN	Methodology Checklist 3: Cohort studies

Study identification (*Include author, title, year of publication, journal title, pages*) **[12] Park Y, Huh IS, Lee J, Kang CR, Cho S-i, Ham HJ, et al. Application of Testing-Tracing-Treatment Strategy in Response to the COVID-19 Outbreak in Seoul, Korea. J Korean Med Sci. 2020;35(45)**

Guideline topic:	Key Question No:	Reviewer:

Before completing this checklist, consider:

1. Is the paper really a cohort study? If in doubt, check the study design algorithm available from SIGN and make sure you have the correct checklist.
2. Is the paper relevant to key question? Analyze using PICO (Patient or Population Intervention Comparison Outcome). IF NO REJECT (give reason below). IF YES complete the checklist.

Reason for rejection: 1. Paper not relevant to key question □ 2. Other reason □ (please specify):

Please note that a retrospective study (ie a database or chart study) cannot be rated higher than +.

Section 1: Internal validity		
In a well conducted cohort study:		***Does this study do it?***
1.1	The study addresses an appropriate and clearly focused question.<br>**The study compares the epidemiological and clinical outcomes between two cohorts of individuals: 1) contacts of clusters associated with COVID-19 cases and individuals with COVID-19 symptoms, who undergo COVID-19 testing and isolation if testing positive; 2) individuals with COVID-19 symptoms who undergo COVID-19 testing and isolation if testing positive.**	<u>Yes</u> □ No □<br>Can't say □
SELECTION OF SUBJECTS		
1.2	The two groups being studied are selected from source populations that are comparable in all respects other than the factor under investigation.<br>**Source population: Individuals with COVID-19 symptoms and individuals who have been in contact with COVID-19 cases in Seoul until May 2, 2020. Intervention group: COVID-19 cases identified after testing contacts of COVID-19 case clusters and symptomatic individuals from March 9, 2020. Comparison group: COVID-19 cases identified through testing of symptomatic individuals from January 10, 2020, whose contacts are traced. Table 1 compares the sociodemographic characteristics of the two groups. Age, sex, source of infection and clinical status differ significantly.**	Yes □ <u>No</u> □<br>Can't say □ Does not apply □
1.3	The study indicates how many of the people asked to take part did so, in each of the groups being studied.	Yes □ <u>No</u> □<br>Does not apply □

1.4	The likelihood that some eligible subjects might have the outcome at the time of enrolment is assessed and considered in the analysis.<br>**Outcome measured: forward transmission of COVID-19. Measurement of exposure is likely objective and consistent in intervention as well as in comparison group. There is little difference in management between the two groups, so there should not be too much performance bias**	Yes □ <ins>No</ins> □<br>Can't say □ Does not apply □
1.5	What percentage of individuals or clusters recruited into each arm of the study dropped out before the study was completed.<br>**The percentage of individuals dropping out is not specified, but it is likely to be small given the nature and the short duration of the study.**	Not specified, likely to be small
1.6	Comparison is made between full participants and those lost to follow up, by exposure status.<br>**Unclear, but losses to follow-up are likely to be small.**	Yes □ No □<br><ins>Can't say</ins> □ Does not apply □
ASSESSMENT		
1.7	The outcomes are clearly defined.<br>**Main outcome relevant to our review: Forward transmission of COVID-19**	<ins>Yes</ins> □ No □<br>Can't say □
1.8	The assessment of outcome is made blind to exposure status. If the study is retrospective this may not be applicable	Yes □ No □<br>Can't say □ <ins>Does not apply</ins> □
1.9	Where blinding was not possible, there is some recognition that knowledge of exposure status could have influenced the assessment of outcome.<br>**Not mentioned in the paper**	Yes □ <ins>No</ins> □<br>Can't say □

1.10	The method of assessment of exposure is reliable	Yes □<br>Can't say □	No □
1.11	Evidence from other sources is used to demonstrate that the method of outcome assessment is valid and reliable	Yes □<br>Can't say □	No □<br>Does not apply□
1.12	Exposure level or prognostic factor is assessed more than once	Yes □<br>Can't say □	No □<br>Does not apply □
CONFOUNDING			
1.13	The main potential confounders are identified and taken-into-account in the design and analysis.<br>**The authors did not adjust the analysis for potential confounders**	Yes □<br>Can't say □	No □
STATISTICAL ANALYSIS			
1.14	Have confidence intervals been provided?<br>**The authors do not provide confidence intervals for the changes in the reproduction number**	Yes □	No □
**SECTION 2: OVERALL ASSESSMENT OF THE STUDY**			
2.1	How well was the study done to minimise the risk of bias or confounding?	High quality (++) □<br>Acceptable (+) □<br>Unacceptable – reject 0	
2.2	Taking-into-account clinical considerations, your evaluation of the methodology used, and the statistical power of the study, do you think there is clear evidence of an association between exposure and outcome?	Yes ☐<br>Can't say ☐	No ☐
2.3	Are the results of this study directly applicable to the patient group targeted in this guideline?	Yes □	No □

2.4	**Notes.** Summarise the authors conclusions. Add any comments on your own assessment of the study, and the extent to which it answers your question and mention any areas of uncertainty raised above.
	**In the present study, tracing and testing contacts COVID-19 case clusters and symptomatic individuals and quarantining all those testing positive decreased the COVID-19 reproduction rate from 1.3 to 0.6 (no effect size was provided). Preemptive testing and prompt contact tracing are effective measures for COVID-19 epidemic control.**<br><br>**The results of this study should be considered with precaution, as no measure of effect was provided, no assessment of potential confounders was done and there is possible selection bias.**

Annex 2.2.3. Results of the quality assessment: mathematical modelling studies included in the review.

Study	Is there a transparent presentation of the model?<br><br>Yes=1<br>No=0	Are the parameters and their sources fully described?<br><br>Yes=1<br>No=0	Are sensitivity analyses on the main model assumptions explored?<br><br>Yes=1<br>No=0	Does the model distinguish between different categories of infectiousness?<br><br>Yes=1<br>No=0	Is the model an individual simulation?<br><br>Yes=3<br>No=0	Is there social mixing or a multi-layer network?<br><br>Yes (multi-layer network) = 2<br>Yes (social mixing) = 1<br>No = 0	Final score
**Agent-Based Models (ABM):**							
Abueg et al. [13]	1	1	1	1	3	2	9
Aleta et al. [14]	1	1	1	1	3	2	9
Bicher et al. [15]	1	1	1	1	3	2	9
Li et al. [16]	1	1	1	1	3	2	9
Gressman et al. [17]	1	1	1	1	3	2	9
Hill et al. 1 [18]	1	1	1	1	3	2	9
Hill et al. 2 [19]	1	1	1	1	3	2	9
Kerr et al. [20]	1	1	1	1	3	2	9
Luo et al. [21]	1	1	1	1	3	2	9
Moreno López et al. [22]	1	1	1	1	3	2	9
Ng et al. [23]	1	1	1	1	3	2	9
Pham et al. [24]	1	1	1	1	3	2	9
Scott et al. [25]	1	1	1	1	3	2	9
Shamil et al. [26]	1	1	1	1	3	2	9
Stuart et al. [27]	1	1	1	1	3	2	9
Tatapudi et al. [28]	1	1	1	1	3	2	9
Thompson et al. [29]	1	1	1	1	3	2	9
Wells et al. [30]	1	1	1	1	3	2	9
Willem et al. [31]	1	1	1	1	3	2	9
Barthe et al. [32]	1	1	1	1	3	1	8
Bhattacharyya et al. [33]	1	1	0	1	3	2	8
Colomer et al. [34]	1	1	1	1	3	1	8
Eilersen et al. [35]	1	1	2	1	3	2	8
Goldberg et al. [36]	1	1	1	1	3	1	8
Goldenbogen et al. [37]	1	1	1	0	3	2	8
Low et al. [38]	1	0	1	1	3	2	8
Moon et al. [39]	1	1	1	0	3	2	8
Mukherjee et al. [40]	1	1	1	1	3	1	8
Panovska-Griffiths et al. [41]	1	0	1	1	3	2	8
Pollmann et al. [42]	1	1	1	1	3	1	8
Fiore et al. [43]	0	1	1	1	3	1	7
Geffen et al. [44]	1	1	1	1	3	0	7
Quilty et al. [45]	1	1	1	1	3	0	7
Tuomisto et al. [46]	1	1	1	1	3	0	7
Wallentin et al. [47]	1	1	1	0	3	1	7
Zafarnejad et al. [48]	1	1	1	1	3	0	7
Reich et al. 1 [49]	1	1	1	0	3	0	6
Reich 2 [50]	1	0	1	1	3	0	6
**Stochastic Branching Process Models (SBP):**							
Plank et al. [51]	1	1	1	1	3	2	9
Allali et al. [52]	1	1	1	1	3	0	7
Bradshaw et al. 1 [53]	1	1	1	1	3	0	7
Bradshaw et al. 2 [54]	1	1	1	1	3	0	7
Brook et al. [55]	1	1	1	1	3	0	7
Davis et al. [56]	1	1	1	1	3	0	7
Filonets et al. [57]	1	1	1	1	3	0	7
Firth et al. [58]	1	1	1	1	3	0	7
Fyles et al. [59]	1	1	1	1	3	0	7
Hellewell et al. [60]	1	1	1	1	3	0	7
Huamani et al. [61]	1	1	1	1	3	0	7
James et al. [62]	1	1	1	1	3	0	7
Kinoshita et al. [63]	1	1	1	1	3	0	7
Kretschmar et al. 1 [64]	1	1	1	1	3	0	7
Kretschmar et al. 2 [65]	1	1	1	1	3	0	7
Ng et al. [66]	1	1	1	1	3	0	7
Endo et al. [67]	1	1	1	0	3	0	6
Huang et al. [68]	1	1	1	0	3	0	6
Peak et al. [69]	0	1	1	1	3	0	6

Annex 2.2.3. (cont.): Results of the quality assessment: mathematical modelling studies included in the review.

Study	Is there a transparent presentation of the model?<br><br>Yes=1<br>No=0	Are the parameters and their sources fully described?<br><br>Yes=1<br>No=0	Are sensitivity analyses on the main model assumptions explored?<br><br>Yes=1<br>No=0	Does the model distinguish between different categories of infectiousness?<br><br>Yes=1<br>No=0	Is the model an individual simulation?<br><br>Yes=3<br>No=0	Is there social mixing or a multi-layer network?<br><br>Yes (multi-layer network) = 2<br>Yes (social mixing) = 1<br>No = 0	Final score
**Other models:**							
Cencetti et al. [70]	1	1	1	1	3	2	9
Kucharski et al. [71]	1	1	1	1	3	2	9
Ferretti et al. [72]	1	1	1	1	3	0	7
Grassly et al. [73]	1	1	1	1	3	0	7
Sanche et al. [74]	1	1	1	1	3	0	7
Kuzdeuov et al. [75]	1	0	1	1	3	0	6
Moran et al. [76]	0	1	1	1	3	0	6
Worden et al. [77]	1	1	1	0	3	0	6
Kim et al. [78]	1	1	1	0	3	0	6

Annex 2.2.4. Results of the quality assessment: mathematical modelling studies excluded from the review.

Study	Is there a transparent presentation of the model?<br><br>Yes=1<br>No=0	Are the parameters and their sources fully described?<br><br>Yes=1<br>No=0	Are sensitivity analyses on the main model assumptions explored?<br><br>Yes=1<br>No=0	Does the model distinguish between different categories of infectiousness?<br><br>Yes=1<br>No=0	Is the model an individual simulation?<br><br>Yes=3<br>No=0	Is there social mixing or a multi-layer network?<br><br>Yes (multi-layer network) = 2<br>Yes (social mixing) = 1<br>No = 0	Final score
**Agent-Based Models (ABM):**							
Rajabi et al. [79]	1	0	1	0	3	0	5
Sarma et al. [80]	1	0	1	0	3	0	5
**Stochastic Branching Process Models (SBP):**							
Pung et al. [81]	0	1	1	0	3	0	5
**Other models:**							
Scarabel et al. [82]	1	1	1	1	0	1	5
Serafino et al. [83]	0	0	1	1	3	0	5
Ashcroft et al. [84]	1	1	1	1	0	0	4
Bilinski et al. [85]	1	1	1	1	0	0	4
Killeen et al. [86]	1	1	1	1	0	0	4
Ponte et al. [87]	1	1	1	1	0	0	4
Segal et al. [88]	1	1	1	0	0	0	3
Braun et al. [89]	1	0	1	0	0	0	2
Jeon et al. [90]	0	0	1	1	0	0	2
Lambert et al. [91]	1	0	0	1	0	0	2
Malmberg et al. [92]	0	1	1	0	0	0	2
Afzal et al. [93]	1	0	0	0	0	0	1
**Dynamic transmission models (DTM):**							
Bracis et al. [94]	1	1	1	1	0	1	5
Di Domenico et al. [95]	1	1	1	1	0	1	5
Fair et al. [96]	1	1	1	1	0	1	5
Ferrari et al. [97]	1	1	1	1	0	1	5
Forgoston et al. [98]	1	1	1	1	0	1	5
Gosce et al. [99]	1	1	1	1	0	1	5
Grimm et al. [100]	1	1	1	1	0	1	5
Lunz et al. [101]	1	1	1	1	0	1	5
Min et al. [102]	1	1	1	1	0	1	5
Struben et al. [103]	1	1	1	1	0	1	5
Teimuri et al [104]	1	1	1	1	0	1	5
Zhong et al. [105]	1	1	1	1	0	1	5
Amaku et al. [106]	1	1	1	1	0	0	4
Amaku et al. [107]	1	1	1	1	0	0	4
Ansah et al. [108]	1	1	1	1	0	0	4
Barrat et al. [109]	1	1	1	1	0	0	4
Burdinski et al. [110]	1	1	1	1	0	0	4
Currie et al. [111]	1	1	1	1	0	0	4
Drake et al. [112]	1	1	1	1	0	0	4
Giordano. [113]	1	1	1	1	0	0	4
Grantz et al. [114]	1	1	1	1	0	0	4
Humphrey et al. [115]	1	1	1	1	0	0	4
Johnson et al. [116]	1	1	1	1	0	0	4
Keeling et al. [117]	1	1	1	0	0	1	4
Müller et al. [118]	1	1	1	0	0	1	4
Nakamoto et al. [119]	1	1	1	1	0	0	4
Ngonghala et al. [120]	1	1	1	1	0	0	4
Pandey et al. [121]	1	1	1	0	0	1	4
Prabhakaran et al. [122]	1	1	1	1	0	0	4
Proverbio et al. [123]	1	1	1	1	0	0	4
Rusu et al. [124]	1	1	1	1	0	0	4
Taboe et al. [125]	1	1	1	1	0	0	4
Torneri et al. [126]	1	1	1	1	0	0	4
Tuite et al. [127]	1	1	1	0	0	1	4
Wilson et al. 1 [128]	1	1	1	1	0	0	4
Wilson et al. 2 [129]	1	1	1	1	0	0	4
Yu et al. [130]	1	1	1	1	0	0	4
Bhadauria et al. [131]	1	1	1	0	0	0	3
Browne et al. [132]	1	1	1	0	0	0	3
Gardner et al. [133]	1	1	1	0	0	0	3

Annex 2.2.4 (cont): Results of the quality assessment: mathematical modelling studies excluded from the review.

Study	Is there a transparent presentation of the model?<br><br>Yes=1<br>No=0	Are the parameters and their sources fully described?<br><br>Yes=1<br>No=0	Are sensitivity analyses on the main model assumptions explored?<br><br>Yes=1<br>No=0	Does the model distinguish between different categories of infectiousness?<br><br>Yes=1<br>No=0	Is the model an individual simulation?<br><br>Yes=3<br>No=0	Is there social mixing or a multi-layer network?<br><br>Yes (multi-layer network) = 2<br>Yes (social mixing) = 1<br>No = 0	Final score
**Dynamic transmission models (DTM):**							
Kempf et al. [134]	1	1	1	0	0	0	3
Kurita et al. [135]	1	1	1	0	0	0	3
Li et al. [136]	1	1	1	0	0	0	3
Nuckchady et al. [137]	0	1	1	1	0	0	3
Nuzzo et al. [138]	1	1	1	0	0	0	3
Siraj et al. [139]	1	1	1	0	0	0	3
Wang et al. [140]	1	1	1	0	0	0	3
Zu et al. [141]	1	1	1	0	0	0	3

Annex 3. Overview of studies.

Annex 3.1. Overview of empirical studies.

Authors and outcomes assessed	Setting (country, regions)	Type of study	Intervention(s) and comparator(s)
Haug et al. [4]<br>Reff	79 territories and 46 countries worldwide	Ecological	42,151 different NPIs including contact tracing. Interventions are compared to each other
Hong et al. [9]<br>Growth rate in cumulative cases.	108 countries	Ecological	Assembly Restrictions (A): School closures (A1), Workplace closures (A2), Cancel public events (A3), Gathering size restriction (A4); Movement Restrictions (M): Close public transport (M1), Stay at home requirement (M2), Internal movement restrictions (M3), International travel restrictions (M4); Privacy Restriction (P): Contact tracing (P1). Interventions compared with each other
Kendall et al. [2]<br>Incidence, R	Isle of Wight	Ecological	Manual contact tracing + contact tracing using automated app. Comparator: Manual contact tracing
Leffler et al [7]<br>Deaths	200 countries	Ecological	(1) School closing; (2) Workplace closing; (3) Cancelling of public events; (4) Restrictions on gatherings; (5) Public transport closure; (6) Stay-at-home requirements; (7) Internal movement restrictions; (8) International travel restrictions; (9) Income support; (10) Public information campaigns; (11) Testing policy; (12) Contact tracing policy; (13) Mask-wearing
Liu et al. [5]<br>R	130 countries worldwide	Ecological	(1) Internal containment and closure (School and workplace closure, public event cancellation, limits on gathering sizes, public transport closure, stay-at-home requirement, internal movement restriction); (2) International travel restrictions; (3) Economic policies; (4) Health systems policies (Public information campaign, testing policy, contact tracing). Interventions are compared with each other
Papadopoulos et al. [10]<br>Number of cases, deaths	137 countries worldwide	Ecological	(1) School closing; (2) Workplace closing; (3) Cancelling of public events; (4) Restriction on gatherings; (5) Closure of public transport; (6) Stay-at-home restrictions; (7) Domestic travel restrictions; (8) International travel restrictions; (9) Public information; (10) Testing framework; (11) Contact tracing. Interventions are compared with each other
Pozo-Martin et al. [3]<br>Growth rate in cumulative cases	37 OECD countries	Ecological	(1) School closing requirements; (2) Workplace closing requirements; (3) Public events cancelling requirements; (4) Restrictions on gatherings; (5) Public transport restrictions; (6) Stay-at-home requirements; (7) Restrictions on internal travel; (8) International travel controls; (9) Public health information campaigns; (10) Mask wearing requirements; (11) Testing policy; (12) Contact tracing policy. Interventions are compared with each other.
Vecino-Ortiz et al. [6]<br>Deaths	32 departments and 5 districts in Colombia	Ecological	Contact tracing as implemented in different departments and districts
Wibbens et al. [8]<br>Growth rate in cases	40 territories: 17 countries and 23 US states	Ecological	(1) Closing of schools; (2) Closing of workplaces; (3) Public event cancelling; (4) Gathering bans; (5) Public transport closure; (6) Shelter-in-place orders and home confinement; (7) Restrictions on internal movement; (8) Restrictions on international travel; (9) Public information campaigns; (10) Testing access; (11) Contact tracing. Interventions are compared with each other.
Wymant et al. [1]<br>Number of cases, deaths	England and Wales	Ecological	Contact tracing using National Health Service (NHS) COVID-19 app. Comparator: no app
Malheiro et al. [11]<br>Secondary attack rate	Eastern Porto (Portugal)	Cohort study	(1) Contact tracing and quarantine. Comparator: no contact tracing and quarantine
Park et al. [12]<br>R	Seoul (South Korea)	Pre-post study	(1) Tracing the contacts of COVID-19 case clusters and symptomatic individuals, testing them and placing all those testing positive in quarantine. Comparator: Testing only symptomatic, tracing and testing their contacts and quarantining all those testing positive

Annex 3.2. Overview of mathematical modelling studies.

Authors and Outcomes assessed	Setting (country, regions); Period of the epidemic modelled	Type of modelling approach	Intervention(s) and comparator(s)
Abueg et al. [13]<br>Newly and total infected, total deaths, number hospitalized.	Three counties in Washington state (USA); 2020 lockdown reopening, social distancing	ABM	(1) Digital contact tracing of 1st order contacts - 90% probability of quarantine; (2) Manual contact tracing – likelihood of tracing household/ workplace/ random contacts = 100% / 80% / 5% (3) Intervention (1) combined with Intervention (2). Comparator: no intervention
Aleta et al. [14]<br>Daily and cumulative infection incidence, R_{eff}, number hospitalized.	Boston (USA); 2020 lockdown reopening, no social distancing	ABM	(1) After 8 weeks of lockdown, reopening of all work and community places except mass-gathering environments (4 weeks) followed by full reopening; (2) Intervention (1) + testing of symptomatic individuals (30% and 50% successfully diagnosed), tracing (varying fraction of non-household contacts) and quarantining (household and non-household contacts). Strategies are compared to the unmitigated scenario in which no interventions are implemented
Barthe et al. [32]<br>Number of infections	Tübingen (Germany); Epidemic from first cases, context other than 2020 lockdown reopening, no social distancing	ABM	(1) Implementation of PanCast, which uses bluetooth beacons strategically located to match IDs of contacts with IDs of positive-testing individuals + manual contact tracing – this results in bidirectional contact tracing, and (2) Digital contact tracing (smartphone-based, pairwise encounter-based) + manual contact tracing. Comparator: manual contact tracing
Bhattacharyya et al. [33]<br>Percentage of infected population, percentage of recovered, number of deaths per million	Bengaluru (India); Epidemic from first cases, context other than 2020 lockdown reopening	ABM	(1) Lockdown, (2) Mild lockdown, (3) fixed duration lockdown, (4) Two-day week followed by strict lockdown, (5) Tracing and quarantining neighbours (and neighbours of neighbours) of infected patients, (6) Quarantining of city ward and reopening with different relaxation factors. Several mixes of policies are also considered. Interventions are compared to each other and to no intervention
Bicher et al. [15]<br>Active and cumulative number of confirmed cases, R_{eff},	Austria; 2020 lockdown reopening, social distancing	ABM	(1) Tracing of household members; (2) Tracing of co-workers; (3) Interventions (1) + (2); (4) Intervention (3) + tracing of individual contacts (50% accuracy); (5) Intervention (3) + tracing of individual contacts (75% accuracy). The interventions are compared to no contact tracing and to each other assuming other measures are in place that achieve high (74.58%) infectivity reduction
Colomer et al. [34]<br>Number of deaths, recovered	Spain; Ongoing epidemic in a context other than 2020 lockdown reopening, with and without social distancing/ vaccination	ABM	(1) Varying levels of vaccination, 2) varying levels of contact tracing, 3) varying levels of vaccination and contact tracing with and without implementing other non-pharmaceutical interventions (social distancing + hand washing + mask wearing). Different levels of implementation of strategies (1)-(3) with and without other non-pharmaceutical interventions are compared to no tracing
Eilersen et al. [35]<br>Peak number of infected	Denmark; Epidemic from first cases in a context other than 2020 lockdown reopening/ reopening after 30-day lockdown at the beginning of the epidemic	ABM	(1) One-step tracing and quarantining – i.e. isolation of agents with positive test results, closure of their workplaces, identification and quarantining of their regular social contacts with different levels of testing and quarantine duration. Comparator: no intervention
Fiore et al. [43]<br>R_{eff}	New York (USA), Southeast Italy, Midlands (UK); Epidemic from first cases in a context other than 2020 lockdown reopening, social distancing	ABM	(1) Contact tracing, testing and isolation if testing positive for different levels of testing efficacy and contact tracing coverage and testing (20%, 40%, 60%, 80%, and100%). Different scenarios were modelled for disease incidence (equivalent to a daily growth in cases of 15%, 25% or 35%) to represent alternative physical distancing policies. Comparator: no intervention

Annex 3.2 (cont.). Overview of mathematical modelling studies.

Authors and Outcomes assessed	Setting (country, regions); Period of the epidemic modelled	Type of modelling approach	Intervention(s) and comparator(s)
Geffen et al. [44]<br>Mean total number of infections	Epidemic from first cases in a context other than 2020 lockdown reopening, no social distancing	ABM	(1) Isolation of agents with positive test results (85% mean adherence per day); (2) isolation of agents with positive tests results (85% mean adherence per day) + 10% of their contacts are traced and isolated (85% mean adherence per day), (3) isolation of agents with positive results (85% adherence per day), and 30% of their contacts are traced and isolated (85% mean adherence per day), (4) perfect isolation of agents when they test positive and perfect tracing of first and secondary contacts, who are then also perfectly isolated. Comparator: no isolation, no contact tracing
Goldberg et al. [36]<br>Epidemic size, hospital load and number of days in quarantine	Not stated;<br>Epidemic from first cases in a context other than 2020 lockdown reopening, no social distancing	ABM	Digital contact tracing with user referrals under four scenarios: (1) app users are a random subset of population, (2) randomly selected users recommend the app to one contact, (3) randomly selected initial users recommend the app to its neighbours, and these to about 50% of contacts, and (4) app users are individuals with highest number of connections. All users notify contacts when they have symptoms. The interventions are compared to each other
Goldenbogen et al. [37]<br>Number of cases	Two communities in Germany, one in England, one in Sweden;<br>2020 lockdown reopening, with/without social distancing	ABM	(1) co-location recursive contact tracing (identifying individuals spatio-temporal overlap), (2) contact-based tracing (identifying actual interactions). Comparator: The interventions are compared to each other and to no intervention
Gressman et al. [17]<br>Cumulative infections	USA;<br>Reopening education institutions after 2020 lockdown	ABM	(1) "Standard intervention" = Daily random testing of 3% of students and of contacts identified the previous day + quarantining al students who test positive and all symptomatic with 100% success rate + contact tracing of all positive tested and all symptomatic + transitioning all classes with 30 or more students to online-only interaction + mask wearing (50% reduction of transmission) + 5% immunity at start of term, (2) Excluding testing from "standard intervention", 3) excluding contact tracing. Interventions are compared to each other and to no intervention
Hill et al. 1 [18]<br>Proportion of students infected	A University in the UK;<br>Reopening after 2020 lockdown in educational institutions	ABM	(1) contact tracing with recall decay and varying levels of trace and quarantine efficacy (0%-100%). Comparator: no intervention
Hill et al. 2 [19]<br>Outbreak size, peak infectious prevalence	A worker population in the UK;<br>Epidemic from first cases in a context other than 2020 lockdown reopening	ABM	(1) contact tracing with recall decay and varying levels of trace and quarantine efficacy (0%-100%). Comparator: no intervention
Kerr et al. [20]<br>Attack rate (%) and epidemic trends.	One county in Seattle, Washington (USA);<br>2020 lockdown reopening with mask wearing	ABM	(1) Testing, tracing, quarantining with a variable range of quarantine effectiveness, routine testing probability, swab-to-result delay, contact tracing probability, quarantine testing probability, and contact tracing delay. Intervention (1) is implemented under 3 scenarios: 60%, 80% and 100% mobility. Comparator: Baseline scenario is a high mobility (100%) scenario with high testing (6000 tests per day) and high tracing (70% of all household and workplace contacts within two days).
Li et al. [16]<br>Number of new infections.	USA;<br>Ongoing epidemic in a context other than lockdown reopening, with other NPIs	ABM	(1) mask wearing, (2) school closing, (3) work closing, (4) social distancing, (5) testing, (6) contact tracing and (7) quarantining.<br>The interventions are compared to each other

Annex 3.2 (cont.). Overview of mathematical modelling studies.

Authors and Outcomes assessed	Setting (country, regions); Period of the epidemic modelled	Type of modelling approach	Intervention(s) and comparator(s)
Low et al. [38]<br>Mean number of infections	A township in Cape Town (SA);<br>Epidemic from first cases in a context other than 2020 lockdown reopening, no social distancing	ABM	(1) No contact tracing with a test turnaround time of two days, (2) contact tracing with a test turnaround time of two days, and (3) contact tracing with a test turnaround time of eight days. Comparator: No contact tracing with a test turnaround time of two days
Luo et al. [21]<br>Density of infection (per 10,000 population)	Wuhan (China);<br>Epidemic from first cases in a context other than 2020 lockdown reopening	ABM	(1) Contact tracing of symptomatic and asymptomatic cases with variable rate of coverage of asymptomatic cases (0%-100%); (2) Different delays in implementing contact tracing (30,50,70,90 days after the start of the epidemic). Comparator: no contact tracing
Moon et al. [39]<br>Number of cases	Manhattan, a town in Kansas (USA);<br>2020 lockdown reopening, social distancing	ABM	(1) Varying levels of contact tracing, testing + isolation of infected contacts of a confirmed case, (2) Varying levels of contact tracing, testing + isolation of all contacts of a confirmed case. Comparator: no contact tracing
Moreno López et al. [22]<br>Attack rate and peak incidence	France;<br>2020 lockdown reopening, social distancing	ABM	(1) Combined testing (50% of symptomatic individuals), self-isolation and isolation of household contacts + digital contact tracing with different levels of app adoption. Comparator: no intervention, self-isolation and isolation of household contacts
Mukherjee et al. [40]<br>Average number of susceptible individuals as a fraction of the total population	86 universities in USA;<br>Reopening education institutions after 2020 lockdown	ABM	Combinations of (1) random testing and (2) contact tracing (results of tests are available the same day). The interventions are compared to each other.
Ng et al. [23]<br>Attack rate, hospital admissions, ICU admissions and mortality rate	Canada;<br>2020 lockdown reopening, with and without social distancing	ABM	Different combinations of case detection and isolation, contact tracing and quarantine, physical distancing and community closures. Relevant for contact tracing are: (1) Contact tracing and quarantine (20% of cases detected, 50% of contacts of these cases traced and quarantined), (2) Enhanced case detection and contact tracing (50% of cases detected and isolated with 50% of household members co-isolating + 100% of these cases traced and quarantined), (3) Enhanced case detection and contact tracing with physical distancing interventions (20% reduction in the contact rate). The interventions are compared to each other and to no intervention.
Panovska-Griffiths et al. [41]<br>Number of new infections, deaths, R_{eff}, individuals to be tested to avoid a second wave	UK;<br>2020 lockdown reopening, some social distancing	ABM	(1) Full-time school reopening, 18% testing of symptomatic (TS), 68% tracing of all contacts (TAC); (2) Full-time school reopening, 75% TS, 68% TAC; (3) Full-time school reopening, 87% TC, 40% TAC; (4) Part-time school reopening, 18% TS, 68% TAC; (5) Part-time school reopening, 65% TS, 68% TAC; (6) Part-time school reopening, 75% TS, 40% TAC. Comparator: no testing and contact tracing
Pham et al. [24]<br>R_{eff}	Netherlands;<br>Outbreak from first cases in the context of a hospital, personal protective measures in place	ABM	(1) Baseline: health-care workers (HCWs) using personal protective equipment (PPE) in COVID wards when attending to patients; (2) (1) + HCWs working in specific wards without Ward change; (3) PPE in all wards: (1) + PPE in non-COVID wards. (4) Regular HCW screening: (1) + All HCWs are tested either with (4a) a test with perfect sensitivity (periodicity: 3 days) or a test with time-varying sensitivity, (4b) with a 3-day periodicity or (4c) with a 7-day periodicity. If testing positive, HCWs self-isolate for 7 days. (5) HCW contact tracing: (1) + If a HCW has symptomatic infection, all contacts of index case are traced and tested; (5a) 7-day contact tracing with perfect sensitivity (contacts instantaneously tested on day of symptom onset of index case): (5b) 7-day contact tracing with time-varying imperfect sensitivity (if contacts with index case was more than 5 days back, contacts tested on day of index case symptom onset; otherwise, contacts tested 5 days after contact); (5c) 2-day contact tracing with time-varying imperfect sensitivity, contacts tested

Annex 3.2 (cont.). Overview of mathematical modelling studies.

Authors and Outcomes assessed	Setting (country, regions); Period of the epidemic modelled	Type of modelling approach	Intervention(s) and comparator(s)
Pollmann et al. [42] R_{eff}, number of healthy people in quarantine, number of infected, reduction in R	Not stated; Ongoing epidemic in a context other than 2020 lockdown reopening, social distancing	ABM	(1) instantaneous digital contact tracing, (2) digital contact tracing in combination with random testing and social distancing, and (3) delay in contact tracing and secondary contact tracing. Comparator: no intervention
Quilty et al. [45] Transmission potential	UK; Epidemic from first cases in context other than 2020 lockdown reopening	ABM	(1) Different quarantine duration for index case contacts (0/3/5/7/10 days) and testing at the end of the quarantine (contacts identified = 100%), delay in tracing 3 days); (2) Daily testing for index case contacts with lateral flow tests at 1/3/5/7/10/14 days and quarantine if display symptoms or test positive (contacts identified = 100%), delay in tracing 3 days); (3) Intervention (1) with a lower contact tracing delay of 1.5/0 days; Intervention (2) with a lower contact tracing delay of 1.5/0 days. Comparator: baseline scenario with a 14-day quarantine period and no testing, 3 days contact tracing delay, 50% adherence to quarantine and 67% adherence to self-isolation
Reich et al. 1 [49] Share of the population infected, R_{eff}	Unclear; Epidemic from first cases in context other than 2020 lockdown reopening, social distancing	ABM	Combinations of (1) Mass testing of the population: random testing, (2) Testing only symptomatic individuals: a fraction of infected is tested daily, (3) Testing symptomatic individuals, tracing their contacts + testing them with varying probability (10%-100%) + isolating of those testing positive. Comparator: No intervention
Reich 2 [50] fraction of the population ever infected	Not stated; Epidemic from first cases in context other than 2020 lockdown reopening, social distancing	ABM	(1) Contact tracing with varying levels of: symptom onset to test time of index case (0.2, 0.5, 1, 2, 3.33 days), time from test of index case to test and quarantine of contacts (0, 1, 2, 3, 4, 8 days), and share of contacts traced (0, 0.5, 0.8, 1). All interventions modelled with some social distancing measures. The interventions are compared to each other
Scott et al. [25] Number of cases	Victoria (AUS); 2020 lockdown reopening	ABM	(1) Policy relaxations, (2) digital contact tracing with app with some policy relaxations (reopening of pubs, removing work from home directives), (3) physical distancing in social venues and (4) collection of patron identification records by venues. The interventions are compared to each other and to no intervention
Shamil et al. [26] Number and proportion of infected, R_{eff}	Ford County, Kansas and New York City (USA); Epidemic from first cases in a context other than contact tracing	ABM	(1) Digital contact tracing with all smartphone users using a contact tracing app, (2) lockdown and (3) Interventions (1) and (2). Comparator: no intervention
Stuart et al. [27] New infections	New South Wales (AUS); 2020 lockdown reopening, social distancing	ABM	(1) Testing, (2) community-based contact tracing and (3) mask usage at different levels in a context of low transmission (reopening with some NPIs in place) and high mobility. Specifically: 4 combinations of testing symptomatic and asymptomatic contacts (50%, 65%, 80% and 90%), 5 combinations of contact tracing (0%, 25%, 50%, 75% and 100%), 4 combinations of mask uptake (0%, 35%, 50% and 100%). Comparator: no intervention
Tatapudi et al. [28] Number of infections, cases, hospitalized and deaths	One county in Florida (USA); 2020 lockdown reopening, social distancing	ABM	(1) Stay-at home orders (from March 17, 2020), (2) Partial reopening (from May 18, 2020) including 50% of workplace areas, limiting large events to 25% capacity and limitations to gatherings of 10 or more people, (3) Additional reopening including 50%-75% of workplace capacity and large events with 50% capacity and limitations to gathering of 10 or more people (from June 5, 2020), (4) Intervention (3) with mandatory use of face masks (from June 25, 2020), (5) Intervention (4) + contact tracing with 50% target (from June 30, 2020). The interventions are compared to each other

Annex 3.2 (cont.). Overview of mathematical modelling studies.

Authors and Outcomes assessed	Setting (country, regions); Period of the epidemic modelled	Type of modelling approach	Intervention(s) and comparator(s)
Thompson et al. [29]<br>Number of cases, deaths	Luxembourg;<br>2020 lockdown reopening, no social distancing	ABM	(1) Testing (large scale and by prescription one day after case has symptoms), (2) contact tracing with limited capacity (100 cases per day get all their contacts traced), (3) quarantining (default period of 14 days), (4) curfew, (5) location closure, (6) Mask wearing and (7) vaccination. For testing and contact tracing, four scenarios are generated: baseline (agents behave as normal); low (daily testing capacity of 100 + 800 invitations for large scale testing + 100 cases have their contacts traced)/ medium (daily testing capacity of 5000 + 4000 invitations for large scale testing + contact tracing capacity of 300)/ high (10000 cases tested + 8000 invitations for large scale testing + contact tracing capacity of 500). Comparator: no intervention
Tuomisto et al. [46]<br>Active infections, cumulative cases, hospitalizations, deaths, recovered, R_{eff}	Helsinki (Finland);<br>2020 lockdown reopening, social distancing	ABM	(1) mitigation strategy - variable mobility restrictions, “lazy testing” (testing of cases with severe symptoms only), building hospital beds; (2) suppression strategy - variable mobility restrictions, aggressive testing (testing all individuals with respiratory symptoms and also of their contacts) with rising accuracy of contact tracing over time (30%, 40%, 50% and 60%), building hospital beds. The interventions are compared to each other and to no intervention
Wallentin et al. [47]<br>Number of active cases over time	Salzburg (Austria);<br>2020 lockdown reopening, social distancing	ABM	(1) Extended lockdown, (2) stepwise relaxation of lockdown as undertaken by government (small shops open on April 14 2020, all shops open on May 1 2020, small events are allowed on July 1) : (3) intervention (2) + traditional contact tracing under low (13%) / medium (26%) / high (39%) success in contact tracing, and (4) stepwise relaxation of lockdown with monitoring and adaptive response (modelled as backtracking on relaxation if active cases exceed capacity levels of intensive care units). The interventions are compared to each other
Wells et al. [30]<br>Epidemic size	Four counties in Wales (UK);<br>Epidemic from first cases in a context other than 2020 lockdown reopening	ABM	(1) Contact tracing and isolation of symptomatic and (2) any infected individual with varying levels of quarantine efficacy (0%-90%). Comparator: no intervention
Willem et al. [31]<br>Hospital admissions	Belgium;<br>2020 lockdown reopening, social distancing	ABM	(1) Testing + contact tracing with variable success (90% for household members, 50% for non-household members, with the assumption that 50% of symptomatic cases have their contacts traced).A large number of exit scenarios are generated varying location-specific* social mixing and including "household bubbles"**. Interventions are compared to a baseline lockdown exit scenario with limited social mixing and to each other.<br><br>*business to business, community, school social contact tracing.<br>mixing and including "household bubbles"<br>**repetitive leisure contacts in extended household settings
Zafarnejad et al. [48]<br>Number of cases, transmission risk	Not stated;<br>Reopening education institutions after 2020 lockdown	ABM	(1) Changes in class schedule and duration, (2) social distancing, (3) ventilation and air filtration, and (4) surveillance testing (10% of individuals) and contact tracing (25%, 50%, 75%, 100%) in a classroom. Comparator: no intervention
Allali et al. [52]<br>R_{eff}, number of asymptomatic cases detected in contacts of index case to contain the epidemic	Guadeloupe;<br>Epidemic from first cases in context other than 2020 lockdown reopening, social distancing	SBP	(1) Testing, (2) tracing and (3) quarantining with different delays in testing and quarantining in the context of a reduction of R due to the presence of other non-pharmaceutical interventions. Comparator: no intervention

<u>Annex 3.2 (cont.). Overview of mathematical modelling studies.</u>

Authors and Outcomes assessed	Setting (country, regions); Period of the epidemic modelled	Type of modelling approach	Intervention(s) and comparator(s)
Bradshaw et al. 1 [53]<br>R_{eff}, % outbreaks controlled	USA;<br>Epidemic from first cases, no social distancing	SBP	(1) Manual forward contact tracing (2-day and 6-day window); (2) Manual bidirectional contact tracing (2-day and 6-day window); (3) Digital contact tracing with different levels of app uptake; (4) Manual forward contact tracing + digital contact tracing with low and high app uptake (5) Manual forward and bidirectional (2-day and 6-day window) contact tracing + digital contact tracing with low (53% of cases) and high (80% of cases) app uptake. All for different levels of R expressing different levels of implementation of non-pharmaceutical interventions. Interventions are compared to each other and to no intervention
Bradshaw et al. 2 [54]<br>R_{eff}	Unclear;<br>Epidemic from first cases, social distancing	SBP	(1) Identification and isolation of cases, (2) bidirectional contact tracing (2-day and 6-day window) in the context of other non-pharmaceutical interventions. Comparator: Intervention (1)
Brook et al. [55]<br>Daily case counts	UC Berkeley community (USA);<br>Reopening after 2020 lockdown in educational institutions	SBP	(1) Group size limits, (2) symptom isolation, (3) surveillance testing of all students (weekly, semi-weekly, two-weekly); (4) contact tracing (90% of infectious contacts traced and isolated within one day of case isolation) to (2) and (3) Interventions are compared to each other
Davis et al. [56]<br>Probability of a large outbreak, R_{eff}	UK;<br>Epidemic from first cases, social distancing	SBP	Testing and tracing with (1) no delay in tracing, 1-day delay in tracing and 2-day delay in tracing, (2) poor/ good/ enhanced compliance with test and trace, and (3) variable levels of tracing coverage in the context of other pharmaceutical interventions. Combinations of the interventions are compared to each other
Endo et al. [67]<br>Proportion of generation 3 cases averted	Unclear;<br>Epidemic from first cases, social distancing	SBP	(1) Forward tracing only, (2) Forward + backward tracing. The interventions are compared to each other
Filonets et al. [57]<br>R_{eff}, probability of outbreak control	Taiwan;<br>Epidemic from first cases, social distancing	SBP	(1) Medical or non-medical mask wearing, (2) case isolation, (3) restrictions on gatherings and (4) contact tracing. The interventions are compared to each other
Firth et al. [58]<br>Cumulative number of infections, cases after 70 days	UK;<br>Epidemic from first cases, no social distancing (main analysis)	SBP	(1) no control, where no individuals are isolated or quarantined; (2) case isolation, where individuals isolate upon symptom onset after a delay period; (3) primary contact tracing with quarantine, where individuals isolate upon symptom onset (after a delay) and traced contacts are quarantined upon their infector’s symptom onset (also after a delay); and (4) secondary contact tracing, as in scenario (3) but including contacts of contacts. Comparator: Intervention (1)
Fyles et al. [59]<br>Growth rate of the epidemic.	UK;<br>2020 lockdown reopening, social distancing	SBP	(1) Contact tracing with household structure: (1a) household-level tracing, (1b) or individual-level tracing and (1c) without household structure (individual-level tracing) ; (2) contact tracing with household structure: (2a) upon symptom onset/ symptom report of index case / untraced contacts and perfect adherence to quarantine, (2b) with a positive test, (2c) upon symptom onset/ symptom report of index case / untraced contacts and imperfect adherence to quarantine; (3) Individual-level backward contact tracing with different time windows, with and without recall decay and with (50%) and without (0%) contact tracing app uptake. Comparator: self-isolation without contact tracing
Hellewell et al. [60]<br>% of outbreaks controlled	Unclear;<br>Epidemic from first cases, with and without social distancing	SBP	(1) Isolation of symptomatic individuals with a delay and contact tracing (primary contacts isolated immediately after having symptoms, secondary contacts isolated with a delay). Several scenarios for intervention (1) were modelled: short (=3.43 days) and long (=8.09 days) delay from symptom onset to isolation; Variable rate of coverage in contact tracing (0%-100%). Comparator: no intervention
Huamani et al. [61]<br>Outbreaks controlled	Peru;<br>2020 lockdown reopening, no social distancing	SBP	(1) Isolation of symptomatic individuals and contact tracing.<br>Two scenarios are modelled: Pre-quarantine (before national lockdown) and Post-quarantine (after lift of lockdown). Comparator: no intervention

Annex 3.2 (cont.). Overview of mathematical modelling studies.

Authors and Outcomes assessed	Setting (country, regions); Period of the epidemic modelled	Type of modelling approach	Intervention(s) and comparator(s)
Huang et al. [68]<br>Cumulative cases	Singapore;<br>Epidemic from first cases, social distancing	SBP	(1) Contact tracing, (2) self-awareness to exposure, and (3) Intervention (1) + Intervention (2) during three stages: Stage 1 was 23 Jan-16 March 2020, Stage 2 was 17 March-7 April 2020 (restrictions on non-essential traveling to countries affected by COVID-19 and 14-day quarantine for returning travelers introduced), Stage 3 was from 7 April (stringent social distancing measures introduced). Strategies are compared to each other and to no intervention
James et al. [62]<br>R_{eff}	New Zealand;<br>Epidemic from first cases, social distancing	SBP	(1) Isolation and contact tracing with different levels of coverage, varying levels of delay to contact tracing and varying efficacy of isolation and quarantine. Different levels of Intervention 1 are compared to each other and to no intervention
Kinoshita et al. [63]<br>R_{eff}, probability of a major epidemic	Unspecified;<br>Epidemic from first cases, with and without social distancing	SBP	(1) Contact tracing and isolation given different levels of effectiveness of contact tracing, and changes in relative infectiousness of asymptomatic individuals compared to symptomatic. Comparator: no contact tracing and isolation
Kretzschmar et al. 1 [64]<br>R_{eff}	The Netherlands;<br>Epidemic from first cases, some social distancing	SBP	(1) Physical distancing; (2) testing and isolation of index cases; (3) Conventional contact tracing with different tracing delays; (4) App-based tracing with different levels of app coverage. Strategies (3) and (4) are compared to (1) and (2)
Kretzschmar et al. 2 [65]<br>R_{eff}, critical tracing coverage, exponential growth rate, doubling time	The Netherlands;<br>Epidemic from first cases, with/ without social distancing	SBP	(1) Multiple scenarios of contact tracing coverage, contact tracing delays. Comparator: Baseline scenario = when a case is diagnosed, he/she is isolated, 100% traced, 0 days to find and isolate both household and non-household contacts.
Ng et al. [66]<br>R_{eff}, probability of outbreak extinction	Taiwan;<br>Epidemic from first cases, social distancing	SBP	(1) Case-based intervention (case isolation, contact tracing and quarantine for 7/14 days regardless of symptoms), (2) population-based intervention (social distancing and mask use) and (3) Intervention (1) + Intervention (2). Comparator: no case isolation, contact tracing and quarantine
Peak et al. [69]<br>R_{eff}	Not stated;<br>Epidemic from first cases, social distancing	SBP	(1) Active monitoring of contacts and (2) Individual quarantine under two settings which vary in the probability of tracing an infected contact, contact tracing delay, reduction of infectiousness during quarantine for pre-symptomatic contacts, frequency of monitoring symptoms in pre-symptomatic contacts, reduction of infectiousness during isolation as follows: High feasibility setting (probability of tracing = 0.9, 0.5 days contact tracing delay, 75% reduction in infectiousness for contacts in quarantine, frequency of monitoring symptoms in pre-symptomatic contacts = 0.5 days, isolation reduces infectiousness by 90%) and Low feasibility setting (0.5, 2 days, 0.25, 2 days, 0.5). Comparator: no contact tracing
Plank et al. [51]<br>R_{eff}, mean outbreak size after 30 days, probability of elimination	New Zealand;<br>Epidemic from first cases, no social distancing	SBP	(1) Manual contact tracing (home contacts traced instantly, work contacts with probability 0.5, school contacts with probability 0.8, casual contacts with probability 0.25), (2) digital contact tracing (all contacts traced with probability 0.9), and (3) combination of digital and manual contact tracing, including recursive tracing of second order contacts (i.e. contacts of contacts). Comparator: case isolation with no contact tracing.
Cencetti et al. [70]<br>Rate of the number of newly infected individuals	Not stated;<br>Epidemic from first cases, social distancing	Other	(1) Digital contact tracing with different levels of app adoption and quarantine of identified contacts under different levels of quarantine efficiency. Comparator: no digital contact tracing

Annex 3.2 (cont.). Overview of mathematical modelling studies.

Authors and Outcomes assessed	Setting (country, regions); Period of the epidemic modelled	Type of modelling approach	Intervention(s) and comparator(s)
Ferretti et al. [72] Growth rate of the epidemic as a function of % success rate in isolating cases and quarantining contacts	Not stated; Epidemic from first cases, no social distancing	Other	(1) Isolation and manual contact tracing (3 days frm start of symptoms to isolation and contact quarantine), (2) isolation and manual contact tracing (2 days from start of symptoms to isolation and contact quarantine), isolation and manual contact tracing (1 day from start of symptoms to isolation and contact quarantine), isolation and digital contact tracing (instantaneous contact tracing). All interventions are modelled for a range of success rates in isolation and contact tracing. Comparator: no contact tracing
Grassly et al. [73] R_{eff}	UK; Epidemic from first cases, no social distancing	Other	(1) Self-isolation of symptomatic individuals; (2) weekly PCR testing of health care workers and high-risk groups with different assumptions on test sensitivity; (3) testing symptomatic individuals with varying testing rates, tracing their contacts with a varying coverage + quarantining contacts of individuals who test positive. Delays in testing and tracing are also explored. Comparator: Intervention (1)
Kim et al. [78] Percentage of the population that needs to be enrolled (f_e^{min}) for automated contact tracing to be successful	Unclear; Epidemic from first cases, with and without contact tracing	Other	(1) Automated contact tracing with a variable fraction of the infected population which is confirmed positive by testing, a variable fraction of app users who confirmed they are diagnosed positive, and variable probabilities of disease transmission. Comparator: no intervention
Kucharski et al. [71] R_{eff}	UK; Epidemic from first cases, with and without social distancing	Other	Different combinations of self-isolation, contact tracing and physical distancing: (1) Self-isolation within home; (2) Self-isolation outside home; (3) Self-isolation + household quarantine; (4) Intervention (3) + school or work contact tracing (100% traceable); (5) Intervention (3) + manual contact tracing of acquaintances (53% traceable); (6) Intervention (3) + manual contact tracing of all contacts (100% traceable); (7) Intervention (3) + digital (app) contact tracing (53% traceable); (8) Intervention (3) + manual contact tracing of acquaintances + digital contact tracing (90% traceable at work, 79% traceable in school, 52% traceable other, 53% traceable with app); (9) Intervention (3) + manual contact tracing of acquaintances (90% traceable at work, 79% traceable in school, 52% traceable other) + limit of 4 daily contacts; (10) Intervention (3) + manual contact tracing of acquaintances (same as above) + app-based tracing (same as above) + limit of 4 daily contacts; and (11) Mass-testing of 5% of the population per week. Comparator: no intervention
Kuzdeuov et al. [75] Number of infected, quarantined, deaths and isolated	Lecco (Lombardy, Italy); Epidemic from first cases, social distancing	Other	(1) Random testing and isolation with different levels, (2) digital contact tracing with different levels of coverage, (3) Intervention (1)+(2). Comparator: no intervention.
Moran et al. [76] Endemic equilibrium	England; Ongoing epidemic, social distancing	Other	(1) Find, track, trace and isolate (FTTI) with 5% policy efficacy; (2) FTTI with 20% policy efficacy; FTTI with 40% policy efficacy; (4) FTTI policy with 60% policy efficacy. All interventions modelled with some social distancing Comparator: FTTI with 0% policy efficacy.
Sanche et al. [77] R_{eff}	China; Epidemic from first cases, social distancing	Other	(1) Contact tracing, (2) quarantine and (3) social distancing / Comparator: no intervention
Worden et al. [78] R_{eff}	San Francisco (USA); Epidemic from first cases, social distancing	Other	(1) Contact tracing; (2) Mask wearing; (3) interventions (1) + (2). Comparator: Shelter in place order

Annex 4. Study type, characteristics and sources of data.

Annex 4.1. Study design, modelling approach/ statistical analysis, and sources of data for the empirical studies.

**Authors;**<br>**Setting;**<br>**Study design (Longitudinal/ cross-sectional)**	**Modelling approach/ statistical analysis**	**Sources of data**
Haug et al. [4]<br>79 territories / 46 countries worldwide<br>Longitudinal study	Four techniques: (1) case-control analysis; (2) Step function lasso regression; (3) RF regression; (4) Transformer modelling	NPI data: Complexity Science Hub COVID-19 Control Strategies List; CoronaNet COVID-19 Government Response Event Dataset ; WHOGlobal Dataset of Public Health and Social Measures dataset; Novel Coronavirus COVID-19 (2019-nCoV)<br>Cases data: Data Repository by Johns Hopkins CSSE
Hong et al. [9]<br>108 countries<br>Cross-sectional study	Multiple linear regression	NPI data and cases: Oxford COVID-19 Government Response Tracker
Kendall et al. [2]<br>Isle of Wight (UK)<br>Longitudinal study	Bayesian estimation of R (hospital cases) and ML estimation of R (community cases) in Isle of Wight and also estimation of average R in comparison area using synthetic controls	NPI data: NHS Test and Trace programme<br>Incidence data: Upper Tier Local Health Authorities in England
Leffler et al. [7]<br>200 countries worldwide<br>Longitudinal study	Multivariate linear regression	NPI data: Oxford COVID-19 Government Response Trracker<br>Mortality data: Worldometer
Liu et al. [5]<br>130 countries worldwide<br>Longitudinal study	Panel regression – fixed effects model with individual intercept	NPI data and cases: Oxford COVID-19 Government Response Tracker
Papadopoulos et al. [10]<br>137 countries worldwide<br>Longitudinal study	Univariate / multivariate linear regression	NPI data and cases: Oxford COVID-19 Government Response Tracker
Pozo-Martin et al. [3]<br>37 OECD countries<br>Longitudinal study	Panel regression with mixed effects model	NPI data and cases: Oxford COVID-19 Government Response Tracker
Vecino-Ortiz et al. [6]<br>32 departments and 5 districts in Colombia<br>Longitudinal study	Sequential regression with fixed effects model	NPI and incidence data: Colombian National Institute of Health<br>Mortality data: departments and districts
Wibbens et al. [8]<br>Growth rate in cases<br>40 territories: 17 countries and 23 US states<br>Longitudinal study	Bayesian modelling	NPI data and cases: Oxford COVID-19 Government Response Tracker
Wymant et al. [1]<br>England and Wales<br>Longitudinal study	Matched neighbour regression and modelling	NPI data: NHS COVID-19 app<br>Data on number of cases: https:/coronavirus.data.gov.uk
Malheiro et al. [11]<br>Eastern Porto (Portugal)<br>Longitudinal study	Chi-squared tests, Mann-Whitney test	NPI data and cases: surveillance data from Porto authorities
Park et al. [12]<br>Seoul, South Korea<br>Longitudinal study	Deterministic analysis of time to event data	NPI data and cases: surveillance data from Seoul authorities

Annex 4.2. Representation of infection and disease, social interactions, and model parameters of the mathematical modelling studies.

**Authors; Setting; Modelling approach**	**Social interactions: Network layers, Contact structure, and Data sources**	**Infection and disease: Distinguishes symptomatic-asymptomatic carriers? Distinguishes levels of severity of symptoms?**	**Model parameters (distribution if applicable)**
Abueg et al. [13]<br>Washington state (USA);<br>ABM	Households, workplaces, schools and random networks;<br>Age-specific vector;<br>U.S. Census of Population and Housing and ACS Public Use Microdata Sample.	Yes;<br>Yes (mild, severe)	Incubation time = 5.42 days<br>% asymptomatic Age-dependent
Aleta et al. [14]<br>Boston (USA);<br>ABM	Households, workplaces and community level, and schools;<br>Layer-specific contact network;<br>U.S. census (Boston data)	Yes;<br>Yes (mild, hospitalization, ICU)	Latent period = 3 and 5 days<br>% asymptomatic = 25%<br>R_0 = 2.5-3.0
Barthe et al. [32]<br>Tübingen (Germany);<br>ABM	Unique network layer;<br>Age-specific vector;<br>Unclear	Yes;<br>No	% asymptomatic = 40%
Bhattacharyya et al. [33]<br>Bengaluru (India);<br>ABM	Multiple wards;<br>Unclear;<br>Unclear	No;<br>Yes (mild, severe and critical)	Incubation time = 5 days<br>R_0 = 2.8
Bicher et al. [15]<br>Austria;<br>ABM	Households, workplaces, schools and leisure layer;<br>Layer-specific contact network;<br>Austrian national statistics office ‘‘Statistics Austria’’ and POLYMOD.	Yes;<br>Yes (mild, severe, critical and hospitalization states)	Incubation time ~ 5 days<br>Latent period = 3 days
Colomer et al. [34]<br>Spain;<br>ABM	Unique network layer;<br>Unclear;<br>Instituto Nacional de Estadística (INE)	Yes;<br>Yes (distinguises severity depending on hospitalization)	Incubation time = 5.1 days ±10%
Eilersen et al. [35]<br>Denmark;<br>ABM	Households, workplaces, friends, public;<br>Layer-specific contact network;<br>Survey of mixing patterns (Mossong et al []) and Danish family composition statistics	Yes;<br>No	Incubation time = 5 days<br>Serial interval = 4.6 days<br>R_0 = 3
Fiore et al. [43]<br>New York metropolitan area (USA), Southeast Italy and midlands (UK);<br>ABM	Unique network layer;<br>Unclear;<br>Unclear	Yes;<br>Yes (mild, severe)	Incubation time is Normally distributed (mean = 5.5 days, sd=2; skewness=0.6)<br>% asymptomatic = 20%-30%<br>R_0 ~3.05, ~2.18, and ~1.31
Geffen et al. [44]<br>Unspecified;<br>ABM	Unique network layer;<br>Unclear;<br>Unclear	Yes;<br>No	Unclear
Goldberg et al. [36]<br>Not stated;<br>ABM	Unique network layer;<br>Random network called a Geometric Inhomogenous Random Graph (GIRG);<br>Unclear	Yes;<br>Yes (mild and severe)	Unclear
Goldenbogen et al. [37]<br>Various municipalities;<br>ABM	Households, workplaces, schools and public places;<br>Age-specific contact matrix;<br>German census	No;<br>No	Unclear
Gressman et al. [17]<br>USA;<br>ABM	Students and instructors (University environment);<br>Contact network using Poisson point processes;<br>University of Pennsylvania.	Yes;<br>Unclear	Incubation time = 5.2 (discretized Gamma distribution)<br>R_0 = 2.8-5.8

Annex 4.2 (cont.). Representation of infection and disease, social interactions, and model parameters of the mathematical modelling studies.

**Authors; Setting; Modelling approach**	**Social interactions: Network layers, Contact structure, and Data sources**	**Infection and disease: Distinguishes symptomatic-asymptomatic carriers? Distinguishes levels of severity of symptoms?**	**Model parameters (distribution if applicable)**
Hill et al. [18]<br>A University in the UK<br>ABM	Households, study groups, organised societies and sports clubs, social<br>Layer-specific contact networks<br>Survey of contact frequencies, UK University records	Yes;<br>No	Incubation time is Erlang distributed (mean = 6.82 days)<br>% asymptomatic – follows a Uniform distribution (mean = 65%)<br>Uncontrolled R (early outbreak stage) = 3-4
Hill et al. [19]<br>A worker population in the UK;<br>ABM	Households, workplaces, social, other;<br>Layer-specific contact network;<br>UK census, Office of National Statistics data, survey of contact frequencies	Yes;<br>No	Incubation time is Erlang distributed (mean = 6.82 days)<br>% asymptomatic – follows a Uniform distribution (mean = 65%)<br>Uncontrolled R (early outbreak stage) = 2-4
Kerr et al. [20]<br>Seattle (USA);<br>ABM	Households, workplaces, schools, community and long-term care facilities;<br>Age-specific contact matrix by layer and random network for community layer;<br>United States Census Bureau and **Prem et al.**	Yes;<br>Yes (mild, severe, critical)	Time from infectious to symptoms is Lognormally distributed (mean=1.1, sd=0.9)<br>Latent period is Lognormally distributed (mean=4.5, sd=1.5)<br>% asymptomatic is age-dependent<br>R_0 = 2.0, 2.5 and 3.1
Li et al. [16]<br>USA;<br>ABM	Households, workplaces, schools and community;<br>Age-specific contact matrix by layer and random network for community layer;<br>Unclear	Yes;<br>Yes (mild, severe (hospitalization) and critical (ICU))	see Kerr et al.
Low et al. [38]<br>Masiphumele township, Cape Town (SA);<br>ABM	Households, workplaces, schools, public transport and blocks (collections of households);<br>Unclear;<br>Unclear	No;<br>No	Unclear
Luo et al. [21]<br>Wuhan (China);<br>ABM	Households, small communities, large communities;<br>Layer-specific contact network;<br>China Community Network, 2020 China Statistical Yearbook, Wuhan micro-neighbourhood small programme, 2019 China National Population Sampling Survey	Yes;<br>Yes	Incubation time is normally distributed (mean = 5.11, sd = 2.5)<br>% asymptomatic = 27.3%<br>R0 = 3.11
Moon et al. [39]<br>Manhattan, New York (USA);<br>ABM	Contact-layer and tracing-layer;<br>Age-specific vector;<br>U.S. census (Manhattan)	Yes;<br>No	R_0 = 0.55
Moreno López et al. [22]<br>France;<br>ABM	Households, workplaces, schools, community and transport;<br>Age-specific contact matrix and ratio of contacts by layer;<br>National Institute of Statistics and Economic Studies (INSEE) censuses	Yes;<br>Yes (mild, moderate, severe)	Incubation time = 5.2 days<br>Latent period = 2.9 days<br>% asymptomatic is age-dependent<br>R_0 = 1.7, 2.6 y 3.1
Mukherjee et al. [40]<br>University of Illinois, (USA);<br>ABM	Unique network layer;<br>Number of contacts for each agent and time;<br>Several universities	Yes;<br>No	R_0 = 1.82

Annex 4.2 (cont.). Representation of infection and disease, social interactions, and model parameters of the mathematical modelling studies.

Authors;<br>Setting;<br>Modelling approach	Social interactions:<br>Network layers,<br>Contact structure, and<br>Data sources	Infection and disease:<br>Distinguishes symptomatic-asymptomatic carriers?<br>Distinguishes levels of severity of symptoms?	Model parameters<br>(distribution if applicable)
Ng et al. [23]<br>Canada;<br>ABM	Households, workplaces, schools and mixed age venues;<br>Age-specific contact matrix by layer;<br>Canadian census and **Prem et al.**	Yes;<br>Yes (mild and severe)	Latent period = 3.68 days (PERT distribution)<br>% asymptomatic ~38%<br>R_0 = 2.0-3.0
Panovska-Griffiths et al. [41]<br>UK;<br>ABM	Households, workplaces, schools and community;<br>Age-specific contact matrix by layer;<br>UK Government and **Prem et al.**	Yes;<br>Yes (mild, severe and critical)	see Kerr et al.
Pham et al. [24]<br>Netherlands;<br>ABM	Hospital population and community;<br>Different contact rates between doctors-nurses-patients;<br>University Medical Center Utrecht (UMCU)	Yes;<br>Yes (moderate and severe)	Incubation time is Gamma distributed (mean=5.510 days, sd=2.284)<br>% asymptomatic = 20% (patients) and 31% (health-care workers)<br>R_0 is dependent on wild-type or new virus variant
Pollmann et al. [42]<br>Unclear;<br>ABM	Unique network layer;<br>Social contact structure divided into 1) homogeneous population and 2) social graph population;<br>Unclear	Yes;<br>Unclear	Incubation time ~7 days (Gamma dsitribution)<br>% asymptomatic = 40%-95%<br>R_0 = 2.0, 3.0 and 4.0
Quilty et al. [45]<br>UK;<br>ABM	Unique network layer;<br>Unclear;<br>Unclear	Yes;<br>No	Incubation time is Lognormally distributed (logmean=1.63, logsd=0.50), i.e., mean 5.78 days<br>% asymptomatic is Beta distributed (alpha=51, beta=115), i.e., mean=31%
Reich et al. 1 [49]<br>Unclear;<br>ABM	Unique network layer;<br>Unclear;<br>Unclear	No;<br>No	Incubation time is Gamma distributed (mean = 5.10 days, sd=4.4)
Reich 2 [50]<br>Not stated;<br>ABM	Unique network layer;<br>Unclear;<br>Unclear	Yes;<br>No	Incubation time is Gamma distributed (mean=5.10, sd=4.4)<br>% asymptomatic = 40%
Scott et al. [25]<br>Victoria (AUS);<br>ABM	Households, workplaces, several community spaces and activities;<br>Age-specific contact matrix by layer and random networks for some community spaces;<br>Australian Bureau of Statistics and Prem et al.	Yes;<br>Yes (mild, severe and critical)	see Kerr et al.
Shamil et al. [26]<br>Ford County, Kansas, New York City (USA);<br>ABM	Set of groups: F = Stay home, T = Commute, W = Work or attend school, E = Attend event and H = Stay at the hospital;<br>Agents are associated with groups (depending on the person’s task) for interaction at every time unit (i.e., hour);<br>Ford county census and New York City data	Yes;<br>Unclear	Unclear
Stuart et al. [27]<br>New South Wales (AUS);<br>ABM	Households, workplaces, schools, static and dynamic community;<br>Age-specific contact matrix by layer and random networks for some community spaces;<br>New South Wales census	Yes;<br>Yes (mild, severe and critical)	see Kerr et al.

Annex 4.2 (cont.). Representation of infection and disease, social interactions, and model parameters of the mathematical modelling studies.

**Authors;<br>Setting;<br>Modelling approach**	**Social interactions:<br>Network layers,<br>Contact structure, and<br>Data sources**	**Infection and disease:<br>Distinguishes symptomatic-asymptomatic carriers?<br>Distinguishes levels of severity of symptoms?**	**Model parameters<br>(distribution if applicable)**
Tatapudi et al. [28]<br>Miami-Dade county, Florida (USA);<br>ABM	Households, workplaces, schools and community;<br>Unclear;<br>United States Census Bureau (Miami)	Yes;<br>Yes (distinguises severity depending on hospitalization)	Incubation time = 5.5 days<br>Latent period = 3 days<br>% asymptomatic = 35%
Thompson et al. [29]<br>Luxembourg;<br>ABM	Households, primary and secondary schools, restaurants, shops, hospitals; and types of working location categorized by sector;<br>Unclear;<br>STATEC	Yes;<br>Yes (distinguises severity depending on hospitalization)	Incubation time = 6.05 days (Exponentially distributed)<br>% asymptomatic is age-dependent<br>R_0 = 2.45
Tuomisto et al. [46]<br>Helsinki, Finland;<br>ABM	Unique network layer;<br>Age-specific contact matrix;<br>Unclear	Yes;<br>Yes (mild, severe, critical)	Incubation time = 5.10 days (Gamma distributed)<br>% asymptomatic = 50%
Wallentin et al. [47]<br>Austria (Salzburg);<br>ABM	Unique network layer;<br>Unclear;<br>Salzburg data	No;<br>No	R_0 = 2.5-3.5
Wells et al. [34]<br>Four counties in Wales (UK)<br>ABM	Urban populations, rural populations;<br>Layer-specific contact network;<br>UK Census, survey of contact frequencies (Danon et al [])	Yes;<br>No	Incubation time: 4-6 days<br>% asymptomatic: 10%-70%<br>Transmission rate: 0.001-0.3
Willem et al. [31]<br>Belgium;<br>ABM	Households, workplaces, schools and community;<br>Age-specific contact matrix by layer and weekdays/weekends;<br>Belgian census	Yes;<br>Unclear	Incubation time is Lognormally distributed (logmean=1.43, logsd=0.66), i.e., mean = 5.20 days<br>% asymptomatic is age-dependent<br>R_0 = 3.41
Zafarnejad et al. [48]<br>Not stated;<br>ABM	Unique network layer;<br>Unclear;<br>Unclear	Yes;<br>Yes (mild, severe)	Incubation time is Normally distributed (mean=5.75 days, sd=5.75/3)<br>Latent period is Normally distributed (mean=2, sd=2/3)<br>% asymptomatic = 40%
Allali et al. [52]<br>Guadeloupe;<br>SBP	Unique network layer;<br>Unclear;<br>Regional Health Agency	Yes;<br>Yes (mild, severe; critical)	% asymptomatic = 20%<br>R_0 = 1.5, 2.0, 3.0 and 4.0
Bradshaw et al. 1 [53]<br>USA;<br>SBP	Unique network layer;<br>Each case generates a number of new cases drawn from a negative binomial distribution;<br>Unclear	Yes;<br>No	Incubation time median 5.5±2.1 days<br>Generation time follows Skew-Normal distribution<br>% asymptomatic = 40%-55%<br>R_0 = 2.5
Bradshaw et al. 2 [54]<br>Unclear;<br>SBP	Unique network layer;<br>Each case generates a number of new cases drawn from a negative binomial distribution;<br>Unclear	Yes;<br>No	Incubation time median 6.0±2.1 days<br>Generation time follows Skew-Normal distribution<br>% asymptomatic = 40%<br>R_0 = 1-2

Annex 4.2 (cont.). Representation of infection and disease, social interactions, and model parameters of the mathematical modelling studies.

Authors;<br>Setting;<br>Modelling approach	Social interactions:<br>Network layers,<br>Contact structure, and<br>Data sources	Infection and disease:<br>Distinguishes symptomatic-asymptomatic carriers?<br>Distinguishes levels of severity of symptoms?	Model parameters<br>(distribution if applicable)
Brook et al. [55]<br>UC Berkeley community;<br>SBP	Unique network layer;<br>Each case generates a number of new cases drawn from a negative binomial distribution;<br>Unclear	Yes;<br>No	Incubation time = 2-4 days<br>Generation time follows Weibull distribution (mean = 5.05, sd = 1.94)<br>% asymptomatic = 32%<br>R_0 = 2.5
Davis et al. [56]<br>Unclear;<br>SBP	Unique network layer;<br>Each case generates a number of new cases drawn from a negative binomial distribution;<br>Unclear	Yes;<br>No	Incubation time is lognormally distributed (logmean=1.43 days, logsd=0.66), i.e., mean = 5.20 days<br>% asymptomatic = 31%<br>R = 1.3 and 1.5
Endo et al. [67]<br>Unclear;<br>SBP	Unique network layer;<br>Unclear;<br>Unclear	No;<br>No	R_0 = 0.8, 1.2 and 2.5
Filonets et al. [57]<br>Taiwan;<br>SBP	Unique network layer;<br>Each case generates a number of new cases drawn from a negative binomial distribution;<br>Unclear	Yes;<br>No	Incubation time follows a Weibull distribution (mean=5.75 days, sd=2.63)<br>Generation time is Skew-Normally distributed (mean=incubation time, sd=2)<br>% asymptomatic = 15% (symptomatic-produced) and 50% (asymptomatic-produced)<br>R_0 = 1.5, 2.5 and 3.5
Firth et al. [58]<br>UK;<br>SBP	Unique network layer;<br>Unclear;<br>Unclear	Yes;<br>No	Incubation time = 5.8 days (Weibull-distributed)<br>Generation time follows Skew-Normal distribution (mean=incubation time, sd=2)<br>% asymptomatic = 20% and 40%<br>R_0 = 2.0, 2.8 and 3.5
Fyles et al. [59]<br>UK;<br>SBP	Unique network layer;<br>Unclear;<br>Unclear	Yes;<br>No	Incubation time is Gamma-distributed (mean=4.84 days, sd=2.79)<br>Generation time is Weibull-distributed (mean=5.0 days, sd=1.92)
Hellewell et al. [60]<br>Unclear;<br>SBP	Unique network layer;<br>Each case generates a number of new cases drawn from a negative binomial distribution;<br>Unclear	Yes;<br>No	Incubation time is Weibull-distributed (mean=5.8 days, sd=2.6)<br>Generation time is Skew-Normally distributed (mean=incubation time, sd=2)<br>% asymptomatic = 0% and 10%<br>R_0 = 1.5, 2.5 and 3.5
Huamani et al. [61]<br>Peru;<br>SBP	Unique network layer;<br>Unclear;<br>Unclear	Yes;<br>No	Incubation time 5.8±2.6 days<br>% asymptomatic = 18% and 40%<br>R_0 = 2.7 and 3.5 (pre-quarantine),<br>R_0 = 1.5, 2.0 and 2.7 (post-quarantine)

Annex 4.2 (cont.). Representation of infection and disease, social interactions, and model parameters of the mathematical modelling studies.

Authors;<br>Setting;<br>Modelling approach	Social interactions:<br>Network layers,<br>Contact structure, and<br>Data sources	Infection and disease:<br>Distinguishes symptomatic-asymptomatic carriers?<br>Distinguishes levels of severity of symptoms?	Model parameters<br>(distribution if applicable)
Huang et al. [68]<br>Singapore;<br>SBP	Unique network layer;<br>Each case generates a number of new cases drawn from a negative binomial distribution;<br>Unclear	No;<br>No	Incubation time = 7.1 days (Weibull-distributed)<br>Serial interval = 3.23 days (Skew-Normal-distributed)<br>R_0 = 1.2 - 2.5
James et al. [62]<br>New Zealand;<br>SBP	Unique network layer;<br>Unclear;<br>Unclear	Yes;<br>No	Incubation time is Gamma-distributed (mean=5.5 days, sd=2.3)<br>Generation time is Weibull-distributed (mean=5.05, sd = 1.94)<br>% asymptomatic = 80%, 33% and 20%<br>R_0 = 2.0 to 4.0
Kinoshita et al. [63]<br>Unclear;<br>SBP	Unique network layer;<br>Unclear;<br>Unclear	Yes;<br>No	% asymptomatic = 40% (10%-70%)<br>R_0 = 1.5 to 3.5
Kretzschmar et al. 1 [64]<br>The Netherlands;<br>SBP	Unique network layer;<br>Unclear;<br>Unclear	Yes;<br>No	Incubation time based on Backer et al.<br>Serial interval based on Nishiura et al.<br>% asymptomatic = 20%<br>R_0 = 2.5
Kretzschmar et al. 2 [65]<br>The Netherlands;<br>SBP	Unique network layer;<br>Unclear;<br>Unclear	Yes;<br>No	Incubation time is Lognormally-distributed (logmean=1.434065, logsd=0.6612), i.e., mean = 5.22 days<br>% asymptomatic = 20% (0%-100%)<br>R_0 = 1.0-3.0
Ng et al. [66]<br>Taiwan;<br>SBP	Unique network layer;<br>Unclear;<br>Unclear	Yes;<br>No	Incubation time, mean = 5.5 days, sd=3.26<br>Serial interval = 5.86 days<br>% asymptomatic = 40% (20%-60%)<br>R_0 2.5 = 2.0 to 3.0 days
Peak et al. [69]<br>Not stated;<br>SBP	Unique network layer;<br>Each case generates a number of new cases drawn from a negative binomial distribution;<br>Unclear	Yes;<br>No	Incubation time = 5.2 days<br>Serial interval = 4.8 and 7.5 days<br>R_0 = 2.2 days
Plank et al. [51]<br>New Zealand;<br>SBP	Households, workplaces, schools and casual;<br>Age-specific contact matrix by layer;<br>New Zealand and **Prem et al.**	Yes;<br>No	Incubation time is Gamma-distributed (mean=5.5 days, sd=2.3)<br>Generation time is Weibull-distributed (mean=5.05, sd=1.94)<br>% asymptomatic is age-dependent<br>R_0 = 2.6

Annex 4.2 (cont.). Representation of infection and disease, social interactions, and model parameters of the mathematical modelling studies.

**Authors;<br>Setting;<br>Modelling approach**	**Social interactions:<br>Network layers,<br>Contact structure, and<br>Data sources**	**Infection and disease:<br>Distinguishes symptomatic-asymptomatic carriers?<br>Distinguishes levels of severity of symptoms?**	**Model parameters<br>(distribution if applicable)**
Cencetti et al. [70]<br>Unclear;<br>Other	Several social environments (a university campus, a high school and a workplace);<br>Unclear;<br>Copenhagen Networks Study (CNS) data set	Yes;<br>No	% asymptomatic = 20%-40%<br>R_0 = 1.2, 1.5 and 2.0
Ferretti et al. [72]<br>Not specified;<br>Other	Unique network layer;<br>Unclear;<br>Unclear	Yes;<br>No	Incubation time is Lognormally-distributed (logmean=1.644, logsd=0.363), i.e., mean=5.53<br>Generation time is Weibull-distributed (mean=5.05, sd=1.94)<br>% asymptomatic = 40%<br>R_0 = 2.0
Grassly et al. [73]<br>UK;<br>Other	Unique network layer;<br>Unclear;<br>Unclear	Yes;<br>No	Incubation time = 5.5 days (Gamma-distributed)<br>Generation time = 6 days (Weibull-distributed)<br>% asymptomatic = 33%
Kim et al. [78]<br>Unclear;<br>Other	Unique network layer;<br>Every individual described by a circle;<br>Unclear	Yes;<br>Yes	Unclear
Kucharski et al. [71]<br>UK;<br>Other	Household, workplaces, schools and other;<br>Randomly generation of contacts from the marginal distributions of daily contacts by age group;<br>BBC Pandemic dataset	Yes;<br>No	Incubation time = 5 days<br>Serial interval = 6.5 days<br>% asymptomatic = 30% (children), 70% (adults)<br>R_0 = 2.6
Kuzdeuov et al. [75]<br>Lecco (Lombardy);<br>Other	Unique network layer;<br>Particle simulation where contact occurs when the distance between the particles becomes less than the contact threshold;<br>Unclear	Yes;<br>Yes (mild, severe)	Unclear
Moran et al. [76]<br>England;<br>Other	Unique network layer;<br>Distinguishes effective number of contacts in home and work;<br>Unclear	Yes;<br>No	Incubation time = 5 days
Sanche et al. [74]<br>China;<br>Other	Unique network layer;<br>Unclear;<br>Unclear	Yes;<br>No	Incubation time = 4.2 days<br>Serial interval = 6-9 days<br>R_0 = 5.7
Worden et al. [77]<br>San Francisco (USA);<br>Other	Unique network layer;<br>Unclear;<br>Unclear	No;<br>No	Incubation time is Lognormally-distributed (mean=5.6, sd=4.2)

Annex 5. Expanded results: mathematical modelling studies.

Authors; Setting; Type of modelling approach	Intervention(s) and Comparator	Results as applicable to contact tracing	Quality score/ comments (studies with score <8)
Abueg et al. [13] Washington state (USA): King, Pierce, and Snohomish counties; ABM	(1) Digital contact tracing of 1st order contacts - 90% probability of quarantine; (2) Manual contact tracing – likelihood of tracing household/ workplace/ random contacts = 100% / 80% / 5% (3) Intervention (1) combined with Intervention (2). Comparator: no intervention	Results are presented graphically.<br><br>(1) Digital contact tracing: An app with 75% (15%) adoption reduces the total number of infections by 56–73% (3.9–5.8%), 73–79% (8.1–9.6%), and 67–81% (6.3–11.8%) and deaths by 52–70% (2.2–6.6%), 69–78% (11.2–11.3%), and 63–78% (8.2–15.0%) compared to the default scenario of only self-isolation due to symptoms for King, Pierce, and Snohomish counties, respectively.<br><br>(2) Manual contact tracing: With ideal staffing levels (15 per 100,000) manual contact tracing significantly affects the epidemic. However, with available working capacity the impact of the epidemic is much reduced.<br><br>(3) Digital + Manual contact tracing (fully staffed): 22%-37% app adoption is required to compensate a reopening of 10-20%.	9
Aleta et al. [14] Boston (USA); ABM	(1) After 8 weeks of lockdown, reopening of all work and community places except mass-gathering environments (4 weeks) followed by full reopening; (2) Intervention (1) + testing of symptomatic individuals (50% successfully diagnosed), tracing (varying fraction of non-household contacts) and quarantining (household and non-household contacts). Strategies are compared to the unmitigated scenario in which no interventions are implemented	Results are presented graphically.<br><br>Number of hospitalizations (ICU) in the unmitigated scenario = 4.57 (2.56); with Intervention (1), no tracing = 2.70 (1.58); with Intervention (2), 30% detection and 60% tracing = 0.35 (0.22) = 87% reduction in hospitalisations; with Intervention (2), 50% detection and 40% tracing = 0.29 (0.15) = 86% reduction in hospitalisations	9
Bicher et al. [15] Austria; ABM	(1) Tracing of household members; (2) Tracing of co-workers; (3) Interventions (1) + (2); (4) Intervention (3) + tracing of individual contacts (50% accuracy); (5) Intervention (3) + tracing of individual contacts (75% accuracy). The interventions are compared to no contact tracing and to each other assuming other measures are in place that achieve high (74.58%) infectivity reduction	Results are presented graphically.<br><br>With 74.58% infectivity reduction due to other policies, Cumulative infected agents (May 15–Nov 15) are 371,709 (no contact tracing), 220,046 (Household tracing), 244,092 (Workplace tracing), 165,167 (Combined tracing), 141,822 (50% individual tracing) and 119,826 (75% individual tracing). That is a reduction in the cumulative infected agents (May 15–Nov 15) of 40.80% (1), 34.33% (2), 55.57% (3), 61.85% (4) and 67.76% (5), with respect to no contact tracing.<br><br>Compared to no contact tracing, the contribution to the required infectivity reduction for disease steady state is 1.62% (household tracing), 1.05% (workplace tracing), 3.11% (combined tracing), 4.12% (50% individual tracing), and 5.18% (75% individual contact tracing)	9

Annex 5 (cont.). Expanded results: mathematical modelling studies.

Authors; Setting; Type of modelling approach	Intervention(s) and Comparator	Results as applicable to contact tracing	Quality score/ comments (studies with score <8)
Li et al. [16]<br>USA;<br>ABM	(1) Mask wearing, (2) school closing, (3) work closing, (4) social distancing, (5) testing, (6) contact tracing and (7) quarantining. Interventions are compared to each other	(1) Masks + remote school/work had the highest impact accounted for 66.64% of variance in new cases<br><br>(2) Masks + remote school/work + the ability to trace contacts of tested individuals accounted for 13.76% of the variance in new cases<br><br>(3) The ability to trace contacts of tested individuals accounted for 8.95% of the variance in new cases<br><br>(4) Masks + remote school/work + whether contact tracing starts without waiting for test results accounted for 2.80% of the variance.	9
Gressman et al. [17]<br>USA;<br>ABM	(1) "Standard intervention" = Daily random testing of 3% of students and of contacts identified the previous day + quarantining all students who test positive and all symptomatic with 100% success rate + contact tracing of all positive tested and all symptomatic + transitioning all classes with 30 or more students to online-only interaction + mask wearing (50% reduction of transmission) + 5% immunity at start of term; (2) Excluding testing from "standard intervention", 3) excluding contact tracing. Interventions are compared to each other and to no intervention	Results are presented graphically<br><br>In the absence of any intervention, all scenarios end with effectively all susceptible community members developing COVID-19 by the end of the semester.<br><br>"Standard intervention" median cumulative infections = 43.<br><br>Excluding random testing/ contact tracing, median cumulative infections are 50/ 47, respectively. For contact tracing, this is a reduction of 8.5% of infections	9
Hill et al. [18]<br>A University in UK<br>ABM	(1) Contact tracing with varying levels of trace and quarantine efficacy. Comparator: no intervention	Results are presented graphically<br><br>Intervention (1): With 40%/60%/80%/100% trace and quarantine efficacy, the proportion of students infected is reduced by 16%/29%/48%/68% compared to no intervention	9
Hill et al. [19]<br>A worker population in the UK;<br>ABM	(1) Contact tracing with varying levels of trace and quarantine efficacy. Comparator: no intervention	Results are presented graphically<br><br>Intervention (1): With 100% trace and quarantine efficacy, the size of the outbreak was reduced by 50% and the peak in infectious prevalence was reduced by 75%	9
Kerr et al. [20]<br>Seattle (USA);<br>ABM	(1) Testing, tracing, quarantining with a variable range of quarantine effectiveness, routine testing probability, swab-to-result delay, contact tracing probability, quarantine testing probability, and contact tracing delay. Intervention (1) is implemented under 3 scenarios: 60%, 80% and 100% mobility. Comparator: Baseline scenario is a high mobility (100%) scenario with high testing (6000 tests per day) and high tracing (70% of all household and workplace contacts within two days).	Results are presented graphically<br><br>Results relevant to contact tracing: Each quarantined person who fully isolates is estimated to avert 1.2 subsequent infections over a 90-day period, a one-day delay in tracing contacts is estimated to result in roughly 2 additional infections for every index case whose contacts are traced. While quarantine testing had the smallest overall impact on the attack rate, an additional 0.4 infections are still estimated to be averted for every index case whose contacts are tested. With a delay to tracing of 2 days, for a return to 100% mobility, high testing (2.7 tests per 1000 people per day) and tracing (probability of contact tracing higher than 70%) are required to maintain epidemic control in the setting of this study.	9

<u>Annex 5 (cont.). Expanded results: mathematical modelling studies.</u>

**Authors; Setting; Type of modelling approach**	**Intervention(s) and Comparator**	**Results as applicable to contact tracing**	**Quality score/ comments (studies with score <8)**
Luo et al. [21]<br>Wuhan (China)<br>ABM	(1) Contact tracing of symptomatic and asymptomatic cases with variable rate of coverage of asymptomatic cases (0%-100%); (2) Different delays in implementing contact tracing (30,50,70,90 days after the start of the epidemic). Comparator: no contact tracing	Results are presented graphically<br><br>Intervention (1): At day 100, with 0%/25%/50%/75%/100% of asymptomatic cases traced there is a reduction in the infection density per 10000 population of 64.9%/69.1%/75.7%/80.2%/87.7%.<br><br>Intervention (2): With 75% of asymptomatic cases traced, delays in the implementation of contact tracing has little effect on the density of infection	9
Moreno López et al. [22]<br>France;<br>ABM	(1) Combined testing (50% of symptomatic individuals), self-isolation and isolation of household contacts + digital contact tracing with different levels of app adoption. Comparator: no intervention, self-isolation and isolation of household contacts	Results are presented graphically.<br><br>With R_0 = 2.6, self-isolation and isolation of household contacts reduces peak incidence by 27% (0% app adoption); adding digital contact tracing with 20% app adoption, relative reduction in peak incidence = 35%; with 60% app adoption, relative reduction in peak incidence = 66%. There is an additional mitigation effect (30%-144%) provided by contact tracing compared to household isolation only.<br><br>With R_0 = 1.7, adding digital contact tracing with 20% app adoption, relative reduction in peak incidence = 45% (additional mitigation effect of 25%), with 60% app adoption, relative reduction in peak incidence = 89% (additional mitigation effect of 147%).<br><br>32% app adoption would bring the epidemic to manageable levels.	9
Ng et al. [23]<br>Canada;<br>ABM	Different combinations of case detection and isolation, contact tracing and quarantine, physical distancing and community closures. Relevant for contact tracing are: (1) Contact tracing and quarantine (20% of cases detected, 50% of contacts of these cases traced and quarantined), (2) Enhanced case detection and contact tracing (50% of cases detected and isolated with 50% of household members co-isolating + 100% of these cases traced and quarantined), (3) Enhanced case detection and contact tracing with physical distancing interventions (20% reduction in the contact rate). The interventions are compared to each other and to no intervention.	Results presented graphically<br><br>Results relevant to contact tracing:<br><br>Contact tracing (success rate of 50%) and quarantine, The attack rate with no intervention was 64.6% (95% CrI 63.9%-65%). With intervention (1), the total attack rate =was 62.5% (95% CI 62.0%–63.3%), a small reduction.<br>No intervention = 64.6% (95% CrI 63.9%–65.0%)<br><br>Intervention (2) attenuated the total attack rate markedly to 0.4% (95% CrI 0.03%–23.5%).<br><br>Intervention (3) reduced , the total attack rate was to 0.2% (95% CrI 0.03%–1.7%).	9

Annex 5 (cont.). Expanded results: mathematical modelling studies.

Authors; Setting; Type of modelling approach	Intervention(s) and Comparator	Results as applicable to contact tracing	Quality score/ comments (studies with score <8)
Pham et al. [24] Netherlands; ABM	(1) Baseline: health-care workers (HCWs) using personal protective equipment (PPE) in COVID wards when attending to patients; (2) (1) + HCWs working in specific wards without Ward change; (3) PPE in all wards: (1) + PPE in non-COVID wards. (4) Regular HCW screening: (1) + All HCWs are tested either with (4a) a test with perfect sensitivity (periodicity: 3 days) or a test with time-varying sensitivity, (4b) with a 3-day periodicity or (4c) with a 7-day periodicity. If testing positive, HCWs self-isolate for 7 days. (5) HCW contact tracing: (1) + If a HCW has symptomatic infection, all contacts of index case are traced and tested, with contacts wearing PPE at all times; (5a) Contacts up to 2 days before symptom onset are traced with time-varying test sensitivity; (5b) contacts up to 7 days before symptom onset are traced and tested on the day of symptom onset (5b1) or on the day of symptom onset if the contact was more than 5 days ago, otherwise the test was performed on day 5 after the contact (5b2) . Comparator: (1)	Results presented graphically<br><br>R_{eff} baseline scenario: 0.65<br><br>Intervention (5a) obtained R_{eff} = 0.41 (reduction of 37%); Intervention (5b1) obtained R_{eff} = 0.44 (reduction of 32%); Intervention (5b2) obtained R_{eff} = 0.39 (reduction of 40%)<br><br>Contact tracing was highly effective in limiting nosocomial transmissions in our model, especially when traced contacts are tested at least five days after their exposure and precautionary measures are undertaken in the meantime. If traced HCWs are immediately tested, self-isolated, and replaced by susceptible HCWs, this can lead to increased transmission	9
Scott et al. [25] Victoria (AUS); ABM	(1) Policy relaxations, (2) digital contact tracing with app with some policy relaxations (reopening of pubs, removing work from home directives), (3) physical distancing in social venues and (4) collection of patron identification records by venues. The interventions are compared to each other and to no intervention	Results are presented graphically<br><br>Contact tracing app coverage would need to be higher than 30% to reduce the population-level transmission risk after opening pubs and bars or removing work from home directives.<br><br>Opening pubs and bars, recording the identification of patrons would be effective if at least 60% of contacts were reliably traced.	9
Shamil et al. [26] Ford County, Kansas and New York City (USA); ABM	(1) Digital contact tracing with all smartphone users using a contact tracing app, (2) lockdown and (3) Interventions (1) + (2). Strategies are compared to no intervention and to each other	Results are presented graphically<br><br>Contact tracing with 60% of app users can lead to R_{eff} < 1 within three weeks of the start of the intervention. With 75% smartphone users, new infections are eliminated within 3 months.<br><br>Digital contact tracing in combination with lockdown has a higher impact. Introducing contact tracing over time – day 21/ day 27/ day 41 leads to 10.89%/ 14.63%/ 26.70% infections after 90 days.	9

Annex 5 (cont.). Expanded results: mathematical modelling studies.

Authors; Setting; Type of modelling approach	Intervention(s) and Comparator	Results as applicable to contact tracing	Quality score/ comments (studies with score <8)
Stuart et al. [27]<br>New South Wales (AUS);<br>ABM	(1) Testing, (2) community-based contact tracing and (3) mask usage at different levels in a context of low transmission (some non-pharmaceutical interventions in place) and high mobility. Specifically: 4 combinations of testing symptomatic and asymptomatic contacts (50%, 65%, 80% and 90%), 5 combinations of contact tracing (0%, 25%, 50%, 75% and 100%), 4 combinations of mask uptake (0%, 35%, 50% and 100%). Strategies compared to each other.	Results are presented graphically<br><br>All strategies with some contact tracing and high testing (90% of people with symptoms and 90% of asymptomatic contacts of confirmed cases) with high mask uptake achieve epidemic control (median ~180 total infections between October-December 2020) lead to a robustly controlled epidemic.<br><br>With very high testing rates (90% of people with symptoms, plus 90% of contacts of a confirmed case) and fast and effective contact tracing, epidemic control can be achieved without widespread mask usage.<br><br>With medium-lower testing rates of 50% or 65%, the marginal impact of both masks and contact tracing is considerable<br><br>With lower testing rates, mask use can play an important role in epidemic control.	9
Tatapudi et al. [28]<br>Miami-Dade county, Florida (USA);<br>ABM	(1) Stay-at home orders (from March 17, 2020), (2) Partial reopening (from May 18, 2020) including 50% of workplace areas, limiting large events to 25% capacity and limitations to gatherings of 10 or more people, (3) Additional reopening including 50%-75% of workplace capacity and large events with 50% capacity and limitations to gathering of 10 or more people (from June 5, 2020), (4) Intervention (3) with mandatory use of face masks (from June 25, 2020), (5) Intervention (4) + contact tracing with 50% target (from June 30, 2020). The interventions are compared to each other	Results are presented graphically<br><br>Intervention (5) compared with Intervention (4): Infections/cases/hospitalizations/deaths are respectively 581K and 1.74M / 247K and 714K / 35.2K and 120K / 8.8K and 29.7K. This is a reduction of 66%/ 65%/ 70%/ 70%<br><br>Intervention (1) leaves a large proportion of the population susceptible. Reopening should include a strong implementation of social distancing, mask wearing, contact tracing, testing, and isolation of infected	9
Thompson et al. [29]<br>Luxembourg;<br>ABM	(1) Testing (large scale and by prescription one day after case has symptoms), (2) contact tracing with limited capacity (100 cases per day get all their contacts traced), (3) quarantining (default period of 14 days), (4) curfew, (5) location closure, (6) Mask wearing and (7) vaccination. For testing and contact tracing, four scenarios are generated: baseline (agents behave as normal); low (daily testing capacity of 100 + 800 invitations for large scale testing + 100 cases have their contacts traced)/ medium (daily testing capacity of 5000 + 4000 invitations for large scale testing + contact tracing capacity of 300)/ high (10000 cases tested + 8000 invitations for large scale testing + contact tracing capacity of 500). Comparator: no intervention	Results are presented graphically<br><br>Medium / high levels of testing and contact tracing significantly reduce the total number of cases but not deaths.<br><br>Testing and tracing does not necessarily address specifically the needs of vulnerable individuals (such as those living in care homes).	9
Wells et al [30]<br>Four counties in Wales (UK)<br>ABM	(1) Contact tracing and isolation of symptomatic and (2) any infected individual with varying levels of quarantine efficacy (0%-90%). Comparator: no intervention	Results are presented graphically.<br><br>Intervention (2): With 47% or higher quarantine efficacy, epidemic size is reduced by at least 95%	9

Annex 5 (cont.). Expanded results: mathematical modelling studies.

**Authors; Setting; Type of modelling approach**	**Intervention(s) and Comparator**	**Results as applicable to contact tracing**	**Quality score/ comments (studies with score <8)**
Willem et al. [31]<br>Belgium;<br>ABM	(1) Testing + contact tracing with variable success (90% for household members, 50% for non-household members, with the assumption that 50% of symptomatic cases have their contacts traced). A large number of exit scenarios are generated varying location-specific* social mixing and including "household bubbles"**. Interventions are compared to a baseline lockdown exit scenario with limited social mixing and to each other.	Results are presented graphically<br><br>By varying the success rate of contact tracing per index case, the relative number of hospital admissions ranged from 35% to 60% of hospital admissions in the base case scenario without contact tracing.<br><br>Average reduction in hospital admissions of 22% in June and 58% in August with the contact tracing strategy in place with respect to baseline, assuming that 70% of the symptomatic cases are subjected to contact tracing and comply with home isolation. The combination of contact tracing and repetitive social mixing in household bubbles has the potential to reduce the average number of hospital admissions up to 87% by August. Tracing non-household contacts seems to have highest impact on hospital admissions.<br><br>Delays in isolating index cases are more important than delays in contact tracing.<br><br>Substantial impact of household bubbles on reducing hospital admissions.	9
Barthe et al. [32]<br>Tübingen (Germany);<br>ABM	(1) Implementation of PanCast, which uses bluetooth beacons strategically located to match IDs of contacts with IDs of positive-testing individuals + manual contact tracing – this results in bidirectional contact tracing, and (2) Digital contact tracing (smartphone-based, pairwise encounter-based) + manual contact tracing. Comparator: manual contact tracing	Results are presented graphically<br><br>Intervention (1): PanCast with 20% adoption, reduction in the number of infections = 10% compared to manual contact tracing.<br>Intervention (2): Digital contact tracing with 50% adoption, reduction in contact tracing = 10% compared to manual contact tracing.	8
Bhattacharyya et al [33]<br>Bengaluru (India);<br>ABM	(1) Lockdown, (2) Mild lockdown, (3) fixed duration lockdown, (4) Two-day week followed by strict lockdown, (5) Tracing and quarantining neighbours (and neighbours of) of infected patients, (6) Quarantining of city ward and reopening with different relaxation factors. Comparator: no intervention	Results are presented graphically<br>With respect to tracing and quarantining, policy (5) on its own reduces the percentage of recovered from 84.96% to 55.63% (about 30 points) and the number of deaths per million from 8702 to 5642 (i.e. by 3060); For (2) + (5) these values are 84.96% to 1.09% / 8702 to 103; For (6)+(5) 84.96% to 1.05%/ 8702 to 171; For (1)+(4)+(5) 84.96% to 0.35%/ 8702 to 34; for (1)+(5) 84.96% to 0.35%/ 8702 to 43 .	8

Annex 5 (cont.). Expanded results: mathematical modelling studies.

Authors; Setting; Type of modelling approach	Intervention(s) and Comparator	Results as applicable to contact tracing	Quality score/ comments (studies with score <8)
Colomer et al [34]<br>Spain;<br>ABM	(1) Varying levels of vaccination, 2) varying levels of contact tracing, 3) vaccination and varying levels of contact tracing with and without implementing other non-pharmaceutical interventions (social distancing + hand washing + mask wearing). Different levels of implementation of strategies (1)-(3) with and without other non-pharmaceutical interventions are compared to not tracing	Results are presented graphically<br><br>Intervention (2) without other pharmaceutical interventions (probability of transmission=0.1): Out of a population of 46 million, with 0% contact tracing the percentage of dead is 0.41. With 10%/20%/30%/40% contact tracing there is a reduction in percentage of dead of 48%/ 66%/ 76%/ 83%.<br><br>Intervention (2) with other pharmaceutical interventions (probability of transmission=0.05): Out of a population of 46 million, with 0% contact tracing the percentage of dead is 0.33%. With 10%/20%/30%/40% contact tracing there is a reduction in percentage of dead of 64%/ 76%/ 82%/ 82%<br><br>Intervention (3) with 19.56% vaccination and no pharmaceutical interventions (probability of transmission=0.1): Out of a population of 46 million, with 0% contact tracing the percentage of dead is 0.31%. With 10%/20%/30%/40% contact tracing there is a reduction in percentage of dead of 48%/ 65%/ 74%/ 74%.<br><br>Intervention (3) with 19.56% vaccination and pharmaceutical interventions (probability of transmission=0.05): Out of a population of 46 million, with 0% contact tracing the percentage of dead is 0.17%. With 10%/20%/30%/40% contact tracing there is a reduction in percentage of dead of 47%/ 65%/ 65%/ 71%.	8
Eilersen et al. [35]<br>Denmark;<br>ABM	(1) One-step tracing and quarantining – i.e. isolation of agents with positive test results, closure of their workplaces, identification and quarantining of their regular social contacts with different levels of testing and quarantine duration. Comparator: no intervention	Results are presented graphically<br><br>Intervention (1) in a context other than lockdown reopening: With 10% of infected being tested per day of illness and quarantine duration of 5 days, the peak number of infected is reduced by 60%. With higher levels of testing, the peak number of infected can be further reduced<br><br>Intervention (1) after lockdown reopening: With 20% of infected being tested per day of illness and quarantine duration of 5 days, a resurgence of the epidemic is prevented	8
Goldberg et al. [36]<br>Not stated;<br>ABM	Digital contact tracing with user referrals under four scenarios: (1) app users are a random subset of population, (2) randomly selected users recommend the app to one contact*, (3) randomly selected initial users recommend the app to its neighbours, and these to about 50% of contacts**, and (4) app users are individuals with highest number of connections. All users notify contacts when they have symptoms***. The interventions are compared to each other<br><br>*Basic recommendation<br>**Ring recommender<br>***Degree targeting<br>Comparator: interventions compared to each other	Results are presented graphically<br><br>Intervention (4) is most effective, followed closely by Intervention (3)<br><br>Compared to Intervention (1), the epidemic size curves decrease at a faster rate under Intervention (2) and Intervention (3).<br><br>Recommending the tracing app to acquaintances reduces the epidemic size and maximum hospital load decrease at a much higher rate than randomly selecting the same number of app users.	8

Annex 5 (cont.). Expanded results: mathematical modelling studies.

Authors;<br>Setting;<br>Type of modelling approach	Intervention(s) and Comparator	Results as applicable to contact tracing	Quality score/ comments (studies with score <8)
Goldenbogen et al. [37]<br>Various municipalities;<br>ABM	(1) co-location recursive contact tracing (identifying individuals spatio-temporal overlap), (2) contact-based tracing (identifying actual interactions). Comparator: The interventions are compared to each other and to no intervention	Results are presented graphically<br><br>Co-location digital contact tracing decreases the expected proportion of positive results per identified case (when tracing all contacts) by a factor of 20 (from 1000 to 50 tests)	8
Low et al. [38]<br>Masiphumele township, Cape Town (SA);<br>ABM	(1) No contact tracing with a test turnaround time of two days, (2) contact tracing with a test turnaround time of two days, and (3) contact tracing with a test turnaround time of eight days. Comparator: No contact tracing with a test turnaround time of two days	Intervention (2): Mean number of infections reduced by 25.5% compared to Intervention (1)<br><br>Intervention (3): Mean number of infections reduced by 7.5% compared to Intervention (1)<br><br>Every additional day added to the test turn-around time increased the total number of infections – with the greatest increase in infections between 4 and 5 days, and the smallest increase between 10 and 11 days.	8
Moon et al. [39]<br>Manhattan, New YorkKansas (USA);<br>ABM	(1) Varying levels of contact tracing, testing + isolation of infected contacts of a confirmed case, (2) Varying levels of contact tracing, testing + isolation of all contacts of a confirmed case. Comparator: no contact tracing	Results are presented graphically<br><br>For 25%-50%-75%-100% reopening, and levels of contact tracing of 20%/40%/60% intervention (1) and intervention (2) show a percentage reduction in cases of, respectively, 84.60%/91.67%/92.41% - 72.60%/97.26%/98.22% - 55.49%/85.16%/96.87% - 42.39%/72.28%/96.60% and 72.64%/86.90%/89.17% - 44.24%/85.00%/97.96% - 32.21%/58.76%/86.59% - 27.90%/49.40%/72.75%	8
Mukherjee et al. [40]<br>University of Illinois (USA);<br>ABM	Combinations of (1) random testing and (2) contact tracing (results of tests are available the same day). Interventions are compared to each other	Results are presented graphically<br><br>If bulk testing is set at 1000 individuals per day, a lowering contact tracing success from 90% to 80% diminishes from 0.753 to 0.712 (= 5.44% reduction) the average number of susceptible individuals<br><br>If bulk testing is set at 15000 individuals per day, lowering contact tracing success from 90% to 80% diminishes from 0.891 to 0.890 (=1% reduction) the average number of susceptible individuals<br><br>Contact tracing has a much larger impact when bulk testing capacity is small. Contact tracing on its own is not enough to contain infection spread	8
Panovska-Griffiths et al. [41]<br>UK;<br>ABM	(1) Full-time school reopening, 18% testing of symptomatic (TS), 68% tracing of all contacts (TAC); (2) Full-time school reopening, 75% TS, 68% TAC; (3) Full-time school reopening, 87% TC, 40% TAC; (4) Part-time school reopening, 18% TS, 68% TAC; (5) Part-time school reopening, 65% TS, 68% TAC; (6) Part-time school reopening, 75% TS, 40% TAC. Comparator: no testing and contact tracing	Results are presented graphically<br><br>If schools reopen full-time/ part-time with 68% contact tracing, 75%/ 65% of individuals would have to be tested and isolated to keep $R_{eff}<1$.<br><br>If schools reopen full-time/ part-time with 40% contact tracing, 87%/ 75% of individuals would have to be tested and isolated to keep $R_{eff}<1$	8

Annex 5 (cont.). Expanded results: mathematical modelling studies.

Authors; Setting; Type of modelling approach	Intervention(s) and Comparator	Results as applicable to contact tracing	Quality score/ comments (studies with score <8)
Pollmann et al. [42]<br>Unclear;<br>ABM	(1) instantaneous digital contact tracing, (2) digital contact tracing in combination with random testing and social distancing, and (3) delay in contact tracing and secondary contact tracing. Comparator: no intervention	Results are presented graphically<br><br>Under high transmission ($R_0 = 3$), digital contact tracing leads to $R_{eff} < 1$ only if the proportion of symptomatic detected is > 80% and app adoption is > 90%.<br><br>Under lower transmission ($R_0 = 2$), digital contact tracing leads to $R_{eff} < 1$ if proportion of asymptomatic detected is 80% and app adoption is > 70%.<br><br>In both cases, perfect contact tracing is required.<br><br>In combination with random testing and social distancing (contact rate 80% of that at full mobility), at least 60% of the population needs to use digital contact tracing to reduce infection rates below $R_{eff} < 1$.<br><br>For an app coverage of 75%, the impact of tracing delays and secondary contact tracing depends strongly on the incubation time.	8
Fiore et al. [43]<br>New York metropolitan area (USA), Southeast Italy and midlands UK;<br>ABM	(1) Contact tracing, testing and isolation if testing positive for different levels of testing efficacy and contact tracing coverage and testing (20%, 40%, 60%, 80%, and 100%). Different scenarios were modelled for disease incidence (equivalent to a daily growth in cases of 15%, 25% or 35%) to represent alternative physical distancing policies. Comparator: no intervention	Results presented graphically<br>High (>60%) contact tracing and testing efficacy, and a testing capacity between 0.7 and 6.8 per thousand in a context of low (15%) and medium (25%) transmission efficiency led to suppression of the virus.<br><br>Very high (>80%) contact tracing and testing efficacy, and a testing capacity between 3.6 and 9.1 per thousand in a context of high (35%) transmission efficiency led to suppression of the virus (R<1).<br><br>Low contact tracing and testing efficacy (20% and 40%) in a context of medium (25%) or high (35%) transmission efficiency (25% or 35%) could not contain exponential trend of the epidemic.	7<br>No multi-layer network, limited representation of model
Geffen et al. [44]<br>South Korea;<br>ABM	(1) Isolation of agents with positive test results (85% mean adherence per day); (2) isolation of agents with positive tests results (85% mean adherence per day) + 10% of their contacts are traced and isolated (85% mean adherence per day), (3) isolation of agents with positive results (85% adherence per day), and 30% of their contacts are traced and isolated (85% mean adherence per day), (4) perfect isolation of agents when they test positive and perfect tracing of first and secondary contacts, who are then also perfectly isolated. Comparator: no isolation, no contact tracing.	Intervention (1)/(2)/(3)/(4), compared to no isolation or contact tracing leads to a mean reduction of infections of 402 (6.9%) /1324 (23%) /2384 (41%)/4749 (82%).	7<br>No social mixing or multi-layer network

<u>Annex 5 (cont.). Expanded results: mathematical modelling studies.</u>

Authors; Setting; Type of modelling approach	Intervention(s) and Comparator	Results as applicable to contact tracing	Quality score/ comments (studies with score <8)
Quilty et al. [45] UK; ABM	(1) Different quarantine duration for index case contacts (0/3/5/7/10 days) and testing at the end of the quarantine (contact tracing coverage = 100%), delay in tracing 3 days); (2) Daily testing for index case contacts with lateral flow tests at 1/3/5/7/10/14 days and quarantine if display symptoms or test positive (contacts identified = 100%), delay in tracing 3 days); (3) Intervention (1) with a lower contact tracing delay of 1.5/0 days; Intervention (2) with a lower contact tracing delay of 1.5/0 days. Comparator: baseline scenario with a 14-day quarantine period and no testing, 3 days contact tracing delay, 50% adherence to quarantine and 67% adherence to self-isolation	Results are presented graphically<br><br>Interventions (1) and (2) with quarantine periods greater than zero days yield risk ratio of averting transmission potential greater than 1 compared to the baseline scenario. For example, reducing the contact tracing delay to 0 days the risk ratio of 7-day quarantine with no testing may exceed the effect of 14-day quarantine with 3-day test and trace delay	7<br>No social mixing or multi-layer network
Tuomisto et al. [46] Helsinki, Finland; ABM	(1) mitigation strategy - variable mobility restrictions, “lazy testing” (testing of cases with severe symptoms only), building hospital beds; (2) suppression strategy - variable mobility restrictions, aggressive testing (testing all individuals with respiratory symptoms and also of their contacts) with rising accuracy of contact tracing over time (30%, 40%, 50% and 60%), building hospital beds. The interventions are compared to each other and to no intervention	Results are presented graphically<br><br>Intervention (1) with variable 30–40% mobility reduction delays the peak of the epidemic but does not suppress the epidemic.<br><br>Intervention (2): reduction of the cumulative number of infected individuals from 820,000 to 80,000 and the number of deaths from 6,000 to 640 when compared with Intervention (1) during the first year of the epidemic.<br><br>Intervention (2) is equally effective as Intervention (1) but allowing for 20% lower mobility restrictions.<br><br>Adding contact tracing to testing: deaths reduced from 75% to 92%.	7<br>No social mixing or multi-layer network
Wallentin et al. [47] Salzburg (Austria); ABM	(1) Extended lockdown, (2) stepwise relaxation of lockdown as undertaken by government (small shops open on April 14 2020, all shops open on May 1 2020, small events are allowed on July 1) : (3) intervention (2) + traditional contact tracing under low (13%) / medium (26%) / high (39%) success in contact tracing, and (4) stepwise relaxation of lockdown with monitoring and adaptive response (modelled as backtracking on relaxation if active cases exceed capacity levels of intensive care units). The interventions are compared to each other	Results are presented graphically.<br><br>Results relevant to contact tracing:<br><br>Intervention (3) with 26% success in contact tracing allows to reopen to a level of contact rates of 80% relative to those of before the pandemic.<br><br>Intervention (3) with 39% of contacts successfully traced has the potential of helping eradicate the virus.	7<br>No multi-layer network, no distinction between categories of infectiousness

<u>Annex 5 (cont.). Expanded results: mathematical modelling studies.</u>

Authors; Setting; Type of modelling approach	Intervention(s) and Comparator	Results as applicable to contact tracing	Quality score/ comments (studies with score <8)
Zafarnejad et al [48]<br>Not stated;<br>ABM	(1) Changes in class schedule and duration, (2) social distancing, (3) ventilation and air filtration, and (4) surveillance testing (10% of individuals) and contact tracing (25%, 50%, 75%, 100%) in a classroom. Comparator: no intervention	Results are presented graphically.<br><br>The decrease in infection risk is higher than 70% when switching from the lowest level of contact tracing possible to the highest level of contact tracing posible	7<br>No social mixing or multi-layer network
Reich et al. 1 [49]<br>Unclear;<br>ABM	Combinations of (1) Mass testing of the population: random testing, (2) Testing only symptomatic individuals: a fraction of infected is tested daily, (3) Testing symptomatic individuals, tracing their contacts + testing them with varying probability (10%-100%) + isolating of those testing positive. Comparator: No intervention	Results presented graphically.<br><br>With a 2-day delay in detecting an infected individual, tracing all contacts who test positive achieves epidemic containment (3% of population eventually infected). This is not the case for low contact tracing coverage (e.g. tracing 10% of contacts leads to 24% of the population infected).<br><br>For combined interventions to reduce R_{eff} below 1, the following is required:<br><br>Mass testing with contact tracing - Without social distancing, test more than 10% daily, trace 100% of contacts; with social distancing, test 10% daily and trace 50% of contacts.<br><br>Testing the symptomatic with contact tracing – Without social distancing, detect infected in 1 day, trace 50% of contacts; with social distancing, detect infected in 2 days, trace 50% of contacts.	6<br>No social mixing or multi-layer network, no distinction between categories of infectiousness
Reich 2 [50]<br>Not stated;<br>ABM	Three components of contact tracing for containment: (1) symptom onset to test time (0.2, 0.5, 1, 2, 3.33 days), (2) time from test of case to test of contacts (0, 1, 2, 3, 4, 8 days), and (3) share of contacts traced (0, 0.5, 0.8, 1). All options modelled with some social distancing. Comparator: strategies are compared to each other	Results presented graphically<br><br>Reducing the time to test and quarantine contacts (i.e. reducing tracing delays) reduces the share of the population infected, particularly if time between symptom onset and testing of the index case is reduced to one day. For a time between symptom onset and testing of the index case of 3.33 days, the higher the coverage of contacts, the higher the impact of reducing time to test and quarantine of contacts.	6<br>No social mixing or multi-layer network, parameters not fully described
Plank et al. [51]<br>New Zealand;<br>SBP	(1) Manual contact tracing (home contacts traced instantly, work contacts with probability 0.5, school contacts with probability 0.8, casual contacts with probability 0.25); (2) digital contact tracing (all contacts traced with probability 0.9), (3) combination of digital and manual contact tracing, including recursive tracing of second order contacts (i.e. contacts of contacts) and. Comparator: case isolation with no contact tracing.	Results are presented graphically<br><br>Intervention (1) with moderately (50%) effective quarantine of pre-symptomatic or subclinical individuals: R_{eff} reduction = from 2.4 to 1.56.<br><br>Intervention (2) digital contact tracing without manual contact tracing with high uptake rates (80%) R_{eff} reduction = from 2.4 to 1.46.<br><br>Intervention (3) with 50%/ 80% effective quarantine of pre-symptomatic or subclinical individuals: R_{eff} reduction = from 2.4 to 1.24 / 1.12. Adding recursive contact tracing reduced R_{eff} to 1.06	9

Annex 5 (cont.). Expanded results: mathematical modelling studies.

**Authors; Setting; Type of modelling approach**	**Intervention(s) and Comparator**	**Results as applicable to contact tracing**	**Quality score/ comments (studies with score <8)**
Allali et al. [52] Guadeloupe; SBP	(1) Testing, (2) tracing and (3) quarantining with different delays in testing and quarantining. Comparator: no intervention	Results are presented graphically<br><br>Slow-down solution where the number of cases and deaths is small (example: R_0=2, delay between testing and isolating is 2 days, 3 asymptomatic are contacted per symptomatic): ~200 people per day should be isolated.	7 No social mixing or multi-layer network
Bradshaw et al. 1 [53] USA; SBP	(1) Manual forward contact tracing (2-day and 6-day window); (2) Manual bidirectional contact tracing (2-day and 6-day window); (3) Digital contact tracing with different levels of app uptake; (4) Manual forward contact tracing + digital contact tracing with low and high app uptake (5) Manual forward and bidirectional (2-day window) contact tracing + digital contact tracing with low (53% of cases) and high (80% of cases) app uptake. All for different levels of R expressing different levels of implementation of non-pharmaceutical interventions. Interventions are compared to each other and to no intervention	Results are presented graphically<br><br>Intervention (1) with a 2-day/ 6-day tracing window led to an R reduction of 24%/ 24%<br><br>Intervention (2) with a 2-day/ 6-day tracing window led to a further R reduction (compared to (1)) of 24%/ 42%<br><br>Intervention (3) with 100% app uptake: R<1<br><br>Intervention (5) with low/ high app uptake led to further R reduction (compared to (1)) of 21% / 42%	7 No social mixing or multi-layer network
Bradshaw et al. 2 [54] Unclear; SBP	(1) Identification and isolation of cases, (2) bidirectional contact tracing (2-day and 6-day window) in the context of other non-pharmaceutical interventions. Comparator: Intervention (1)	Results are presented graphically<br><br>Intervention (2) with a 2-day tracing window and 70% contact tracing with 50% compliance with isolation led to the same reduction in R (10%) than Intervention (2) with a 6-day window and 50% contact tracing with 50% compliance with isolation.	7 No social mixing or multi-layer network
Brook et al. [55] UC Berkeley community; SBP	(1) Group size limits, (2) symptom isolation, (3) surveillance testing of all students (weekly, semi-weekly, two-weekly); (4) Adding contact tracing (90% of infectious contacts traced and isolated within one day of case isolation) to (1)-(3). Interventions are compared to each other	Results presented graphically<br><br>Adding contact tracing to semi-weekly test (time to results is 10 days) reduces cases by up to 17-fold	7 No social mixing or multi-layer network
Davis et al. [56] Unclear; SBP	Testing and tracing with (1) no delay in tracing, 1-day delay in tracing and 2-day delay in tracing, (2) poor/ good/ enhanced compliance with test and trace, and (3) variable levels of tracing coverage in the context of other pharmaceutical interventions. Combinations of the interventions are compared to each other	Results are presented graphically<br><br>With good compliance, fast testing and high contact tracing coverage, there is a potential reduction in R of 10-15%	7 No social mixing or multi-layer network

Annex 5 (cont.). Expanded results: mathematical modelling studies.

**Authors; Setting; Type of modelling approach**	**Intervention(s) and Comparator**	**Results as applicable to contact tracing**	**Quality score/ comments (studies with score <8)**
Filonets et al. [57]<br>Taiwan;<br>SBP	(1) Medical or non-medical mask wearing, (2) case isolation, (3) restrictions on gatherings and (4) contact tracing. The interventions are compared to each other	Results presented graphically<br><br>With 100% contact tracing and 90% use of non-medical masks and restrictions on gatherings, with higher transmission (R=2.5), There is a 90% probability that R will reach 1<br>With 60% contact tracing and 50% use of medical masks and restrictions on gatherings, with lower transmission (R=1.5), R <1	7<br>No social mixing or multi-layer network
Firth et al. [58]<br>UK;<br>SBP	(1) no control, where no individuals are isolated or quarantined; (2) case isolation, where individuals isolate upon symptom onset after a delay period; (3) primary contact tracing with quarantine, where individuals isolate upon symptom onset (after a delay) and traced contacts are quarantined upon their infector's symptom onset (also after a delay); and (4) secondary contact tracing, as in scenario (3) but including contacts of contacts. Comparator: Intervention (1)	Results presented graphically<br><br>Intervention (3): reduction in infections = 36%<br><br>Intervention (4): reduction in infections = 78%	7<br>No social mixing or multi-layer network
Fyles et al. [59]<br>UK;<br>SBP	(1) Contact tracing with household structure: (1a) household-level tracing, (1b) or individual-level tracing and (1c) without household structure (individual-level tracing) ; (2) contact tracing with household structure: (2a) upon symptom onset/ symptom report of index case / untraced contacts and perfect adherence to quarantine, (2b) with a positive test, (2c) upon symptom onset/ symptom report of index case / untraced contacts and imperfect adherence to quarantine; (3) Individual-level backward contact tracing with different time windows, with and without recall decay and with (50%) and without (0%) contact tracing app uptake. Comparator: self-isolation without contact tracing	Results presented graphically<br><br>Contact tracing with household structure and physical distancing 50%: R<1<br><br>Backward contact tracing at individual level with digital contact tracing (50% app adoption) reduces the daily growth rate in COVID-19 cases unless recall is reduced	7<br>No social mixing or multi-layer network

Annex 5 (cont.). Expanded results: mathematical modelling studies.

Authors; Setting; Type of modelling approach	Intervention(s) and Comparator	Results as applicable to contact tracing	Quality score/ comments (studies with score <8)
Hellewell et al. [60]<br>Unclear;<br>SBP	(1) Isolation of symptomatic individuals with a delay and contact tracing (primary contacts isolated immediately after having symptoms, secondary contacts isolated with a delay). Several scenarios for intervention (1) were modelled: short (=3.43 days) and long (=8.09 days) delay from symptom onset to isolation; Variable rate of coverage in contact tracing (0%-100%). Comparator: no intervention	Results are presented graphically<br><br>For R_0=2.5: 80% of contacts traced and isolated in order to achieve control of 90% outbreaks.<br><br>For R_0=1.5 and no pre-symptomatic transmission, symptomatic isolation (no contact tracing) enough for epidemic control.<br><br>At 80% of contacts traced, the probability of achieving control fell from 89% to 31%, with a long delay from onset to isolation.<br><br>In scenarios in which only 10% of cases were asymptomatic, the probability that outbreaks were controlled by isolation and contact tracing for all values of contact tracing decreased: for 80% of contacts traced, only 37% of outbreaks were controlled, compared with 89% without subclinical infection.	7<br>No social mixing or multi-layer network
Huamani et al. [61]<br>Peru;<br>SBP	(1) Isolation of symptomatic individuals and contact tracing. Two scenarios are modelled: Pre-quarantine (before national lockdown) and Post-quarantine (after lift of lockdown). Comparator: no intervention	Results are presented graphically<br><br>Intervention (1) in pre-quarantine: with isolation of symptomatic from the first case/ fifth case/ tenth case, 100% success in contact tracing: probability of outbreak control = 80% / 40%/ 20%.<br><br>Intervention (1) in post-quarantine: early isolation of symptomatic: probability of outbreak control = 80%.<br><br>After lockdown, 100% of cases need to be traced for outbreak control.	7<br>No social mixing or multi-layer network
James et al. [62]<br>New Zealand;<br>SBP	(1) Isolation and contact tracing with different levels of coverage, varying levels of delay to contact tracing and varying efficacy of isolation and quarantine. Different levels of Intervention 1 are compared to each other and to no intervention	Results are presented graphically<br><br>Intervention (1) with fast contact tracing: R_{eff} is lower when the proportion of contacts traced is high rather than low.<br><br>Intervention (1) with slow contact tracing (i.e. mean tracing time > 5 days), R_{eff} similar to no contact tracing.<br><br>Mean tracing time > 6 days: faster tracing is more effective than increasing proportion of contacts traced.	7<br>No social mixing or multi-layer network
Kinoshita et al. [63]<br>Unclear;<br>SBP	(1) Contact tracing and isolation given different levels of effectiveness of contact tracing, and changes in relative infectiousness of asymptomatic individuals compared to symptomatic. Comparator: no contact tracing and isolation	Results are presented graphically<br><br>75% contact tracing and isolation with 40% asymptomatic and high transmission (R=2.5), low probability of epidemic extinction (28%-32%)<br><br>90% contact tracing and isolation with 40% asymptomatic and low transmission (R=1.5), probability of major epidemic is 80%	7<br>No social mixing or multi-layer network

Annex 5 (cont.). Expanded results: mathematical modelling studies.

Authors;<br>Setting;<br>Type of modelling approach	Intervention(s) and Comparator	Results as applicable to contact tracing	Quality score/ comments (studies with score <8)
Kretzschmar et al. 1 [64]<br>The Netherlands;<br>SBP	(1) Physical distancing; (2) testing and isolation of index cases; (3) Conventional contact tracing with different tracing delays; (4) App-based tracing with different levels of app coverage. Strategies (3) and (4) are compared to (1) and (2)	Results are presented graphically<br><br>R_{eff} < 1 with contact tracing is achieved only if testing delay ~ 2 days, or tracing delay is 1 day, or tracing coverage at least 80%.<br><br>R_{eff} < 1 cannot be achieved with perfect contact tracing (100% testing and tracing coverage) if testing delay is 3+ days.<br><br>If testing delay is 5+ days, app-based contact tracing is not more effective than conventional contact tracing.	7<br>No social mixing or multi-layer network
Kretzschmar et al. 2 [65]<br>The Netherlands;<br>SBP	((1) Multiple scenarios of contact tracing, social distancing and tracing coverage. Comparator: Baseline scenario = when a case is diagnosed, he/she is isolated, 100% traced, 0 days to find and isolate both household and non-household contacts.	Results are presented graphically<br><br>When testing delay is kept at 0 days, for a tracing coverage of 40% and higher, R_{eff} < 1,<br><br>If tracing coverage is 100%, testing delay can be at most 1 day to keep R_{eff} < 1.<br><br>For R_0=2.5 control is not possible with isolation and contact tracing, if less than 80% of all infected persons develop symptoms or are otherwise not detected by the healthcare system.<br><br>If less than 60% of infections are symptomatic and ascertained, the impact of contact tracing on the doubling time is small.	7<br>No social mixing or multi-layer network
Ng et al. [66]<br>Taiwan;<br>SBP	(1) Case-based intervention (case isolation, contact tracing and quarantine for 7/14 days regardless of symptoms), (2) population-based intervention (social distancing and mask use) and (3) Intervention (1) + Intervention (2). Comparator: no case isolation, contact tracing and quarantine	Results presented graphically<br><br>Contac tracing 90% and mask wearing: Achieves R<1 with 90% probability as long as social distancing reduces R to 1.2	7<br>No social mixing or multi-layer network
Endo et al. [67]<br>Unclear;<br>SBP	(1) Forward tracing only, (2) Forward + backward tracing. The interventions are compared to each other	Results presented graphically<br><br>Intervention (2): Effectiveness of contact tracing increased by a factor of 2-3.<br><br>With backward contact tracing, 2-3 times more generation 3 cases are avoided in a highly dispersed outbreak compared to a less-dispersed outbreak	6<br>No social mixing or multi-layer network, no distinction between levels of infectiousness
Huang et al. [68]<br>Singapore;<br>SBP	(1) Contact tracing, (2) self-awareness to exposure, and (3) Intervention (1) + Intervention (2) during three stages: Stage 1 was 23 Jan-16 March 2020, Stage 2 was 17 March-7 April 2020 (restrictions on non-essential traveling to countries affected by COVID-19 and 14-day quarantine for returning travelers introduced), Stage 3 was from 7 April (stringent social distancing measures introduced). Strategies are compared to each other and to no intervention	Results presented graphically<br><br>In order to stop the disease spreading in Stage 3, social distancing needs to be in place to reduce the reproduction number under 1.5 in order for contact tracing to be effective	6<br>No social mixing or multi-layer network, no distinction between levels of infectiousness

Annex 5 (cont.). Expanded results: mathematical modelling studies.

Authors; Setting; Type of modelling approach	Intervention(s) and Comparator	Results as applicable to contact tracing	Quality score/ comments (studies with score <8)
Peak et al. [69]<br>No specific country;<br>SBP	(1) Active monitoring of contacts and (2) Individual quarantine under two settings which vary in the probability of tracing an infected contact, contact tracing delay, reduction of infectiousness during quarantine for pre-symptomatic contacts, frequency of monitoring symptoms in pre-symptomatic contacts, reduction of infectiousness during isolation as follows: High feasibility setting (probability of tracing = 0.9, 0.5 days contact tracing delay, 75% reduction in infectiousness for contacts in quarantine, frequency of monitoring symptoms in pre-symptomatic contacts = 0.5 days, isolation reduces infectiousness by 90%) and Low feasibility setting (0.5, 2 days, 0.25, 2 days, 0.5). Comparator: no contact tracing	Results presented graphically<br><br>High feasibility setting short serial interval/ long serial interval: median R_{eff}=0.57/0.49 with quarantine and R_{eff}=1.55/0.54 with active monitoring of contacts.<br><br>Low feasibility setting, R_{eff}<1 was not achieved with either intervention.<br><br>With R_0 = 2.2, R_{eff} < 1 unless onset of infectiousness preceded symptoms by > 2 days.<br><br>High feasibility setting, no other NPIs: > 75% of contacts must be traced for R_{eff} < 1.<br><br>With short serial interval, if R_0 = 1.25 (via social distancing interventions), active monitoring of 50% of contacts may yield outbreak control.	6<br>No social mixing or multi-layer network, no distinction between levels of infectiousness
Cencetti et al. [70]<br>Unspecified limited geographical area;<br>Other	(1) Digital contact tracing with different levels of app adoption and quarantine of identified contacts under different levels of quarantine efficiency. Comparator: no digital contact tracing	Results presented graphically<br><br>If R_0=2, outbreak cannot be contained independently of level of app adoption<br><br>If R_0=1.5, 40% app adoption yields epidemic control if isolation is 80% efficient.<br><br>If R_0=1.2, 20% app adoption yields epidemic control if isolation is 50% efficient.<br><br>If R_0 > 1 with no digital contact tracing there will be an exponential outbreak.	9
Kucharski et al [71]<br>UK;<br>Other	Different combinations of self-isolation, contact tracing and physical distancing:<br>(1) Self-isolation within home; (2) Self-isolation outside home; (3) Self-isolation + household quarantine; (4) Intervention (3) + school or work contact tracing (100% traceable); (5) Intervention (3) + manual contact tracing of acquaintances (53% traceable); (6) Intervention . (3) + manual contact tracing of all contacts (100% traceable); (7) Intervention (3) + digital (app) contact tracing (53% traceable); (8) Intervention (3) + manual contact tracing of acquaintances + digital contact tracing (90% traceable at work, 79% traceable in school, 52% traceable other, 53% traceable with app); (9) Intervention (3) + manual contact tracing of acquaintances (90% traceable at work, 79% traceable in school, 52% traceable other) + limit of 4 daily contacts; (10) Intervention (3) + manual contact tracing of acquaintances (same as above) + app-based tracing (same as above) + limit of 4 daily contacts; and (11) Mass-testing of 5% of the population per week. Comparator: no intervention	Results are presented graphically<br><br>Compared to no intervention, the % reduction in R_{eff} achieved for each intervention is (1) 29%, (2) 35%, (3) 37%, (4) 53%, (5) 57%, (6) 64%, (7) 47%, (8) 61%, (9) 64%, (10) 66%, (11) 2%.	9

Annex 5 (cont.). Expanded results: mathematical modelling studies.

**Authors; Setting; Type of modelling approach**	**Intervention(s) and Comparator**	**Results as applicable to contact tracing**	**Quality score/ comments (studies with score <8)**
Ferretti et al. [72]<br>Not specified;<br>Other	(1) Isolation and manual contact tracing (3 days frm start of symptoms to isolation and contact quarantine), (2) isolation and manual contact tracing (2 days from start of symptoms to isolation and contact quarantine), isolation and manual contact tracing (1 day from start of symptoms to isolation and contact quarantine), isolation and digital contact tracing (instantaneous contact tracing). All interventions are modelled for a range of success rates in isolation and contact tracing. Comparator: no contact tracing	Results are presented graphically<br><br>If delay in case isolation and contact quarantine is 3+ days, there is no epidemic control independently of the level of contact tracing.<br><br>Contact tracing leads to R<1 if time to case isolation and contact quarantine is less than three days and there is high success in isolating cases and quarantining contacts.<br><br>With digital contact tracing, instant tracing and high success in isolating cases and quarantining contacts (=70%): R<1	7<br>No social mixing or multi-layer network
Grassly et al. [73]<br>UK;<br>Other	(1) Self-isolation of symptomatic individuals; (2) weekly PCR testing of health care workers and high-risk groups with different assumptions on test sensitivity; (3) testing symptomatic individuals with varying testing rates, tracing their contacts with a varying coverage + quarantining contacts of individuals who test positive. Delays in testing and tracing are also explored. Comparator: Intervention (1)	Results are presented graphically<br><br>For 80% coverage in testing and tracing and 24 hours from sample collection to quarantine of contacts, drop in R_{eff} = 26%, compared to Intervention (1).<br><br>For 50% coverage in testing and tracing and 48 hours delay in quarantine of contacts, drop in R_{eff} = 8%, compared to no intervention.	7<br>No social mixing or multi-layer network
Sanche et al. [74]<br>China;<br>Other	(1) Contact tracing, (2) quarantine and (3) social distancing. Comparator: no intervention	Results presented graphically<br><br>Contact tracing of symptomatic persons is effective if the fraction of unidentified persons is low. When 20% of symptomatic persons are undetected, high levels of social distancing will lead to epidemic control.	7<br>No social mixing or multi-layer network
Kim et al. [78]<br>Unclear;<br>Other	(1) Automated contact tracing with a variable fraction of the infected population which is confirmed positive by testing, a variable fraction of app users who confirmed they are diagnosed positive, and variable probabilities of disease transmission. Comparator: no intervention	Results presented graphically. /<br>With a modest probability of transmission (e.g. about 30%) quite a large fraction of the population (about 40–60%) needs to be enrolled in automated contact tracing to achieve R<1	6<br>No social mixing or multi-layer network, no distinction between levels of infectiousness
Kuzdeuov et al. [75]<br>Lecco (Lombardy);<br>Other	(1) Random testing and isolation with different levels, (2) digital contact tracing with different levels of coverage, (3) Intervention (1)+(2). Comparator: no intervention	Results presented graphically.<br><br>Number of daily tests per thousand people = 5/ 20/ 30/ 40) with 0% contact tracing app adoption. The number of infected at the peak of the epidemic is reduced by 26%/ 41%/ 51%/ 63%<br><br>Contact tracing app adoption = (0.25, 0.5, 0.75, 1) with 0% testing: The number of infected at the peak of the epidemic is reduced by 9%/ 18%/ 38%/ 54%<br><br>Random testing (10 tests per thousand per day and digital contact tracing with 50% coverage: 20% reduction in infections	6<br>No social mixing or multi-layer network, parameters not fully described

Annex 5 (cont.). Expanded results: mathematical modelling studies.

Authors; Setting; Type of modelling approach	Intervention(s) and Comparator	Results as applicable to contact tracing	Quality score/ comments (studies with score <8)
Moran et al [76] England; Other	(1) Find, track, trace and isolate (FTTI) with 5% policy efficacy; (2) FTTI with 20% policy efficacy; FTTI with 40% policy efficacy; (4) FTTI policy with 60% policy efficacy. All interventions modelled with some social distancing Comparator: FTTI with 0% policy efficacy.	Results are presented graphically<br><br>With medium-term immunity, FTTI with 40% efficacy or higher leads to a reduction in deaths of 99%. Even small-scale contact tracing strategies are effective.	6<br>No social mixing or multi-layer network, limited representation of the model
Worden et al [77] San Francisco (USA); Other	(1) Contact tracing; (2) Mask wearing; (3) interventions (1) + (2). Comparator: Shelter in place order	Results are presented graphically<br><br>Shelter in place order without contact tracing: 11% increase in cases per day; Shelter in place order without contact tracing or mask wearing: 30% increase in cases per day	6<br>No social mixing or multi-layer network, no distinction between levels of infectiousness

REFERENCES:

1. Wymant C, Ferretti L, Tsallis D, Charalambides M, Abeler-Dörner L, Bonsall D, et al. The epidemiological impact of the NHS COVID-19 App. Nature. 2021.DOI: 10.1038/s41586-021-03606-z

2. Kendall M, Milsom L, Abeler-Dörner L, Wymant C, Ferretti L, Briers M, et al. Epidemiological changes on the Isle of Wight after the launch of the NHS Test and Trace programme: a preliminary analysis. The Lancet Digital Health. 2020;2(12):e658-e66.DOI: 10.1016/S2589-7500(20)30241-7

3. Pozo-Martin F, Weishaar H, Cristea F, Hanefeld J, Bahr T, Schaade L, et al. The impact of non-pharmaceutical interventions on COVID-19 epidemic growth in the 37 OECD member states. European Journal of Epidemiology. 2021.DOI: 10.1007/s10654-021-00766-0

4. Haug N, Geyrhofer L, Londei A, Dervic E, Desvars-Larrive A, Loreto V, et al. Ranking the effectiveness of worldwide COVID-19 government interventions. Nature Human Behaviour. 2020;4(12):1303-12.DOI: 10.1038/s41562-020-01009-0

5. Liu Y, Morgenstern C, Kelly J, Lowe R, Munday J, Villabona-Arenas CJ, et al. The impact of non-pharmaceutical interventions on SARS-CoV-2 transmission across 130 countries and territories. BMC Medicine. 2021;19(1).DOI: 10.1186/s12916-020-01872-8

6. Vecino-Ortiz AI, Congote JV, Bedoya SZ, Cucunuba ZM. Impact of contact tracing on COVID-19 mortality: An impact evaluation using surveillance data from Colombia. PLoS ONE. 2021;16(3 March).DOI: 10.1371/journal.pone.0246987

7. Leffler CT, Ing E, Lykins JD, Hogan MC, McKeown CA, Grzybowski A. Association of country-wide coronavirus mortality with demographics, testing, lockdowns, and public wearing of masks. American Journal of Tropical Medicine and Hygiene. 2020;103(6):2400-11.DOI: 10.4269/ajtmh.20-1015

8. Wibbens PD, Koo WW-Y, McGahan AM. Which COVID policies are most effective? A Bayesian analysis of COVID-19 by jurisdiction. PLOS ONE. 2021;15(12):e0244177.DOI: 10.1371/journal.pone.0244177

9. Hong SH, Hwang H, Park MH. Effect of COVID-19 non-pharmaceutical interventions and the implications for human rights. International Journal of Environmental Research and Public Health. 2021;18(1):1-12.DOI: 10.3390/ijerph18010217

10. Papadopoulos DI, Donkov I, Charitopoulos K, Bishara S. The impact of lockdown measures on COVID-19: a worldwide comparison. medRxiv. 2020:2020.05.22.20106476.DOI: 10.1101/2020.05.22.20106476

11. Malheiro R, Figueiredo AL, Magalhães JP, Teixeira P, Moita I, Moutinho MC, et al. Effectiveness of contact tracing and quarantine on reducing COVID-19 transmission: a retrospective cohort study. Public Health. 2020;189:54-9.DOI: 10.1016/j.puhe.2020.09.012

12. Park Y, Huh IS, Lee J, Kang CR, Cho S-i, Ham HJ, et al. Application of Testing-Tracing-Treatment Strategy in Response to the COVID-19 Outbreak in Seoul, Korea. J Korean Med Sci. 2020;35(45)

13. Abueg M, Hinch R, Wu N, Liu L, Probert W, Wu A, et al. Modeling the effect of exposure notification and non-pharmaceutical interventions on COVID-19 transmission in Washington state. npj Digital Medicine. 2021;4(1).DOI: 10.1038/s41746-021-00422-7

14. Aleta A, Martín-Corral D, Pastore y Piontti A, Ajelli M, Litvinova M, Chinazzi M, et al. Modelling the impact of testing, contact tracing and household quarantine on second waves of COVID-19. Nature Human Behaviour. 2020;4(9):964-71.DOI: 10.1038/s41562-020-0931-9

15. Bicher M, Rippinger C, Urach C, Brunmeir D, Siebert U, Popper N. Evaluation of Contact-Tracing Policies against the Spread of SARS-CoV-2 in Austria: An Agent-Based Simulation. Medical decision making : an international journal of the Society for Medical Decision Making. 2021;41(8):1017-32.DOI: 10.1177/0272989x211013306

16. Li J, Giabbanelli PJ, editors. Identifying Synergistic Interventions to Address COVID-19 Using a Large Scale Agent-Based Model. Computational Science – ICCS 2021; 2021 2021//; Cham: Springer International Publishing.

17. Gressman PT, Peck JR. Simulating COVID-19 in a university environment. Mathematical Biosciences. 2020;328.DOI: 10.1016/j.mbs.2020.108436

18. Hill EM, Atkins BD, Keeling MJ, Tildesley MJ, Dyson L. Modelling SARS-CoV-2 transmission in a UK university setting. Epidemics. 2021;36:100476.DOI: https://doi.org/10.1016/j.epidem.2021.100476

19. Hill EM, Atkins BD, Keeling MJ, Dyson L, Tildesley MJ. A network modelling approach to assess non-pharmaceutical disease controls in a worker population: An application to SARS-CoV-2. PLOS Computational Biology. 2021;17(6):e1009058.DOI: 10.1371/journal.pcbi.1009058

20. Kerr CC, Mistry D, Stuart RM, Rosenfeld K, Hart GR, Núñez RC, et al. Controlling COVID-19 via test-trace-quarantine. Nature Communications. 2021;12(1).DOI: 10.1038/s41467-021-23276-9

21. Luo T, Cao C, Wang Y, Zeng D, Zhang Q. Role of Asymptomatic COVID-19 Cases in Viral Transmission: Findings From a Hierarchical Community Contact Network Model. IEEE Transactions on Automation Science and Engineering. 2022;19(2):576-85.DOI: 10.1109/TASE.2021.3106782

22. Moreno López Jesús A, Arregui García B, Bentkowski P, Bioglio L, Pinotti F, Boëlle P-Y, et al. Anatomy of digital contact tracing: Role of age, transmission setting, adoption, and case detection. Science Advances.7(15):eabd8750.DOI: 10.1126/sciadv.abd8750

23. Ng V, Fazil A, Waddell LA, Bancej C, Turgeon P, Otten A, et al. Projected effects of nonpharmaceutical public health interventions to prevent resurgence of SARS-CoV-2 transmission in Canada. CMAJ. 2020;192(37):E1053-E64.DOI: 10.1503/cmaj.200990

24. Pham TM, Tahir H, van de Wijgert JHHM, Van der Roest BR, Ellerbroek P, Bonten MJM, et al. Interventions to control nosocomial transmission of SARS-CoV-2: a modelling study. BMC Medicine. 2021;19(1):211.DOI: 10.1186/s12916-021-02060-y

25. Scott N, Palmer A, Delport D, Abeysuriya R, Stuart RM, Kerr CC, et al. Modelling the impact of relaxing COVID-19 control measures during a period of low viral transmission. Medical Journal of Australia. 2021;214(2):79-83.DOI: 10.5694/mja2.50845

26. Shamil MS, Farheen F, Ibtehaz N, Khan IM, Rahman MS. An Agent-Based Modeling of COVID-19: Validation, Analysis, and Recommendations. Cognitive Computation. 2021.DOI: 10.1007/s12559-020-09801-w

27. Stuart RM, Abeysuriya RG, Kerr CC, Mistry D, Klein DJ, Gray RT, et al. Role of masks, testing and contact tracing in preventing COVID-19 resurgences: A case study from New South Wales, Australia. BMJ Open. 2021;11(4).DOI: 10.1136/bmjopen-2020-045941

28. Tatapudi H, Das R, Das TK. Impact assessment of full and partial stay-at-home orders, face mask usage, and contact tracing: An agent-based simulation study of COVID-19 for an urban region. Glob Epidemiol. 2020;2:100036-.DOI: 10.1016/j.gloepi.2020.100036

29. Thompson J, Wattam S. Estimating the impact of interventions against COVID-19: From lockdown to vaccination. PLOS ONE. 2021;16(12):e0261330.DOI: 10.1371/journal.pone.0261330

30. Wells K, Lurgi M, Collins B, Lucini B, Kao RR, Lloyd AL, et al. Disease control across urban-rural gradients. Journal of the Royal Society Interface. 2020;17(173).DOI: 10.1098/rsif.2020.0775rsif20200775

31. Willem L, Abrams S, Libin PJK, Coletti P, Kuylen E, Petrof O, et al. The impact of contact tracing and household bubbles on deconfinement strategies for COVID-19. Nature Communications. 2021;12(1).DOI: 10.1038/s41467-021-21747-7

32. Barthe G, Viti RD, Druschel P, Garg D, Gomez-Rodriguez M, Ingo P, et al. Listening to bluetooth beacons for epidemic risk mitigation. Scientific Reports. 2022;12(1):5558.DOI: 10.1038/s41598-022-09440-1

33. Bhattacharyya C, Vinay V. Suppress, and Not Just Flatten: Strategies for Rapid Suppression of COVID19 Transmission in Small World Communities. J Indian Inst Sci. 2020:1-14.DOI: 10.1007/s41745-020-00209-x

34. Colomer MÀ, Margalida A, Alòs F, Oliva-Vidal P, Vilella A, Fraile L. Modeling of vaccination and contact tracing as tools to control the covid-19 outbreak in Spain. Vaccines. 2021;9(4).DOI: 10.3390/vaccines9040386

35. Eilersen A, Sneppen K. Cost–benefit of limited isolation and testing in COVID-19 mitigation. Scientific Reports. 2020;10(1):18543.DOI: 10.1038/s41598-020-75640-2

36. Goldberg LA, Jorritsma J, Komjathy J, Lapinskas J. Increasing efficacy of contact-tracing applications by user referrals and stricter quarantining. PLoS ONE. 2021;16(5 May).DOI: 10.1371/journal.pone.0250435

37. Goldenbogen B, Adler SO, Bodeit O, Wodke JAH, Korman A, Bonn L, et al. Geospatial precision simulations of community confined human interactions during SARS-CoV-2 transmission reveals bimodal intervention outcomes. medRxiv. 2020:2020.05.03.20089235.DOI: 10.1101/2020.05.03.20089235

38. Low M, Geffen N. Contact tracing and isolation reduces covid-19 incidence in a structured agent-based model. medRxiv. 2020:2020.10.06.20207761.DOI: 10.1101/2020.10.06.20207761

39. Moon SA, Scoglio CM. Contact tracing evaluation for COVID-19 transmission in the different movement levels of a rural college town in the USA. Scientific Reports. 2021;11(1):4891.DOI: 10.1038/s41598-021-83722-y

40. Mukherjee UK, Bose S, Ivanov A, Souyris S, Seshadri S, Sridhar P, et al. Evaluation of reopening strategies for educational institutions during COVID-19 through agent based simulation. Scientific Reports. 2021;11(1):6264.DOI: 10.1038/s41598-021-84192-y

41. Panovska-Griffiths J, Kerr CC, Stuart RM, Mistry D, Klein DJ, Viner RM, et al. Determining the optimal strategy for reopening schools, the impact of test and trace interventions, and the risk of occurrence of a second COVID-19 epidemic wave in the UK: a modelling study. The Lancet Child and Adolescent Health. 2020;4(11):817-27.DOI: 10.1016/S2352-4642(20)30250-9

42. Pollmann TR, Schönert S, Müller J, Pollmann J, Resconi E, Wiesinger C, et al. The impact of digital contact tracing on the SARS-CoV-2 pandemic—a comprehensive modelling study. EPJ Data Science. 2021;10(1):37.DOI: 10.1140/epjds/s13688-021-00290-x

43. Fiore VG, DeFelice N, Glicksberg BS, Perl O, Shuster A, Kulkarni K, et al. Containment of COVID-19: Simulating the impact of different policies and testing capacities for contact tracing, testing, and isolation. PLoS ONE. 2021;16(3 March 2021).DOI: 10.1371/journal.pone.0247614

44. Geffen N, Low M. Isolation of infected people and their contacts is likely to be effective against many short-term epidemics. medRxiv. 2020:2020.10.07.20207845.DOI: 10.1101/2020.10.07.20207845

45. Quilty BJ, Clifford S, Hellewell J, Russell TW, Kucharski AJ, Flasche S, et al. Quarantine and testing strategies in contact tracing for SARS-CoV-2: a modelling study. The Lancet Public Health. 2021;6(3):e175-e83.DOI: 10.1016/S2468-2667(20)30308-X

46. Tuomisto JT, Yrjölä J, Kolehmainen M, Bonsdorff J, Pekkanen J, Tikkanen T. An agent-based epidemic model REINA for COVID-19 to identify destructive policies. medRxiv. 2020:2020.04.09.20047498.DOI: 10.1101/2020.04.09.20047498

47. Wallentin G, Kaziyeva D, Reibersdorfer-Adelsberger E. COVID-19 Intervention Scenarios for a Long-term Disease Management. International Journal of Health Policy and Management. 2020;9(12):508-16.DOI: 10.34172/ijhpm.2020.130

48. Zafarnejad R, Griffin PM. Assessing school-based policy actions for COVID-19: An agent-based analysis of incremental infection risk. Computers in Biology and Medicine. 2021;134.DOI: 10.1016/j.compbiomed.2021.104518

49. Reich O, Shalev G, Kalvari T. Modeling COVID-19 on a network: super-spreaders, testing and containment. medRxiv. 2020:2020.04.30.20081828.DOI: 10.1101/2020.04.30.20081828

50. Reich O. COVID-19 Test & Trace Success Determinants: Modeling On A Network. medRxiv. 2020:2020.08.05.20168799.DOI: 10.1101/2020.08.05.20168799

51. Plank MJ, James A, Lustig A, Steyn N, Binny RN, Hendy SC. Potential reduction in transmission of COVID-19 by digital contact tracing systems: a modelling study. Mathematical Medicine and Biology: A Journal of the IMA. 2022;39(2):156-68.DOI: 10.1093/imammb/dqac002

52. Allali M, Portecop P, Carlès M, Gibert D. Model of a Testing-and-Quarantine Strategy to Slow-Down the COVID-19 Outbreak in Guadeloupe. medRxiv. 2020:2020.05.01.20088138.DOI: 10.1101/2020.05.01.20088138

53. Bradshaw WJ, Alley EC, Huggins JH, Lloyd AL, Esvelt KM. Bidirectional contact tracing could dramatically improve COVID-19 control. Nature Communications. 2021;12(1).DOI: 10.1038/s41467-020-20325-7

54. Bradshaw W, Huggins J, Lloyd A, Esvelt K. The feasibility of targeted test-trace-isolate for the control of SARS-CoV-2 variants. F1000Research. 2021;10(291).DOI: 10.12688/f1000research.51164.1

55. Brook CE, Northrup GR, Ehrenberg AJ, Doudna JA, Boots M. Optimizing COVID-19 control with asymptomatic surveillance testing in a university environment. Epidemics. 2021;37:100527.DOI: https://doi.org/10.1016/j.epidem.2021.100527

56. Davis EL, Lucas TCD, Borlase A, Pollington TM, Abbott S, Ayabina D, et al. Contact tracing is an imperfect tool for controlling COVID-19 transmission and relies on population adherence. Nature Communications. 2021;12(1):5412.DOI: 10.1038/s41467-021-25531-5

57. Filonets T, Solovchuk M, Gao W, Sheu TWH. Investigation of the efficiency of mask wearing, contact tracing, and case isolation during the covid-19 outbreak. Journal of Clinical Medicine. 2021;10(13).DOI: 10.3390/jcm10132761

58. Firth JA, Hellewell J, Klepac P, Kissler S, Jit M, Atkins KE, et al. Using a real-world network to model localized COVID-19 control strategies. Nature Medicine. 2020;26(10):1616-22.DOI: 10.1038/s41591-020-1036-8

59. Fyles M, Fearon E, Overton C, null n, Wingfield T, Medley GF, et al. Using a household-structured branching process to analyse contact tracing in the SARS-CoV-2 pandemic. Philosophical Transactions of the Royal Society B: Biological Sciences. 2021;376(1829):20200267.DOI: 10.1098/rstb.2020.0267

60. Hellewell J, Abbott S, Gimma A, Bosse NI, Jarvis CI, Russell TW, et al. Feasibility of controlling COVID-19 outbreaks by isolation of cases and contacts. The Lancet Global Health. 2020;8(4):e488-e96.DOI: 10.1016/S2214-109X(20)30074-7

61. Huamaní C, Timaná-Ruiz R, Pinedo J, Pérez J, Vásquez L. Estimated conditions to control the covid-19 pandemic in peruvian pre- and post-quarantine scenarios. Rev Peru Med Exp Salud Publica. 2020;37(2):195-202.DOI: 10.17843/rpmesp.2020.372.5405

62. James A, Plank MJ, Hendy S, Binny R, Lustig A, Steyn N, et al. Successful contact tracing systems for COVID-19 rely on effective quarantine and isolation. PLoS ONE. 2021;16(6 June).DOI: 10.1371/journal.pone.0252499

63. Kinoshita R, Anzai A, Jung SM, Linton NM, Miyama T, Kobayashi T, et al. Containment, contact tracing and asymptomatic transmission of novel coronavirus disease (Covid-19): A modelling study. Journal of Clinical Medicine. 2020;9(10):1-9.DOI: 10.3390/jcm9103125

64. Kretzschmar ME, Rozhnova G, Bootsma MCJ, van Boven M, van de Wijgert JHHM, Bonten MJM. Impact of delays on effectiveness of contact tracing strategies for COVID-19: a modelling study. The Lancet Public Health. 2020;5(8):e452-e9.DOI: 10.1016/S2468-2667(20)30157-2

65. Kretzschmar ME, Rozhnova G, van Boven M. Isolation and Contact Tracing Can Tip the Scale to Containment of COVID-19 in Populations With Social Distancing. Frontiers in Physics. 2021;8(677).DOI: 10.3389/fphy.2020.622485

66. Ng T-C, Cheng H-Y, Chang H-H, Liu C-C, Yang C-C, Jian S-W, et al. Comparison of Estimated Effectiveness of Case-Based and Population-Based Interventions on COVID-19 Containment in Taiwan. JAMA Internal Medicine. 2021;181(7):913-21.DOI: 10.1001/jamainternmed.2021.1644

67. Endo A, Centre for the Mathematical Modelling of Infectious Diseases C-WG, Leclerc QJ, Knight GM, Medley GF, Atkins KE, et al. Implication of backward contact tracing in the presence of overdispersed transmission in COVID-19 outbreaks. Wellcome Open Res. 2021;5:239-.DOI: 10.12688/wellcomeopenres.16344.3

68. Huang Q, Wang L, Yang Y, Huang L, Du Z, Xiao G. CoVID-19 in Singapore: Impact of Contact Tracing and Self-awareness on Healthcare Demand. medRxiv. 2020:2020.06.04.20122879.DOI: 10.1101/2020.06.04.20122879

69. Peak CM, Kahn R, Grad YH, Childs LM, Li R, Lipsitch M, et al. Individual quarantine versus active monitoring of contacts for the mitigation of COVID-19: a modelling study. The Lancet Infectious Diseases. 2020;20(9):1025-33.DOI: 10.1016/S1473-3099(20)30361-3

70. Cencetti G, Santin G, Longa A, Pigani E, Barrat A, Cattuto C, et al. Digital proximity tracing on empirical contact networks for pandemic control. Nature Communications. 2021;12(1).DOI: 10.1038/s41467-021-21809-w

71. Kucharski AJ, Klepac P, Conlan AJK, Kissler SM, Tang ML, Fry H, et al. Effectiveness of isolation, testing, contact tracing, and physical distancing on reducing transmission of SARS-CoV-2 in different settings: a mathematical modelling study. The Lancet Infectious Diseases. 2020;20(10):1151-60.DOI: 10.1016/S1473-3099(20)30457-6

72. Ferretti L, Wymant C, Kendall M, Zhao L, Nurtay A, Abeler-Dörner L, et al. Quantifying SARS-CoV-2 transmission suggests epidemic control with digital contact tracing. Science. 2020;368(6491).DOI: 10.1126/science.abb6936

73. Grassly NC, Pons-Salort M, Parker EPK, White PJ, Ferguson NM, Ainslie K, et al. Comparison of molecular testing strategies for COVID-19 control: a mathematical modelling study. The Lancet Infectious Diseases. 2020;20(12):1381-9.DOI: 10.1016/S1473-3099(20)30630-7

74. Sanche S, Lin YT, Xu C, Romero-Severson E, Hengartner N, Ke R. RESEARCH High Contagiousness and Rapid Spread of Severe Acute Respiratory Syndrome Coronavirus 2. Emerging Infectious Diseases. 2020;26(7):1470-7.DOI: 10.3201/eid2607.200282

75. Kuzdeuov A, Karabay A, Baimukashev D, Ibragimov B, Varol HA. A Particle-Based COVID-19 Simulator With Contact Tracing and Testing. IEEE Open Journal of Engineering in Medicine and Biology. 2021;2:111-7.DOI: 10.1109/OJEMB.2021.3064506

76. Moran RJ, Billig AJ, Cullen M, Razi A, Daunizeau J, Leech R, et al. Using the LIST model to Estimate the Effects of Contact Tracing on COVID-19 Endemic Equilibria in England and its Regions. medRxiv. 2020:2020.06.11.20128611.DOI: 10.1101/2020.06.11.20128611

77. Worden L, Wannier R, Blumberg S, Ge AY, Rutherford GW, Porco TC. Estimation of effects of contact tracing and mask adoption on COVID-19 transmission in San Francisco: a modeling study. medRxiv. 2020:2020.06.09.20125831.DOI: 10.1101/2020.06.09.20125831

78. Kim H, Paul A. Automated contact tracing: A game of big numbers in the time of COVID-19. Journal of the Royal Society Interface. 2021;18(175).DOI: 10.1098/rsif.2020.0954

79. Rajabi A, Mantzaris AV, Mutlu EC, Garibay OO. Investigating Dynamics of COVID-19 Spread and Containment with Agent-Based Modeling. Applied Sciences. 2021;11(12):5367

80. Sarma U, Ghosh B. Country-specific optimization strategy for testing through contact tracing can help maintain a low reproduction number ($$R_{0}$$) during unlock. Scientific Reports. 2022;12(1):212.DOI: 10.1038/s41598-021-03846-z

81. Pung R, Clapham HE, Lee VJ, Kucharski AJ, group obotCC-w. Relative role of border restrictions, case finding and contact tracing in controlling SARS-CoV-2 in the presence of undetected transmission. medRxiv. 2021:2021.05.05.21256675.DOI: 10.1101/2021.05.05.21256675

82. Scarabel F, Pellis L, Ogden NH, Wu J. A renewal equation model to assess roles and limitations of contact tracing for disease outbreak control. Royal Society Open Science. 2021;8(4):202091.DOI: doi:10.1098/rsos.202091

83. Serafino M, Monteiro HS, Luo S, Reis SDS, Igual C, Lima Neto AS, et al. Digital contact tracing and network theory to stop the spread of COVID-19 using big-data on human mobility geolocalization. PLOS Computational Biology. 2022;18(4):e1009865.DOI: 10.1371/journal.pcbi.1009865

84. Ashcroft P, Lehtinen S, Angst DC, Low N, Bonhoeffer S. Quantifying the impact of quarantine duration on covid-19 transmission. eLife. 2021;10:1-33.DOI: 10.7554/eLife.63704

85. Bilinski A, Mostashari F, Salomon JA. Modeling Contact Tracing Strategies for COVID-19 in the Context of Relaxed Physical Distancing Measures. JAMA Network Open. 2020;3(8):e2019217-e.DOI: 10.1001/jamanetworkopen.2020.19217

86. Killeen GF, Kiware SS. Why lockdown? Why national unity? Why global solidarity? Simplified arithmetic tools for decision-makers, health professionals, journalists and the general public to explore containment options for the 2019 novel coronavirus. Infectious Disease Modelling. 2020;5:442-58.DOI: 10.1016/j.idm.2020.06.006

87. Ponte C, Carmona HA, Oliveira EA, Caminha C, Lima AS, Andrade JS, et al. Tracing contacts to evaluate the transmission of COVID-19 from highly exposed individuals in public transportation. Scientific Reports. 2021;11(1):24443.DOI: 10.1038/s41598-021-03998-y

88. Segal C, Zhang Z, Karras BT, Revere D, Zane G, Baseman JG. Early Epidemiological Evidence of Public Health Value of WA Notify, a Smartphone-based Exposure Notification Tool: Modeling COVID-19 Cases Averted in Washington State. medRxiv. 2021:2021.06.04.21257951.DOI: 10.1101/2021.06.04.21257951

89. Braun P, Haffner S, Woodcock BG. COVID-19 pandemic predictions using the modified Bateman SIZ model and observational data for Heidelberg, Germany: Effect of vaccination with a SARS-CoV-2 vaccine, coronavirus testing and application of the Corona-Warn-App. Int J Clin Pharmacol Ther. 2020;58(8):417-25.DOI: 10.5414/cp203846

90. Jeon S, Rainisch G, Lash RR, Moonan PK, Oeltmann JE, Greening BJ, et al. Estimates of Cases and Hospitalizations Averted by COVID-19 Case Investigation and Contact Tracing in 14 Health Jurisdictions in the United States. Journal of Public Health Management and Practice. 2022;28(1):16-24.DOI: 10.1097/phh.0000000000001420

91. Lambert A. A mathematical assessment of the efficiency of quarantining and contact tracing in curbing the COVID-19 epidemic. Math Model Nat Phenom. 2021;16:53

92. Malmberg H, Britton T. Inflow restrictions can prevent epidemics when contact tracing efforts are effective but have limited capacity: Inflow restrictions can prevent epidemics when contact tracing efforts are effective but have limited capacity. Journal of the Royal Society Interface. 2020;17(170).DOI: 10.1098/rsif.2020.0351rsif20200351

93. Afzal I, Abdul Raheem R, Rafeeq N, Moosa S. Contact Tracing for Containment of Novel Coronavirus Disease (COVID-19) in the Early Phase of the Epidemic in the Maldives. Asia Pacific Journal of Public Health. 2021;33(1):131-3.DOI: 10.1177/1010539520956447

94. Bracis C, Burns E, Moore M, Swan D, Reeves DB, Schiffer JT, et al. Widespread testing, case isolation and contact tracing may allow safe school reopening with continued moderate physical distancing: A modeling analysis of King County, WA data. Infectious Disease Modelling. 2021;6:24-35.DOI: 10.1016/j.idm.2020.11.003

95. Di Domenico L, Pullano G, Sabbatini CE, Boëlle P-Y, Colizza V. Impact of lockdown on COVID-19 epidemic in Île-de-France and possible exit strategies. BMC Medicine. 2020;18(1):240.DOI: 10.1186/s12916-020-01698-4

96. Fair JM, LeClaire RJ, Dauelsberg LR, Ewers M, Pasqualini D, Cleland T, et al. Systems dynamics and the uncertainties of diagnostics, testing and contact tracing for COVID-19. Methods. 2021;195:77-91.DOI: https://doi.org/10.1016/j.ymeth.2021.03.008

97. Ferrari A, Santus E, Cirillo D, Ponce-de-Leon M, Marino N, Ferretti MT, et al. Simulating SARS-CoV-2 epidemics by region-specific variables and modeling contact tracing app containment. npj Digital Medicine. 2021;4(1).DOI: 10.1038/s41746-020-00374-4

98. Forgoston E, Thorne MAS. Strategies for Controlling the Spread of COVID-19. medRxiv. 2020:2020.06.24.20139014.DOI: 10.1101/2020.06.24.20139014

99. Goscé L, Phillips PA, Spinola P, Gupta DRK, Abubakar PI. Modelling SARS-COV2 Spread in London: Approaches to Lift the Lockdown. Journal of Infection. 2020;81(2):260-5.DOI: https://doi.org/10.1016/j.jinf.2020.05.037

100. Grimm V, Mengel F, Schmidt M. Extensions of the SEIR model for the analysis of tailored social distancing and tracing approaches to cope with COVID-19. Scientific Reports. 2021;11(1):4214.DOI: 10.1038/s41598-021-83540-2

101. Lunz D, Batt G, Ruess J. To quarantine, or not to quarantine: A theoretical framework for disease control via contact tracing. Epidemics. 2021;34.DOI: 10.1016/j.epidem.2020.100428

102. Min K-D, Kang H, Lee J-Y, Jeon S, Cho S-i. Estimating the Effectiveness of Non-Pharmaceutical Interventions on COVID-19 Control in Korea. J Korean Med Sci. 2020;35(35)

103. Struben J. The coronavirus disease (COVID-19) pandemic: simulation-based assessment of outbreak responses and postpeak strategies. System Dynamics Review. 2020;36(3):247-93.DOI: https://doi.org/10.1002/sdr.1660

104. Teimouri A. An SEIR Model with Contact Tracing and Age-Structured Social Mixing for COVID-19 outbreak. medRxiv. 2020:2020.07.05.20146647.DOI: 10.1101/2020.07.05.20146647

105. Zhong L. A dynamic pandemic model evaluating reopening strategies amid COVID-19. PLoS ONE. 2021;16(3 March).DOI: 10.1371/journal.pone.0248302

106. Amaku M, Covas DT, Bezerra Coutinho FA, Azevedo Neto RS, Struchiner C, Wilder-Smith A, et al. Modelling the test, trace and quarantine strategy to control the COVID-19 epidemic in the state of São Paulo, Brazil. Infectious Disease Modelling. 2021;6:46-55.DOI: 10.1016/j.idm.2020.11.004

107. Amaku M, Covas DT, Coutinho FAB, Azevedo RS, Massad E. Modelling the impact of contact tracing of symptomatic individuals on the COVID-19 epidemic. Clinics. 2021;76:e2639.DOI: https://doi.org/10.6061/clinics/2021/e2639

108. Ansah JP, Matchar DB, Wei SLS, Low JG, Pourghaderi AR, Siddiqui FJ, et al. The effectiveness of public health interventions against COVID-19: Lessons from the Singapore experience. PLoS ONE. 2021;16(3 March).DOI: 10.1371/journal.pone.0248742

109. Barrat A, Cattuto C, Kivelä M, Lehmann S, Saramäki J. Effect of manual and digital contact tracing on COVID-19 outbreaks: A study on empirical contact data. Journal of the Royal Society Interface. 2021;18(178).DOI: 10.1098/rsif.2020.1000

110. Burdinski A, Brockmann D, Maier BF. Digital contact tracing contributes little to COVID-19 outbreak containment. medRxiv. 2021:2021.06.21.21259258.DOI: 10.1101/2021.06.21.21259258

111. Currie D, Peng C, Lyle D, Jameson B, Frommer M. Stemming the flow: how much can the Australian smartphone app help to control COVID-19? Public Health Research & Practice.

112. Drake JM, Dahlin K, Rohani P, Handel A. Five approaches to the suppression of SARS-CoV-2 without intensive social distancing. Proceedings of the Royal Society B: Biological Sciences. 2021;288(1949):20203074.DOI: doi:10.1098/rspb.2020.3074

113. Giordano G, Blanchini F, Bruno R, Colaneri P, Di Filippo A, Di Matteo A, et al. Modelling the COVID-19 epidemic and implementation of population-wide interventions in Italy. Nature Medicine. 2020;26(6):855-60.DOI: 10.1038/s41591-020-0883-7

114. Grantz KH, Lee EC, D'Agostino McGowan L, Lee KH, Metcalf JCE, Gurley ES, et al. Maximizing and evaluating the impact of test-trace-isolate programs: A modeling study. PLoS Medicine. 2021;18(4).DOI: 10.1371/journal.pmed.1003585

115. Humphrey L, Thommes EW, Fields R, Coudeville L, Hakim N, Chit A, et al. Testing, Tracing and Social Distancing: Assessing Options for the Control of Covid-19. medRxiv. 2020:2020.04.23.20077503.DOI: 10.1101/2020.04.23.20077503

116. Johnson KE, Stoddard M, Nolan RP, White DE, Hochberg N, Chakravarty A. This time is different: model-based evaluation of the implications of SARS-CoV-2 infection kinetics for disease control. medRxiv. 2020:2020.08.19.20177550.DOI: 10.1101/2020.08.19.20177550

117. Keeling MJ, Hollingsworth TD, Read JM. Efficacy of contact tracing for the containment of the 2019 novel coronavirus (COVID-19). Journal of Epidemiology and Community Health. 2020;74(10):861-6.DOI: 10.1136/jech-2020-214051

118. Müller SA, Balmer M, Charlton W, Ewert R, Neumann A, Rakow C, et al. Predicting the effects of COVID-19 related interventions in urban settings by combining activity-based modelling, agent-based simulation, and mobile phone data. PLOS ONE. 2021;16(10):e0259037.DOI: 10.1371/journal.pone.0259037

119. Nakamoto I, Wang S, Guo Y, Zhuang W. A QR Code–Based Contact Tracing Framework for Sustainable Containment of COVID-19: Evaluation of an Approach to Assist the Return to Normal Activity. JMIR Mhealth Uhealth. 2020;8(9):e22321.DOI: 10.2196/22321

120. Ngonghala CN, Iboi E, Eikenberry S, Scotch M, MacIntyre CR, Bonds MH, et al. Mathematical assessment of the impact of non-pharmaceutical interventions on curtailing the 2019 novel Coronavirus. Mathematical Biosciences. 2020;325.DOI: 10.1016/j.mbs.2020.108364

121. Pandey KR, Subedee A, Khanal B, Koirala B. COVID-19 control strategies and intervention effects in resource limited settings: A modeling study. PLOS ONE. 2021;16(6):e0252570.DOI: 10.1371/journal.pone.0252570

122. Prabhakaran H. Spread of the Novel Coronavirus (SARS-CoV-2): Modeling and Simulation of Control Strategies. medRxiv. 2020:2020.05.11.20098418.DOI: 10.1101/2020.05.11.20098418

123. Proverbio D, Kemp F, Magni S, Husch A, Aalto A, Mombaerts L, et al. Dynamical SPQEIR model assesses the effectiveness of non-pharmaceutical interventions against COVID-19 epidemic outbreaks. PLoS ONE. 2021;16(5 May).DOI: 10.1371/journal.pone.0252019

124. Rusu AC, Emonet R, Farrahi K. Modelling digital and manual contact tracing for COVID-19. Are low uptakes and missed contacts deal-breakers? PLOS ONE. 2021;16(11):e0259969.DOI: 10.1371/journal.pone.0259969

125. Taboe HB, Salako KV, Tison JM, Ngonghala CN, Glèlè Kakaï R. Predicting COVID-19 spread in the face of control measures in West Africa. Mathematical Biosciences. 2020;328.DOI: 10.1016/j.mbs.2020.108431

126. Torneri A, Libin P, Vanderlocht J, Vandamme AM, Neyts J, Hens N. A prospect on the use of antiviral drugs to control local outbreaks of COVID-19. BMC Medicine. 2020;18(1).DOI: 10.1186/s12916-020-01636-4

127. Tuite AR, Fisman DN, Greer AL. Mathematical modelling of COVID-19 transmission and mitigation strategies in the population of Ontario, Canada. CMAJ. 2020;192(19):E497-E505.DOI: 10.1503/cmaj.200476

128. Wilson N, Blakely T, Baker MG, Eichner M. Estimating the risk of outbreaks of COVID-19 associated with shore leave by merchant ship crews: simulation studies for New Zealand. The New Zealand medical journal. 2021;134(1529):26-38

129. Wilson N, Baker MG, Blakely T, Eichner M. Estimating the impact of control measures to prevent outbreaks of COVID-19 associated with air travel into a COVID-19-free country. Scientific Reports. 2021;11(1):10766.DOI: 10.1038/s41598-021-89807-y

130. Yu Z, Zhu X, Liu X, Wei T, Yuan H-Y, Xu Y, et al. Reopening International Borders without Quarantine: Contact Tracing Integrated Policy against COVID-19. International Journal of Environmental Research and Public Health [Internet]. 2021; 18(14).

131. Bhadauria AS, Pathak R, Chaudhary M. A SIQ mathematical model on COVID-19 investigating the lockdown effect. Infectious Disease Modelling. 2021;6:244-57.DOI: 10.1016/j.idm.2020.12.010

132. Browne CJ, Gulbudak H, Macdonald JC. Differential impacts of contact tracing and lockdowns on outbreak size in COVID-19 model applied to China. Journal of Theoretical Biology. 2022;532:110919.DOI: https://doi.org/10.1016/j.jtbi.2021.110919

133. Gardner BJ, Kilpatrick AM. Contact tracing efficiency, transmission heterogeneity, and accelerating COVID-19 epidemics. PLOS Computational Biology. 2021;17(6):e1009122.DOI: 10.1371/journal.pcbi.1009122

134. Kempf P. Six scenarios of non-medical interventions in the SARS-CoV-2 epidemic. medRxiv. 2020:2020.05.25.20112532.DOI: 10.1101/2020.05.25.20112532

135. Kurita J, Sugawara T, Ohkusa Y. Effectiveness of COCOA, a COVID-19 contact notification application, in Japan. medRxiv. 2020:2020.07.11.20151597.DOI: 10.1101/2020.07.11.20151597

136. Li W, Gong J, Zhou J, Zhang L, Wang D, Li J, et al. An evaluation of COVID-19 transmission control in Wenzhou using a modified SEIR model. Epidemiology and Infection. 2021;149.DOI: 10.1017/S0950268820003064

137. Nuckchady DC. Impact of public health interventions on the COVID-19 epidemic: a stochastic model based on data from an African island. Archives of Clinical and Biomedical Research. 2022;6:517-35.DOI: 10.1101/2020.06.22.20134130

138. Nuzzo A, Tan CO, Raskar R, DeSimone DC, Kapa S, Gupta R. Universal Shelter-in-Place Versus Advanced Automated Contact Tracing and Targeted Isolation: A Case for 21st-Century Technologies for SARS-CoV-2 and Future Pandemics. Mayo Clinic Proceedings. 2020;95(9):1898-905.DOI: 10.1016/j.mayocp.2020.06.027

139. Siraj A, Worku A, Berhane K, Aregawi M, Eshetu M, Mirkuzie A, et al. Early estimates of COVID-19 infections in small, medium and large population clusters. BMJ Glob Health. 2020;5(9):e003055.DOI: 10.1136/bmjgh-2020-003055

140. Wang V. A Model for the Testing and Tracing Needed to Suppress COVID-19. medRxiv. 2020:2020.06.02.20120568.DOI: 10.1101/2020.06.02.20120568

141. Zu J, Li ML, Li ZF, Shen MW, Xiao YN, Ji FP. Transmission patterns of COVID-19 in the mainland of China and the efficacy of different control strategies: a data- And model-driven study. Infectious Diseases of Poverty. 2020;9(1).DOI: 10.1186/s40249-020-00709-z
